# A systematic identification of resistance determinants to antisense antibiotics suggests adaptation strategies dependent on the delivery peptide

**DOI:** 10.1038/s41467-026-76357-y

**Published:** 2026-08-12

**Authors:** Adam J. Mulkern, Thu-Hien Vu, Linda Popella, Tobias Kerrinnes, Svetlana Ðurica-Mitić, Manuel Halte, Kylee Dresbach-Hill, Anne-Catrin Uhlemann, Stephan Uphoff, Lars Barquist, Jörg Vogel, Marco Galardini

**Affiliations:** 1 Institute for Molecular Bacteriology, TWINCORE Centre for Experimental and Clinical Infection Research, a joint venture between the Hannover Medical School (MHH) and the Helmholtz Centre for Infection Research (HZI), Hannover, Germany; 2 Cluster of Excellence RESIST (EXC 2155), Hannover Medical School (MHH), Hannover, Germany; 3 Department of Biology, University of Oxford, Oxford, UK; 4 Institute of Molecular Infection Biology, University of Würzburg, Würzburg, Germany; 5 Cluster for Nucleic Acid Therapeutics Munich (CNATM), Munich, Germany; 6 Helmholtz Institute for RNA-based Infection Research (HIRI), Helmholtz Centre for Infection Research (HZI), Würzburg, Germany; 7 Department of Biochemistry, University of Oxford, Oxford, UK; 8 Department of Nutritional and Metabolic Biology, Columbia University Irving Medical Center, New York, NY USA; 9 Division of Infectious Diseases, Columbia University Irving Medical Center, New York, NY USA; 10 University of Toronto, Mississauga, ON Canada; 11 Faculty of Medicine, University of Würzburg, Würzburg, Germany

**Keywords:** Antimicrobial resistance, Experimental evolution

## Abstract

The rise of antimicrobial resistance (AMR) among human pathogens is a serious threat to global health, demanding novel treatment strategies. Antibiotics based on programmable antisense oligomers (asobiotics) offer an attractive solution, as their specificity can be quickly updated to target resistant bacteria. To understand the genetic architecture of resistance to asobiotics, we used laboratory evolution assays to identify mutations that decrease susceptibility to antisense peptide nucleic acids (PNAs) in four major gram-negative pathogens: *Escherichia coli*, *Klebsiella pneumoniae*, *Salmonella enterica*, and *Pseudomonas aeruginosa*. Reduced susceptibility depended on the cell-penetrating peptide (CPP) conjugated to the PNA, with reduced uptake emerging as a common adaptation only against the (KFF)_3_-K CPP. Accordingly, *sbmA* was consistently mutated across all species treated with (KFF)_3_-K-conjugated PNAs. We further identified mutations related to translation, peptide transport, and the cell envelope, generating new hypotheses on the cellular response to CPP-PNA conjugates. By contrast, for (RXR)_4_XB-*acpP* we observed only a modest resistance increase, driven by mutations in the PNA binding site that could be readily bypassed by changing the PNA sequence. These findings show that CPP identity strongly determines robustness against resistance evolution, and that laboratory evolution can illuminate the mechanisms of action of asobiotics.

## Abstract (web summary)

In this study, authors investigate the evolution of bacterial resistance to antisense peptide nucleic acid (PNA) antibiotics. They observe that this typically occurs mainly by reducing drug uptake via mutations in the carrier peptide’s transporter, while resistance to a different carrier peptide stayed low and is easily bypassed by tweaking the drug sequence.

## Introduction

Antimicrobial resistance (AMR) poses a significant threat to global public health, necessitating the urgent development of new therapeutic strategies^1^. The introduction of new conventional antibiotics is inevitably followed by the emergence and spread of resistance, which suggests that outpacing the evolution of resistance might not be a realistic strategy^2^. Sequence-based therapeutics, such as short antisense oligomers (ASO) could instead be quickly adapted to circumvent resistance determinants, thanks to their programmable nature, a feature that has been proven to be effective with mRNA vaccines updates^3^. In the context of antimicrobial therapy, asobiotics are a subclass of ASOs developed as antibacterials, designed to inhibit bacterial growth by targeting the mRNA of essential genes through sequence complementarity to the translation initiation region. ASOs are thus thought to modulate protein production by sterically blocking ribosome binding and preventing translation initiation^4–7^. Different ASO modalities have been developed^8^, with peptide nucleic acids (PNAs) and peptide phosphorodiamidate morpholino oligomers (PPMOs) being commonly used to target bacteria. PNAs are stabilized and protected from nuclease degradation by a neutrally charged pseudo-peptide backbone, while PPMOs are based on a phosphorodiamidate morpholino backbone that resists nuclease degradation. To facilitate their delivery into bacterial cells, PNAs/PPMOs are commonly conjugated to cell-penetrating peptides (CPPs). Commonly used CPPs include (KFF)_3_K and (RXR)_4_XB, which are thought to utilize distinct mechanisms for translocation across the bacterial membranes^9^. Previous studies have shown that the (KFF)_3_K-aided delivery of PNAs requires the inner membrane protein *S*bmA for transport^10–14^, which itself transports the unconjugated PNA after (KFF)_3_K has been degraded by a yet unidentified periplasmic protease. On the other hand, (RXR)_4_XB has been shown to operate through a membrane-potential dependent mechanism^15^.

Even though there have been substantial improvements in the development of asobiotics, for instance by defining the optimal ASO sequence characteristics^16^, a broad understanding of the mechanisms of resistance induced by treatment with asobiotics is currently lacking. Based on what is known about their mode of action, it can be expected that resistance mechanisms will fall in roughly three categories: reduced binding to the target site, impaired uptake, and other mechanisms. Mutations in the ASO binding region have indeed been proven to affect asobiotics efficacy, even though they have not yet been reported to spontaneously emerge upon treatment^17^. Spontaneous mutations affecting active uptake have instead been previously described, specifically loss-of-function mutations in *sbmA*, which encodes an inner membrane transporter required for the uptake of (KFF)_3_K-conjugated PNAs and PPMOs into bacterial cells; loss of SbmA function therefore renders bacteria less susceptible to these conjugates by preventing uptake^10,11^. If these resistance mutations were frequent across clinically relevant species and asobiotic constructs, it might be possible to focus on designing broad-spectrum treatments that induce these mutations with low propensity^18^; the empirical data are, however, currently lacking. Moreover, a systematic view of asobiotics resistance mutations would help generate new hypotheses about alternative uptake pathways and the cellular response to stress induced by interference with the translation of essential genes. This deeper understanding would, in turn, also allow for the design of asobiotics that are more resilient to the emergence of resistance.

In this study, we aimed to systematically investigate the resistance to CPP-PNA conjugates using in vitro experimental evolution. We tested four CPP-PNA conjugates across four clinically relevant gram-negative bacterial pathogens. We found that the choice of CPP has a large influence on the magnitude of induced resistance, and we determined which genetic variants are induced in response to treatment, some of which seem to be related to the cellular adaptation to the asobiotics mode of action. These results suggest that in order to develop programmable sequence-based antimicrobials that are resilient to the evolution of resistance, great consideration must be taken in selecting the most appropriate delivery mechanism.

## Results

### (KFF)_3_K induces the highest increase in resistance in a “drug selection ramp”

**Fig. 1:**
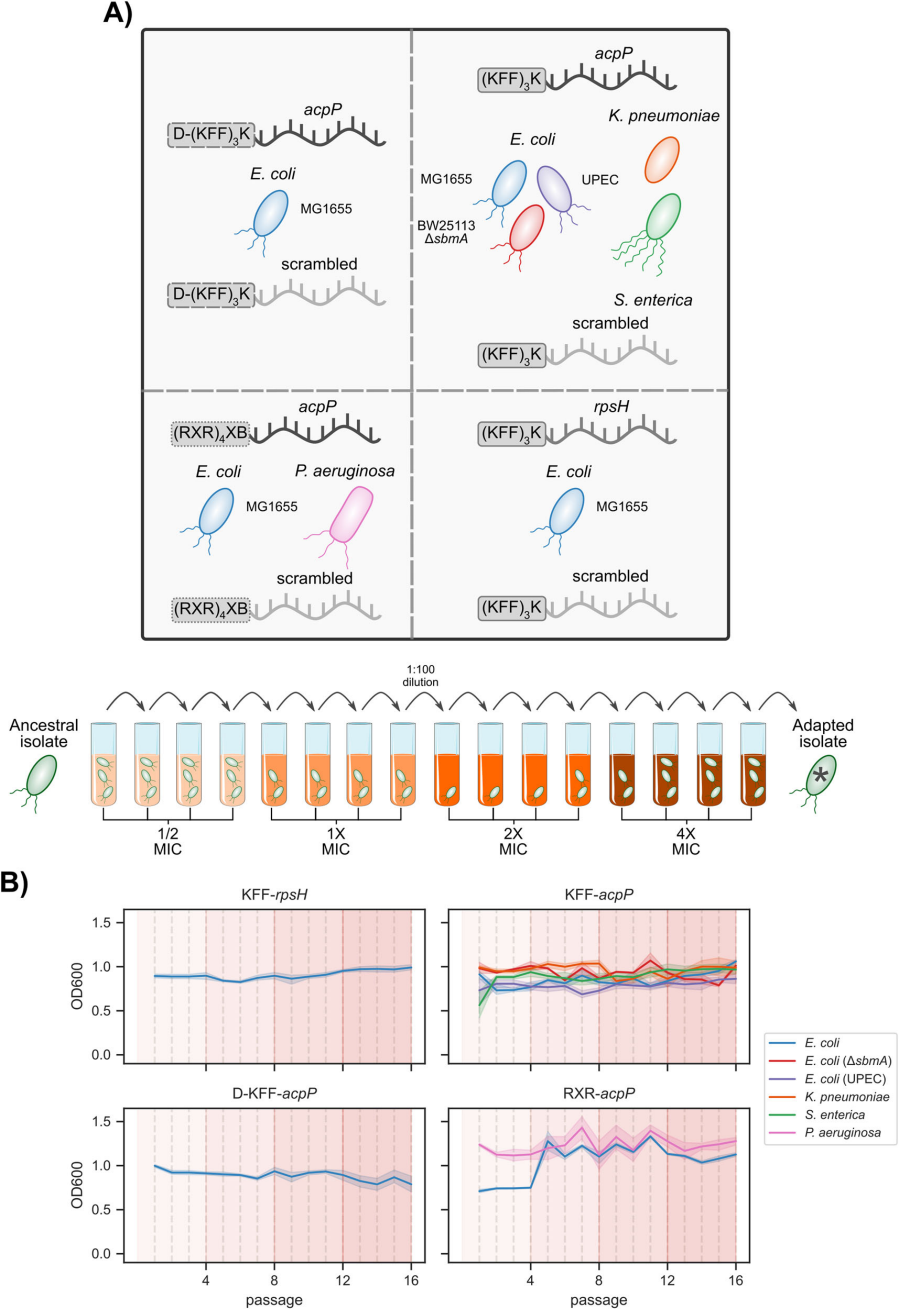
A drug selection ramp to induce resistance to asobiotics. **A** Schematic representation of the species and asobiotic constructs tested, as well as the serial passaging protocol. Test tube icon by Servier, via Bioicons (CC-BY 3.0). **B** Daily optical density (OD) measurements from the nine drug selection ramps; the solid line indicates the average OD over the 10 biological replicates, the shaded area the 95% confidence interval.

To induce resistance, we implemented a “drug selection ramp”, an in vitro laboratory evolution method that has been previously described^19,20^. This serial passage assay applies a gradual increase in selection pressure, starting from a sub-inhibitory concentration (half of the minimum inhibitory concentration (MIC) and doubling the concentration every four passages, reaching four times the MIC for the last passages (Fig. 1). We applied this assay on six individual strains belonging to four clinically relevant pathogen species: *E. coli*, *K. pneumoniae*, *S. enterica*, and *P. aeruginosa*. For *E. coli* we tested three distinct strains: the MG1655 reference, the KEIO *sbmA* knockout strain (with the BW25113 background, mutant ID JW0368), and the uropathogenic (UPEC) strain 536^21^. The Δ*sbmA* mutant was chosen for the role of the encoded inner membrane transporter in the uptake of (KFF)_3_K-conjugated PNAs into *E. coli*; knocking out this gene renders *E. coli* less susceptible to (KFF)_3_K-PNA treatment^11^. The Δ*sbmA* mutant could therefore provide insights into additional genes that influence PNA activity and are otherwise overlooked because of the predominant role of mutations in *sbmA*.

We tested four different ASO constructs, combining two distinct PNAs, targeting the *rpsH* and *acpP* essential genes, respectively, with three CPPs; we used (KFF)_3_K in its original L-form, its D-form (D-(KFF)_3_K), and (RXR)_4_XB, termed as KFF, D-KFF, and RXR hereafter (Fig. 1A). We tested KFF-*acpP* on all strains except *P. aeruginosa*, for which we tested a cognate RXR-*acpP* construct. *P. aeruginosa* lacks a *sbmA* homolog, very likely accounting for the ineffectiveness of KFF for PNA delivery for this species^22^. In contrast, all four ASO constructs were tested against the *E. coli* MG1655 reference strain to directly compare them: KFF-*acpP*, D-KFF-*acpP*, KFF-*rpsH*, and RXR-*acpP*. Ten biological replicates were performed for each asobiotic / strain combination to better evaluate the variance in the induced resistance phenotype. As a control, we also tested a scrambled PNA conjugated with each of the three CPPs, which indicated that they had no detectable impact on the strains we tested.

**Fig. 2:**
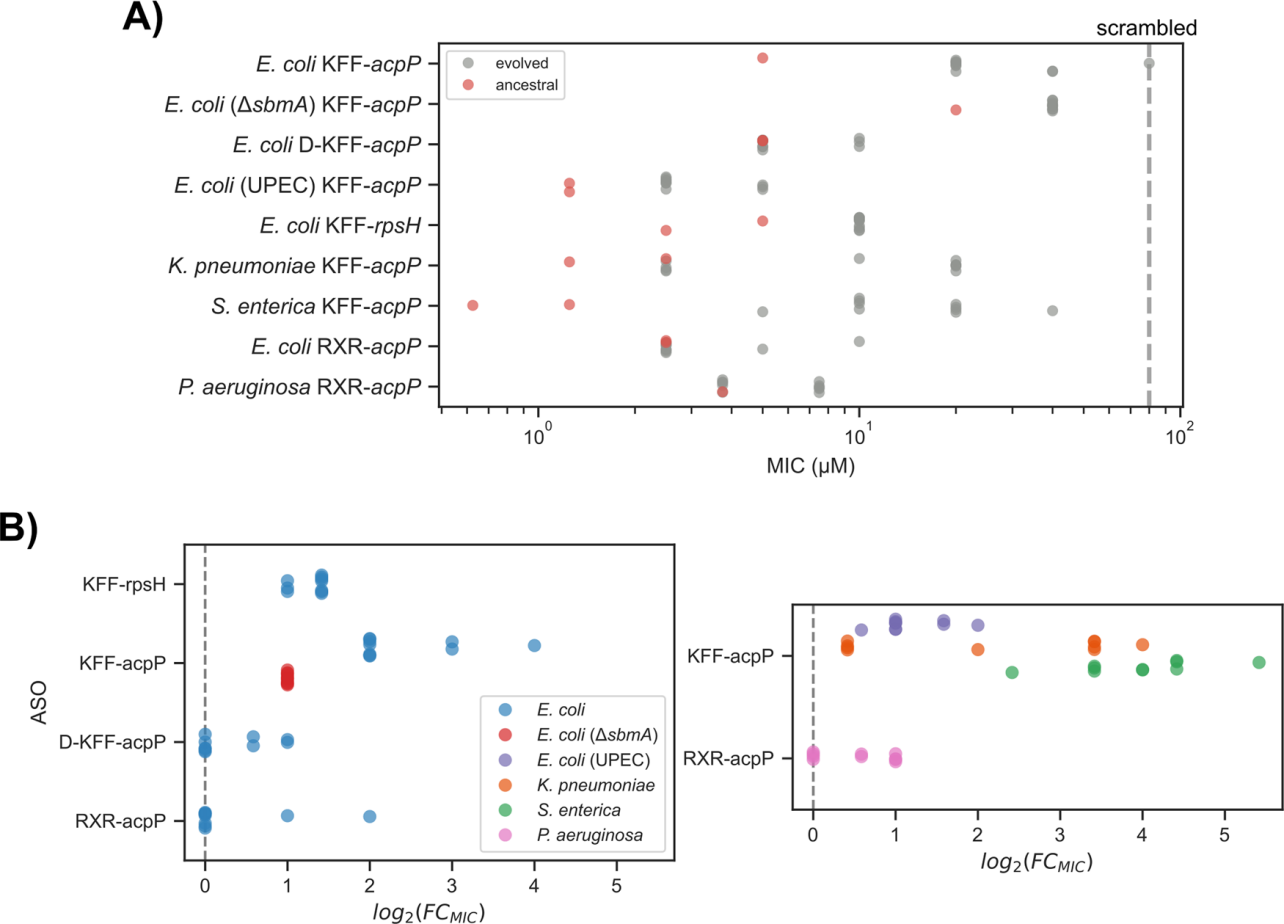
Changes in asobiotics sensitivity across species and strains. **A** Absolute values for the minimum inhibitory concentration (MIC) for all ancestral isolates (red, 2 replicates) and evolved samples (gray, 10 replicates). The vertical gray dashed line indicates the MIC of the samples adapted to the scrambled asobiotics across all species and constructs. **B** Fold-change increase in minimum inhibitory concentration (MIC) after the completion of the selection ramp with respect to the ancestral isolate. Each dot indicates the fold-change for a single biological replicate, vertical gray dashed line represents ancestral MIC values. The left sub-panel shows the measurements for the two *E. coli* laboratory strains only, other species are shown in the right sub-panel.

We observed consistent levels of growth during each of the 16-daily 1:100 passages of the drug selection ramp, with limited variability across replicates (Supplementary Fig. 1), suggesting that each isolate may have developed resistance to the asobiotic construct by the end of the ramp (Fig. 1B). We also observed similar growth dynamics for the scrambled controls (Supplementary Fig. 2). We confirmed that on average all drug selection ramps induced resistance by performing susceptibility assays, which revealed increased MIC values between the ancestral and adapted populations (Fig. 2A). As expected, we recorded no change in MIC values for the strains adapted to the scrambled controls, for which we observed absolute values > 80 µM (Fig. 2A). We however observed a relatively wide range of absolute MIC values across the ancestral isolates (Supplementary Data 1): *S. enterica* treated with KFF-*acpP* was the most sensitive (average 1.88 µM), while the Δ*sbmA* mutant was, as expected, the most resistant to KFF-*acpP* (average 20 µM), which was four times the MIC value of *E. coli* MG1655 treated with KFF-*acpP* (average 5 µM). We observed a significant fold-change increase in MIC (one-sample Wilcoxon signed-rank test *p*-value < 0.05) for all combinations of species and asobiotics, with the exception of *E. coli* MG1655 adapted to D-KFF-*acpP* and RXR-*acpP*. We observed the highest increase in MIC for *S. enterica* adapted to KFF-*acpP*, with an average fold increase of 17.6. On the other hand, we observed the lowest increase for *E. coli* MG1655 adapted to D-KFF-*acpP*, with an average fold increase of 1.3. Strikingly, for *E. coli* MG1655 adapted to RXR-*acpP* we did not record any increase in MIC with respect to the ancestral isolate for 8 out of the 10 adapted populations (Fig. 2B, and Supplementary Data 1). When focusing on *E. coli* MG1655, which was tested on all four asobiotic constructs, we observed a clear difference in MIC increase based on the CPPs used, with the two KFF constructs inducing a higher level of resistance (mean MIC fold change of 2.5 and 6 for KFF-*rpsH* and KFF-*acpP*, respectively) when compared to both D-KFF-*acpP* and RXR-*acpP* (1.3 and 1.4 average increase in MIC, respectively). For these last two constructs in particular (i.e., D-KFF-*acpP* and RXR-*acpP*), we observed no significant difference when applying a Mann-Whitney U test (bonferroni-corrected *p*-value > 0.05). These results prove (KFF)_3_K to be a less resilient choice of CPP for asobiotic delivery, which is in line with previous reports showing its low stability and its dependency on *sbmA*-mediated active uptake^13^. We further confirmed this observation by noting that the MIC increase of the Δ*sbmA* mutant was on average higher than that we observed for both D-KFF-*acpP* and RXR-*acpP* (2 versus 1.3 and 1.4, respectively). Therefore, this mutant has additional capacity to increase its resistance on top of the already existing *sbmA* gene deletion. We also observed that selecting a target gene and a PNA sequence with the same CPP affected the extent of acquired resistance. On average, *E. coli* MG1655 adapted to KFF-*acpP* had a higher fold increase in MIC (6-fold) when compared to KFF-*rpsH* (2.5-fold, Fig. 2B).

### (KFF)_3_K asobiotics induce genetic variants in *sbmA* in the majority of samples across species

**Fig. 3:**
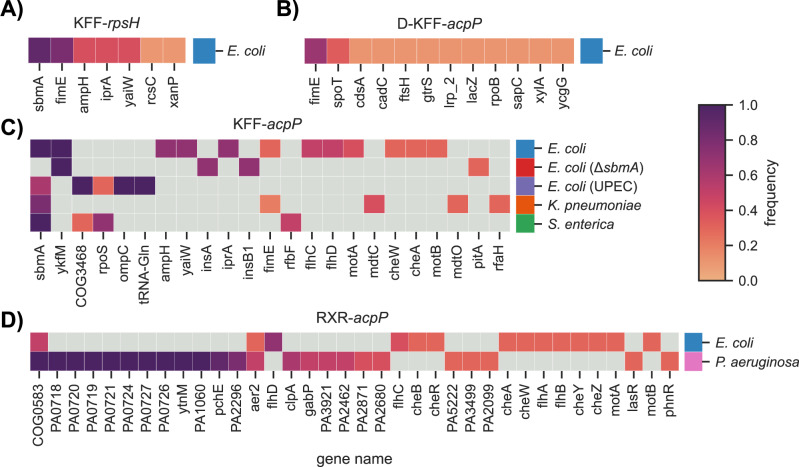
Common genetic variants induced during the drug selection ramps. The heatmaps indicate the proportion of replicates for each strain / asobiotic combination (**A** for KFF-*rpsH*, **B** for D-KFF-*acpP*, **C** for KFF-acpP, **D** for RXR-*acpP*) for which we observed genetic variants in each gene. Gray boxes indicate that no variant was observed in the corresponding gene. For KFF-*acpP* and RXR-*acpP*, which each have a list of genes with at least one sample showing a mutation, only genes with a variant in at least three replicates are shown. Genes are sorted by their average frequency across all species, with high to low frequency sorted from left to right.

To uncover the genetic basis of the observed resistance, we carried out whole-genome sequencing of both the ancestral and adapted experimental populations (Supplementary Data 2). In order to avoid considering variants induced by serial passaging or the non-targeted effect of scrambled asobiotics, we also sequenced the scrambled controls (Supplementary Data 3 and Supplementary Fig. 3). On average, we counted 34.9 genetic variants for each biological replicate (12 genetic variants on average for scrambled controls). *P. aeruginosa* displayed the highest number of variants (199.5 for each replicate on average), while for *E. coli* exposed to the D-KFF-scrambled control asobiotic had the highest number of variants (62 for each replicate on average). We recorded the “synonymous SNP” (single-nucleotide polymorphism) as the most common category of genetic variant across all samples (average of 15 SNPs per sample), while for the scrambled controls the most prevalent variants were large deletions (average of 8 large deletions per sample). We calculated the proportion of samples in which each gene was found to be mutated across the ten biological replicates for each strain / asobiotic combination. This metric was used to identify the most likely determinants of resistance for each asobiotic construct (Fig. 3A–D and Supplementary Fig. 4). For this analysis we excluded synonymous SNPs, variants in intergenic regions, and those in known pseudogenes.

For all the species adapted to KFF-*acpP*, we observed a high (at least six replicates) proportion of samples with likely impactful genetic variants in the *sbmA* gene, which is essential for the transport of KFF-conjugated PNAs into the cell. We observed a similarly high proportion (9 replicates) of samples with variants in the *sbmA* gene for *E. coli* MG1655 adapted to KFF-*rpsH*, suggesting that resistance to KFF in this strain outweighs that to the PNA itself. This result extends previous reports on the role of mutations in *sbmA* in resistance to KFF-PNAs beyond a single strain of *E. coli* and extends it to *S. enterica* and *K. pneumoniae*^11^. For *E. coli* MG1655 we observed a high proportion of samples with variants in *yaiW* when adapted to both KFF-*acpP* (7 replicates) and KFF-*rpsH* (4 replicates); *yaiW* is co-transcribed with *sbmA*, encodes for an outer membrane protein, and its deletion mutant has been shown to affect the sensitivity to antimicrobial peptides, which overall may indicate a functional relationship with the better characterized SbmA^23^. We indeed validated that the double *sbmA*/*yaiW* deletion confers higher resistance to KFF-*acpP* (see next section). Additionally, variants in the *ykfM* gene were consistently identified across all replicates of both *E. coli* MG1655 and Δ*sbmA* adapted to KFF-*acpP*. This uncharacterized transmembrane gene has been linked to alterations in response to chemical and physical stresses^24^.

It has been previously shown how SbmA transports PNAs from the periplasmic space into the cytoplasm after KFF has been proteolytically degraded, and how its D-form (D-KFF), by virtue of its resistance to proteolysis, is not dependent on this transport mechanism^11^. Consistent with this model, we did not observe any mutation in *sbmA* when we treated *E. coli* with D-KFF-*acpP* (Supplementary Fig. 5). We instead observed that D-KFF-*acpP* induced variants in the *spoT* gene in *E. coli* (3 replicates), possibly linking the SpoT-dependent stress response to fatty acid metabolism, which is directly targeted by the *acpP* PNA^25^. We further identified variants induced in *xanP (*encoding for a nucleobase transporter^26^) in *E. coli* MG1655 treated with KFF-*acpP* (1 replicate, Supplementary Fig. 6), KFF-*rpsH* (1 replicate, Supplementary Fig. 7), and RXR-*acpP* (1 replicate, Supplementary Fig. 8), as well as in the Δ*sbmA* mutant treated with KFF-*acpP* (2 replicates, Supplementary Fig. 9). In *S. enterica*, the most common mutations were found in *rpoS* (8 replicates), which encodes the RNA polymerase sigma factor, and in *rfbF* (5 replicates) (Supplementary Fig. 13), a gene involved in the biosynthesis of the LPS O-antigen in the outer membrane, which has been shown to inhibit PNA efficacy in *E. coli*^27^. In *K. pneumoniae*, we observed mutations in *mdtC* in 4 replicates. This gene is part of the *mdtABC*/*tolC* multidrug efflux pump and confers resistance to several antimicrobials^28^. Even though chemical inhibitors of the pump activity have shown no impact on PNA efficacy^27^, the *bamB*/*tolC* double mutant was found to be more sensitive to KFF-*acpP* in *E. coli*^29^. Finally, both D-KFF-*acpP* and RXR-*acpP* constructs did not induce a large increase in MIC in most samples (Fig. 2). Therefore, we hypothesize that the variants observed in a high proportion of samples observed may not be directly related to resistance to these ASO constructs and might represent “passenger” mutations instead.

### Private mutations are associated with variability in the induced resistance to asobiotic treatment

**Fig. 4:**
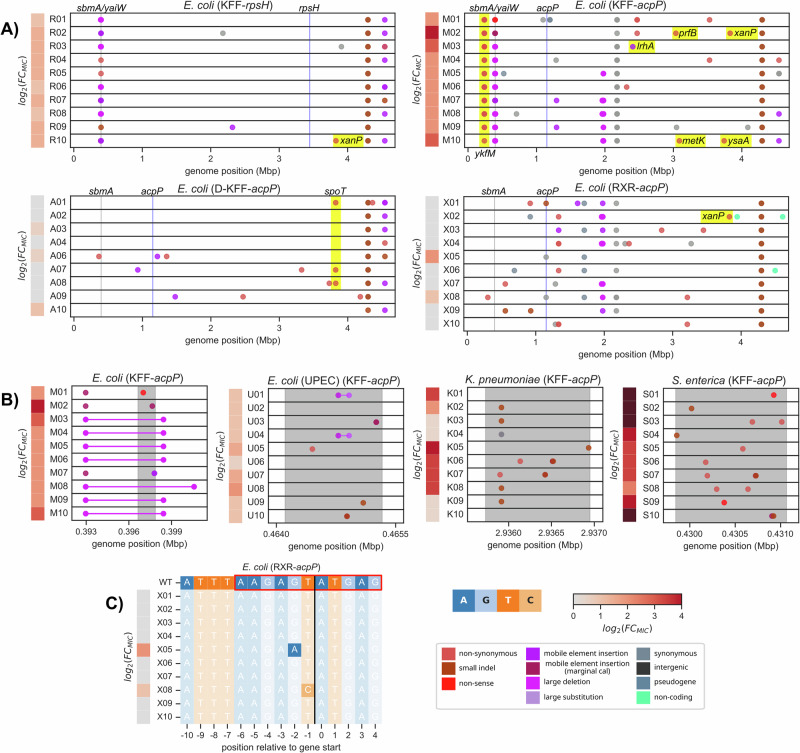
A detailed view on the genome-wide distribution of genetic variants induced by the drug selection ramps. **A** Genome-wide distribution of genetic variants in each biological replicate for *E. coli* MG1655 after exposure to each of the four asobiotic constructs; the MIC fold-change with respect to the ancestral isolate is shown in gray-to-red heatmaps on the left. The gray and blue vertical lines indicate the location of the *sbmA* and PNA target genes (*acpP* or *rpsH*), respectively. Other genes discussed in the main text are highlighted in yellow. The same visualization for the other five species/ASO constructs can be found in Supplementary Fig. 10. **B** Genetic variants in the *sbmA* gene induced by treating *E. coli* MG1655, UPEC 536, *K. pneumoniae* or *S. enterica* with KFF-*acpP*. The gray shaded area in each panel indicates the location of *sbmA*’s coding sequence. Each subpanel has a different genomic window size in order to accommodate the variants in and around *sbmA*. **C** Rare genetic variants in *acpP* induced within the RXR-*acpP* targeting window in *E. coli* MG1655; the red box indicates the location of the PNA binding site. The two replicates with SNPs within the target sequence are highlighted, together with their MIC fold-change increase. The vertical black line indicates the start of *acpP*’s coding sequence.

Given the variability in the MIC fold changes across the different biological replicates, we next focused on the differences between individual samples adapted to the same asobiotic construct. We looked in particular at *E. coli* MG1655, which was adapted to all four asobiotics, to study the joint variability in phenotypic and genotypic adaptation (Fig. 4A and Supplementary Figs. 5–9, 11–14). For KFF-*rpsH* we observed a relatively uniform increase in MIC across all replicates (2.5 fold-change over the ancestral on average), and with all isolates encoding a variant in *sbmA*. Populations of *E. coli* MG1655 adapted to KFF-*acpP* had both higher average MIC increases (6-fold over the ancestral isolate) and greater variance, with three replicates increasing their MIC relative to the ancestral isolate more than 8-fold. Since all samples developed similar *sbmA* genetic variants, we focused on variants exclusive to three samples with higher MIC increase (Supplementary Fig. 6). For sample M03, which showed an 8-fold increase in MIC we observed a private mobile genetic element insertion in the *lrhA* gene, a probable transcriptional regulator. For sample M10, which also showed an 8-fold increase in MIC, we observed two private non-synonymous SNPs in the *metK* (V231F) and *ysaA* (W3R) genes, both of which have no known involvement in resistance to asobiotics or the KFF CPP. Lastly, for sample M02 we observed a 16-fold increase in MIC, the above-mentioned variant in *xanP*, and a non-synonymous SNP, not found in any other sample, in codon 246 (T246S) of the *prfB* gene. *prfB* encodes for the peptide chain release factor RF2, which, among other functions, interacts with ArfA to recover stalled ribosomes^30,31^. Mutations at position 246 in particular have been shown to increase termination efficiency^32,33^. Even though the mechanism of action of asobiotics is thought to be the prevention of ribosome binding through steric interference, we found the function of this gene in general, and of mutations at this position in particular, compelling, also in light of the absence of other unique variants. Interestingly, in UPEC adapted to KFF-*acpP*, we have observed a variant in the related gene *prfA* (Supplementary Fig. 12), specifically a C127F substitution, which further highlights the potential relevance of *prf*-related genes in adaptation to asobiotics treatments. We indeed validated the ability of the *prfB* variant to confer elevated resistance to KFF-*acpP* (see next section).

Even though all three tested species adapted to KFF-*acpP* developed mutations in *sbmA*, we observed some variability in the types of induced variants across replicates (Fig. 4B). For *E. coli* MG1655 we observed large (average 5771 bp) deletions mediated by the IS3 insertion sequence in 7 replicates, 5 of which had the same length (5465 bp) and start and end position on the chromosome. We observed the exact same IS3-mediated deletions in 4 replicates of *E. coli* MG1655 adapted to KFF-*rpsH* (Supplementary Fig. 7). The deletion variants induced by KFF-*acpP* in UPEC 536 were in contrast rarer (2 replicates) and shorter (both 134 bp). At the other end of the spectrum, we noted that variants induced in both *K. pneumoniae* and *S. enterica* were all short variants such as non-synonymous SNPs (3 replicates for *K. pneumoniae*, 5 for *S. enterica*), non-sense SNPs (2 replicates for *S. enterica*), and small insertion/deletions (InDels, 7 replicates for *K. pneumoniae*, 4 for *S. enterica*).

### A relatively rare mutation within the PNA target window is associated with reduced sensitivity to RXR-*acpP* in *E. coli*

Since RXR-aided PNA uptake is independent of any active transporter machinery, there might be no direct way of developing resistance against the RXR CPP, which likely explains the less frequent mutations across replicates and the average low increase in MIC. As reported above and in Fig. 1, we observed that only two *E. coli* MG1655 populations developed a measurable increase in MIC when compared to the ancestral isolate adapted to the RXR-*acpP* construct. The most notable genetic variants (Supplementary Fig. 8) differentiating these samples from the remaining eight was the presence of two SNPs in the center of the RXR-*acpP* binding site, i.e. at position −1 (T > C) and −2 (G > A) for sample X08 (2 fold MIC increase) and X05 (4 fold MIC increase), respectively (Fig. 4C). While up to two mismatches on the 5’ or 3’ binding target of PNA and PPMO-based asobiotics do not completely abolish its binding capacity, mismatches in the central binding region substantially impair PNA binding^16,17,34^. In both samples, we observed a relatively low frequency ( ~ 30%) of these SNPs, which may explain the relatively small increase in MIC. From this result, we can conclude that *E. coli* (strain MG1655 in particular) seems to be unable to successfully escape the detrimental effects of RXR- or (D)-KFF-mediated delivery of the *acpP* PNA.

### Genetic reconstruction confirms two mechanistically distinct routes to KFF-*acpP* resistance

To causally validate candidate resistance determinants identified from population-level sequencing, we isolated single clones from two evolved *E. coli* MG1655 populations and reconstructed the variants they carried in a clean MG1655 background. We focused on two mechanistically distinct classes of resistance determinant: loss-of-function of the *sbmA/yaiW* operon predicted to impact CPP-dependent PNA uptake, and the *prfB* T246S non-synonymous variant identified in sample M02 (Fig. 4A), predicted to affect translation termination and confer resistance through an uptake-independent mechanism. Each variant was reconstructed independently in the MG1655 background using a two-step scarless Lambda-Red/I-SceI allelic replacement method, with *prfB* (an essential gene in *E. coli*) reconstructed via a hybrid Lambda-Red/P1 co-transduction strategy at the linked *idi* locus (see Materials and Methods). MIC assays against KFF-a*cpP* confirmed that both variants are individually sufficient to confer resistance in isolation. The reconstructed Δ*sbmA*/*yaiW* mutant displayed an MIC of >80 µM against KFF-*acpP*, a > 16-fold increase over the ancestral MG1655 MIC of 5 µM, consistent with loss of KFF-PNA inner membrane translocation. The *ΔsbmA*/*yaiW* mutant also showed up to a > 2-fold increase over the *E.coli* treated with KFF-*acpP* evolved lines with no identified *yaiW* variant; 2-fold increase with respect to samples M01 and M07, and no MIC increase for sample M02. The reconstructed *prfB* T246S mutant displayed a MIC of 20 µM, a 4-fold increase with respect to the ancestral isolate, and a similar increase as we had recorded in 7 of the 10 *E. coli* lines adapted to KFF-*acpP*, demonstrating that a single substitution in a peptide chain release factor confers resistance in the absence of any uptake-associated variant. Both reconstructed MICs fall within the range observed in the evolved MG1655 KFF-*acpP* populations (Fig. 2), identifying loss of SbmA-mediated uptake and modulation of translation termination as two distinct routes to KFF-*acpP* resistance.

## Discussion

A crucial step in developing new antimicrobial compounds is identifying the resistance mechanisms that will inevitably emerge once these treatments are being used at scale^35^. This step is important not only for forecasting the evolution of resistance but also for elucidating the mechanism of action of the new compounds. The selective pressure exerted by the antimicrobial is likely to elicit mutations in genes directly involved in the drug's action and the cell's response to it. In the case of asobiotics, this is of particular importance, as empirical data on their uptake, precise mode of action, and impact on cellular processes is still scarce. Additionally, data on whether clinically relevant species respond similarly to CPP-PNA conjugates are currently lacking.

In this study, we have for the first time used a systematic approach to identify the genetic determinants for asobiotics resistance across CPP-PNA conjugates and clinically relevant bacterial gram-negative species (*E. coli*, *S. enterica*, *K. pneumoniae*, and *P. aeruginosa*). An important observation from our study is the high prevalence of mutations in the *sbmA* gene when using KFF-conjugated PNAs. While these mutations had been previously reported for *E. coli*^11^, here we have shown how this gene is also prevalently mutated in two other clinically relevant species, such as *S. enterica* and *K. pneumoniae*. Beyond *sbmA*, in *E. coli* treated with KFF-*acpP* we frequently observed variants in *yaiW*, which is co-transcribed with *sbmA* and is localized in the outer membrane; its role in resistance to antimicrobial peptides might give it a role in the uptake of KFF-conjugated PNAs^23^. The downstream gene product YaiW is, in fact, a palmitate-modified surface-exposed outer membrane lipoprotein^23^, indicating a possible initial entry mechanism for the KFF-PNAs into the periplasmic space, followed by a SbmA-dependent transport into the cytoplasm. Through the generation of a clean double mutant, we have shown how a *yaiW* deletion might confer up to a 2-fold increase in MIC compared to a single *sbmA* deletion. Further work will be needed to elucidate the precise molecular mechanism that leads to this increase in resistance, and whether deleterious variants in this gene also confer resistance in other Gram-negative species. Other prevalent mutations of note include the *ykfM* gene, encoding an uncharacterized transmembrane protein, suggesting an additional possible asobiotic transport mechanism and therefore a potential role in resistance. However, since knockout mutants of this gene show changes in their chemical and physical stress response pathways^24^, this mutation could instead be influencing PNA efficacy indirectly. Furthermore, we observed other species-specific mutations induced by treatment with KFF-*acpP*, including the glutamine-tRNA gene in UPEC 536, *rfbF* in *S. enterica*, and *mdtC* in *K. pneumoniae*. Mutations that might alter translation rates and efficiency, which is a plausible consequence of a mutation in a tRNA gene, and more so in the peptide release factors *prfA* and *prfB*, might ameliorate the reduced rate of translation of the target gene. Both *prfA* and *prfB* are known to influence translation by rescuing stalled ribosomes^30,31,36^; we can therefore hypothesize that mutations in these two genes can confer resistance to PNAs via two mechanisms. The first one could be of an indirect nature, via an overall increased rate of translation, which would also affect the targeted essential gene, thus counteracting the effect of the PNA’s steric interference. The second would be via direct interaction with the ribosome subunits that fail to form a functional complex on the target mRNA due to PNA binding. Apart from validating the role of the *prfB* mutation in asobiotics resistance, the current available information is insufficient to clarify which of these hypotheses are correct, if any. Regarding mutations in *rfbF*, we hypothesize that they could result in significant alterations to the outer membrane structure, thus potentially impacting the ability for CPPs to bind and penetrate the cell membrane^27^, and in turn, inhibit PNA delivery. Again, these altered membrane properties could have further downstream impacts on the susceptibility of the pathogen not only to asobiotics, but also to other antimicrobials. Similarly, we have observed frequent mutations in *mdtC*, which is part of the *mdtABC*/*tolC* multidrug efflux pump; as *tolC*/*bamB* double knockout mutants in *E. coli* have been shown to have higher susceptibility to KFF-*acpP*, we can anticipate that investigating efflux pumps more closely might further elucidate KFF-PNAs uptake^29^.

It has been previously demonstrated that introducing mutations in the central asobiotic binding site results in its reduced efficacy^16,17^. To the best of our knowledge, it has not been previously reported to happen spontaneously upon treatment. We observed two instances of mutations in the central portion of the asobiotic *acpP* binding site when treating *E. coli* MG1655 with RXR-*acpP*, which were coincidentally the only samples treated with this construct to show a measurable increase in their MIC. This striking correlation, and existing in vitro data on this very genomic region^16^, leads us to hypothesize with relatively high confidence that these mutations are the sole cause of the increase in resistance for these two samples. We also use the observation that mutations in the asobiotic binding site were induced in only two samples over the total of 90 evolved lines to hypothesize that mutations in these highly conserved binding regions might incur a fitness cost; this, in turn, makes them less likely to emerge than the other mutations we have observed. Consistent with this interpretation, attempts to reconstruct the *acpP* target-site SNP via a modified CRISPR-Cas9 editing system^37^ in a clean MG1655 background encountered repeated technical barriers attributable to sensitivity of *acpP* to reduced expression, as *acpP* is among the most stringently required essential genes in *E. coli*, where even small reductions in transcript level substantially impair growth^38^. Even if the propensity of these variants to emerge was eventually found to be higher in more realistic treatment scenarios, they could be circumvented with relative ease through an update of the PNA sequence, thus making asobiotic a treatment that is resilient to the evolution of resistance.

As we had expected based on theoretical considerations, we have observed that asobiotic treatment induces mutations affecting both uptake and the target sequence itself. It is also worth noting that the spectrum of resistance determinants recovered in any evolution experiment is shaped by the selection regime applied, and that the gradual concentration ramp used here may favor different mutational routes than would, for example, abrupt high-dose exposure or a fluctuating treatment. For some of the remaining variants, we could make hypotheses on their role in resistance (e.g., outer membrane composition and efflux), but perhaps more intriguingly on the actual mode of action of this new class of antimicrobials (i.e., rescue of stalled ribosomes). Future work should be directed at further characterizing these variants to validate these hypotheses and clarify the molecular mechanisms behind asobiotics action and cellular response, with a special focus on their evolutionary resilience.

## Methods

### Bacterial strains

The following strains were used in this study: *Escherichia coli* MG1655 (JVS-5709; NCBI GenBank: U00096.3), *Pseudomonas aeruginosa* PAO1 (JVS-11761; NCBI RefSeq: NC_002516.2), *Klebsiella pneumoniae* ICC8001 (kindly provided by Joshua Liang Chao Wong and Gad Frankel, Imperial College London; JVS-13403; NCBI GenBank: CP009208.1), uropathogenic *E. coli* 536 (UPEC 536; JVS-12054; NCBI GenBank: CP000247.1). All strains were streaked on Luria-Bertani (LB) agar plates to obtain single colonies and cultured in non-cation adjusted Mueller-Hinton Broth (MHB, BD Difco™, Thermo Fisher Scientific), at 37 °C and 220 rpm shaking.

### Peptide nucleic acids (PNAs)

Peptide-conjugated PNAs (PPNA), directly linked to either (KFF)_3_K, D-(KFF)_3_K or (RXR)_4_XB, were purchased from Peps4LS GmbH (Heidelberg, Germany) or provided by W. Tegge, B. Kornak, and M. Brönstrup at the Helmholtz Center for Infection Research (HZI, Braunschweig). The constructs’ quality and purity were verified by mass spectrometry and HPLC, ensuring a purity level above 98% (Supplementary Notes). PPNAs were dissolved in nuclease-free water and heated at 55 °C for 5 min before use. Concentrations were measured using a NanoDrop spectrophotometer (A260 nm; ThermoFisher, U.S.) and calculated based on the extinction coefficient^39^. The PPNAs were stored at –20 °C and reheated at 55 °C for 5 minutes before preparing the working dilutions. Throughout, low retention pipette tips and low binding Eppendorf tubes (Sarstedt, Germany) were used. The PNAs sequences are listed in Supplementary Data 4.

### Determination of minimal inhibitory concentration (MIC) and minimal bactericidal concentration (MBC)

The MIC values were determined using a modified broth microdilution method^40^, following the guidelines of the Clinical and Laboratory Standards Institute. An overnight bacterial culture was diluted 1:100 in fresh Mueller-Hinton Broth (MHB) and cultivated to an optical density (OD600) of 0.5. This culture was further diluted in MHB to achieve a final concentration of approximately 10^5^ cfu/ml. The diluted bacterial solution was then combined with 10x PPNA working solutions in a 96-well plate (Thermo Fisher Scientific, U.S.), adjusting a final volume of 100 µl per well. Growth was monitored using a BioTek Epoch2NS Microplate Spectrophotometer (Agilent, U.S.), measuring OD600 every 20 min for 24 h at 37 °C with continuous double-orbital shaking (237 cpm). The MIC was defined as the lowest PPNA concentration that inhibited visible growth (OD600 < 0.1).

To determine the MBC, immediately following the MIC assessment, the entire contents of wells containing ½x, 1x and 2x MIC were plated onto LB agar. These plates were incubated at 37 °C for 18–20 h, and bacterial growth was recorded to identify the lowest PPNA concentration preventing regrowth. To detect potential slow-growing colonies, the plates were further incubated at room temperature for an additional 72 h.

### Experimental evolution

Resistance was induced by subjecting bacterial strains to progressively increasing concentrations of PPNA over 16 daily passages in LB broth, distinct from the MHB-based MIC determination described above. Initial susceptibility values determined by MIC were used to design a modified ‘evolutionary ramp’ protocol^19,20^, with PPNA concentrations increased every four passages from ½x MIC to 4x MIC. The passages were conducted in 96-well microplates (Greiner Bio-One, Austria) with 10 biological replicates for each strain and PPNA combination (150 µl total well volume). Plates were incubated for 22 hours at 37 °C, and overnight cultures were diluted 1:100 into fresh LB broth medium containing the relevant PPNA concentration for the next passage. Optical density (OD600nm) was measured at the end of each passage. Parallel evolution experiments using scrambled-sequence PNA controls conjugated to each CPP were performed under identical conditions (Supplementary Fig. 2). All samples, including both control and treated strain groups, were stored in 25% glycerol at −80 °C.

### Reconstruction of resistance-associated variants in MG1655

The Δ*sbmA*/*yaiW* deletion and *prfB* T246S substitution identified in evolved *E. coli* MG1655 lineages were reconstructed in the parental MG1655 background using a two-step scarless Lambda-Red/I-SceI allelic replacement method^41,42^ with the temperature-sensitive helper plasmid pWRG730. A kanamycin-resistance/I-SceI (KanSceI) cassette was first inserted at the target locus by Lambda-Red recombination and selected on kanamycin (50 μg/ml). In a second step the cassette was replaced by the desired allele, and clones retaining the cassette were eliminated by I-SceI-induced double-strand cleavage upon induction with anhydrotetracycline (AnTc, 100 ng/ml). All recombineering steps were performed at 30 °C to maintain pWRG730, and verified clones were restreaked twice at 42 °C to cure the helper plasmid.

For the Δ*sbmA*/*yaiW* reconstruction, the chromosomal region 392,966–400,583 of MG1655 (RefSeq NC_000913.3), spanning *sbmA* and *yaiW*, was replaced with the KanSceI cassette using primers AMH7 / AMH8, then exchanged for a PCR product amplified from the M08 evolved clone using primers AMH9 / AMH10. Insertion and replacement were verified by PCR with primers AMH11 / AMH12 and by Sanger sequencing (Plasmidsaurus).

Because *prfB* is essential in *E. coli*, the *prfB* T246S substitution was introduced by a hybrid strategy combining KanSceI insertion at the linked *idi* locus with P1 transduction from the M02 evolved clone. A KanSceI cassette amplified with primers AMH41 / AMH42 was inserted at the *idi* locus (3,033,064–3,033,583), generating strain AMH4 (MG1655 Δ*idi*::KanSceI / pWRG730). P1 phage was prepared on the M02 donor and used to transduce AMH4; transductants were recovered in LB at 30 °C, induced with AnTc to drive I-SceI counter-selection, and plated on chloramphenicol plus AnTc. Colonies that had lost the *idi*::KanSceI cassette (PCR screen with primers AMH43 / AMH44) were then sequenced at the *prfB* locus using primers AMH5 / AMH6 to confirm the T246S substitution. Primer sequences and strain designations are provided in Supplementary Data 5.

### DNA isolation, sequencing, and data analysis

To assess genetic variations in microbial populations from experimental evolution, DNA was extracted from the bulk liquid culture of each 10 replicated treatment groups and one control ancestral group for every combination of PPNA and strain at the final collection point (passage 16). The DNeasy Blood & Tissue Kit (Qiagen, Germany) was used for genomic DNA extraction, following the manufacturer’s protocol for gram-negative bacteria. DNA concentration and purity were measured using a NanoDrop spectrophotometer (dsDNA, A_260_/A_280_ ratio). Library preparation using standard Illumina protocols and sequencing using the Illumina HiSeq 4000/PE150 platform were performed at Novogene (Cambridge, UK). Variant populations were detected using breseq (v.0.39.0)^43^, a pipeline tool developed for detecting mutations in laboratory-evolving microorganisms. The analysis was performed using the polymorphism mode to detect variants present in a non-clonal bacterial population. Mutations were filtered based on their frequency, with the threshold set at 15%. Ancestral isolates, for which we isolated DNA from a single colony, were instead analyzed using breseq's consensus mode. For sample M02 we called variants again using snippy (v4.6.0)^44^, as breseq did not annotate the non-synonymous variant in *prfB* correctly due to the presence of a known ribosome slippage site in this gene. For each adapted sample, we applied the following data-processing steps to the GenomeDiff files: we first removed variants observed in the corresponding ancestral isolate. For the Δ*sbmA* samples, we further identified the variants present in all ten replicates and removed them from each sample; this was necessary because Keio knockout mutants are known to have accumulated variants since their first generation. In order to match genetic variants across strains and species and identify frequently mutated genes, we annotated the reference genomes using eggnog-mapper (v2.1.12)^45^, and used the orthologous group identifier to match ortholog genes.

### Reporting summary

Further information on research design is available in the Nature Portfolio Reporting Summary linked to this article.

## Supplementary information

Supplementary Information

Description of Additional Supplementary Files

Supplementary Data 1

Supplementary Data 2

Supplementary Data 3

Supplementary Data 4

Supplementary Data 5

Reporting Summary

Transparent Peer Review file

## Source data

Source Data

## Data Availability

Raw sequencing reads have been deposited at the European Nucleotide Archive (ENA), with accession number PRJEB81806. Source data are provided with this paper.

## Code availability

All the scripts used to process the OD readings, MIC values, the outputs of breseq, and generate all figures presented in the manuscript are available as a git repository: https://github.com/microbial-pangenomes-lab/2024_aso_evolution. The version of the code used to generate all the results presented here has been deposited in Zenodo^46^. The code is based on python scripts and Jupyter notebooks, using the following libraries: numpy (v2.0.0)^47^, scipy (v1.14.0)^48^, pandas (v2.2.2)^49^, statsmodels (v0.14.2), matplotlib (v3.9.1)^50^, seaborn (v0.13.2)^51^, and jupyter-lab (v4.2.4)^52^.
