# Supplementary material for: A systematic identification of resistance determinants to antisense antibiotics suggests adaptation strategies dependent on the delivery peptide
Source: Nat Commun. 2026 Aug 12;17:8262. doi: 10.1038/s41467-026-76357-y (PMC13469510; doi:10.1038/s41467-026-76357-y)
Supplement: Supplementary file 1 [file 41467_2026_76357_MOESM1_ESM.pdf]

Supplementary information

Supplementary data

Supplementary Data 1.
Description: Minimum inhibitory concentration (MIC) values for ASO formulations for each ancestral strain and adapted isolates.

Supplementary Data 2.
Description: List of genetic variants identified in each sample adapted to the ASO formulations, after filtering.

Supplementary Data 3.
Description: List of genetic variants identified in each sample adapted to the scrambled ASO formulations, after filtering.

Supplementary Data 4.
Description: List of asobiotics and their target sequences across the bacterial species tested in this study.

Supplementary Data 5.
Description: List of strains and primers used for the validation of selected mutants.

Supplementary figures

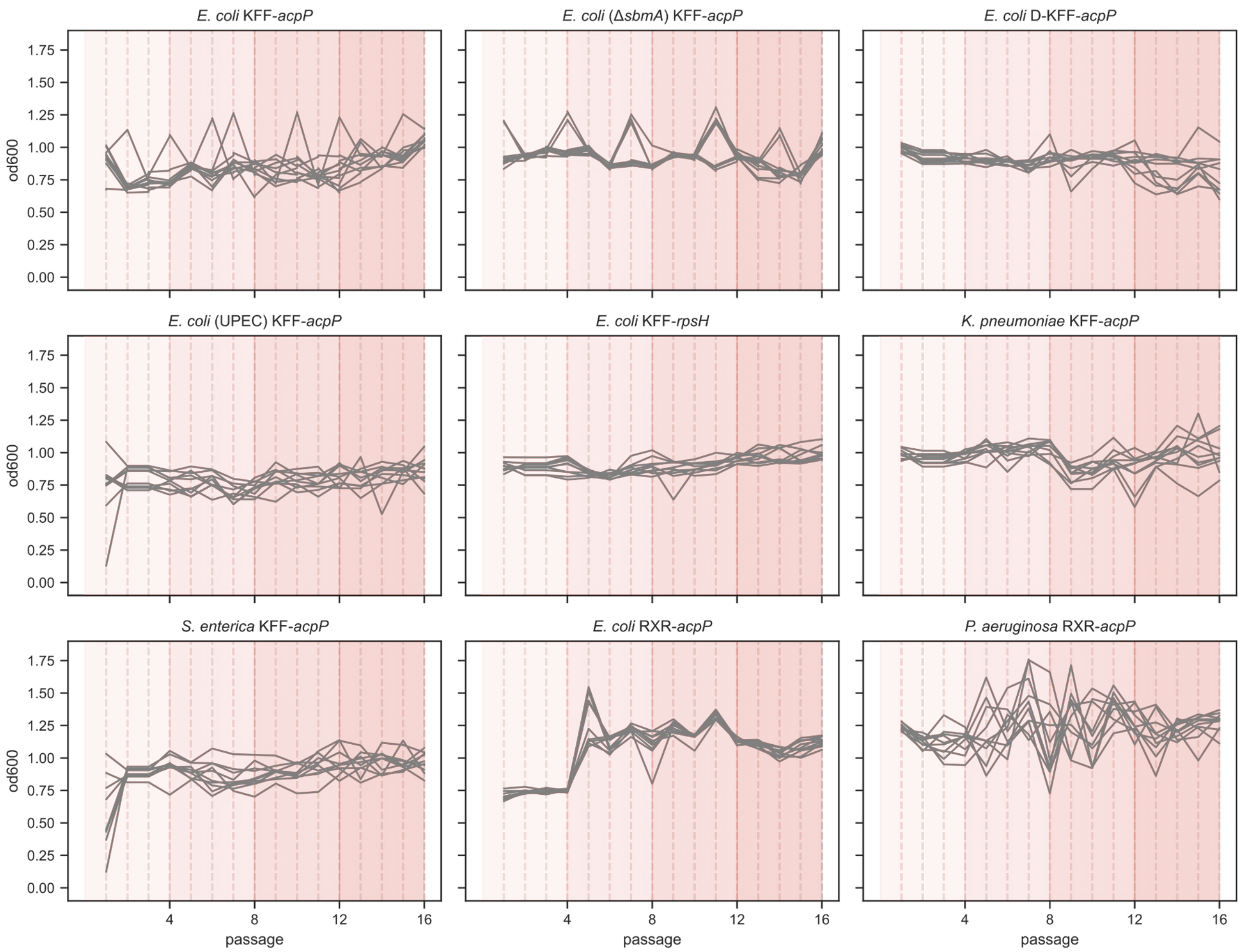

Supplementary Figure 1. Daily optical density (OD) readings from the 9 drug selection ramps. Each line indicates the OD readings of one of the 10 biological replicates.

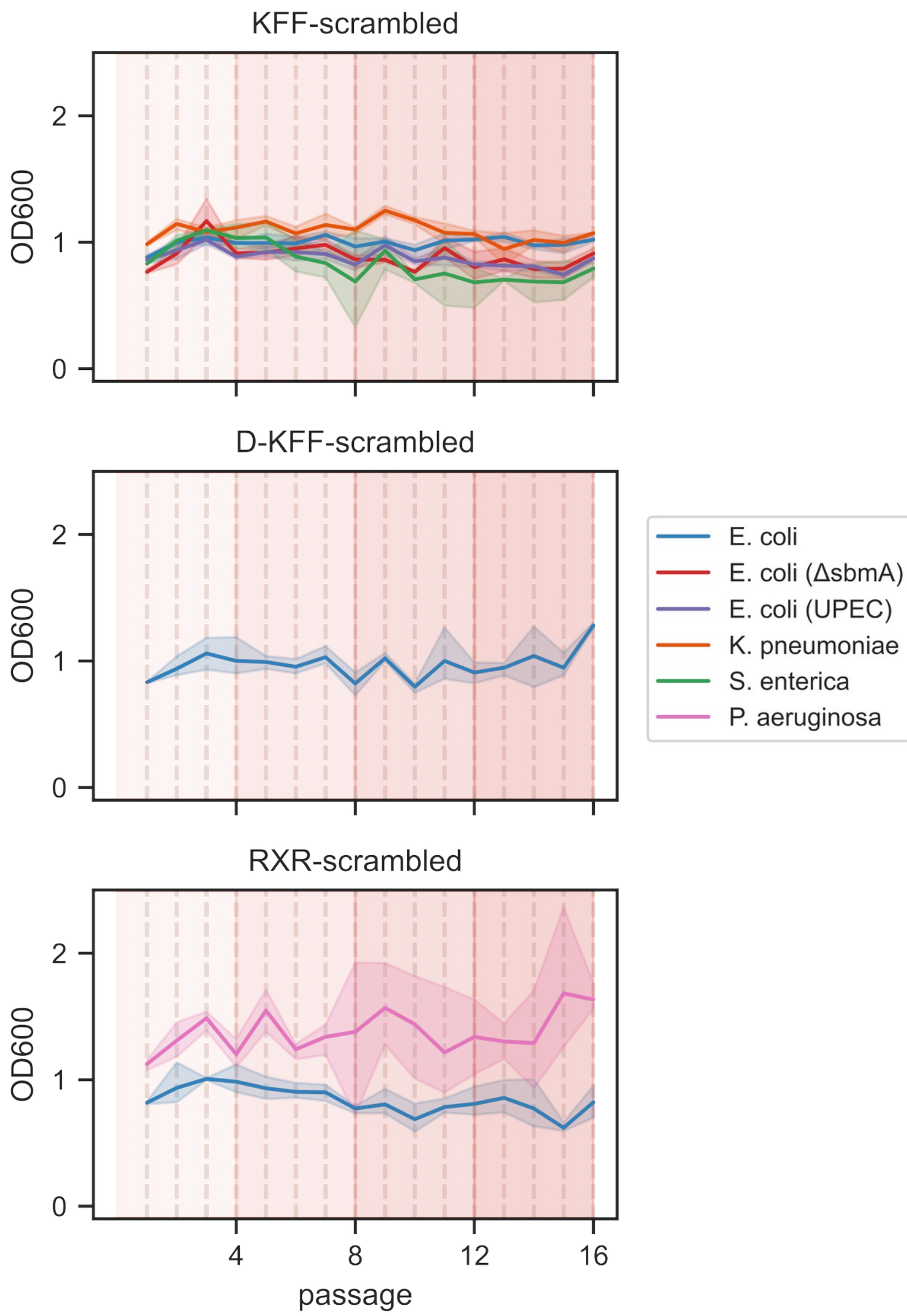

Supplementary Figure 2. Daily optical density (OD) measurements from the 8 scrambled drug selection ramps. The solid line indicates the average OD over the 3 biological replicates, the shaded area the 95% confidence interval.

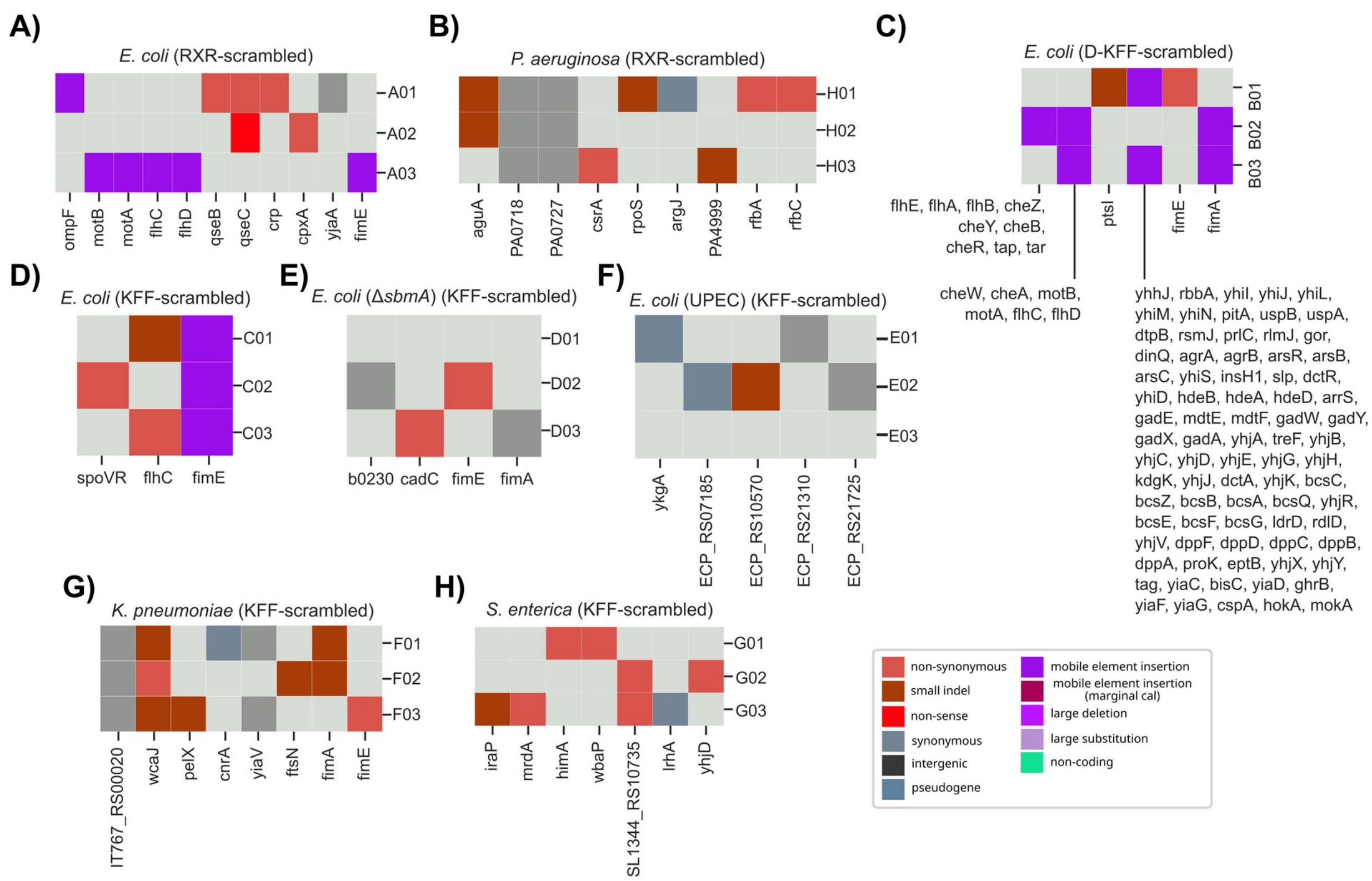

Supplementary Figure 3. Genome-wide distribution of genetic variants in strains adapted to scrambled asobiotics. Genes are ordered according to their position in the chromosome. Light gray boxes indicate that the gene does not have a genetic variant in a sample.

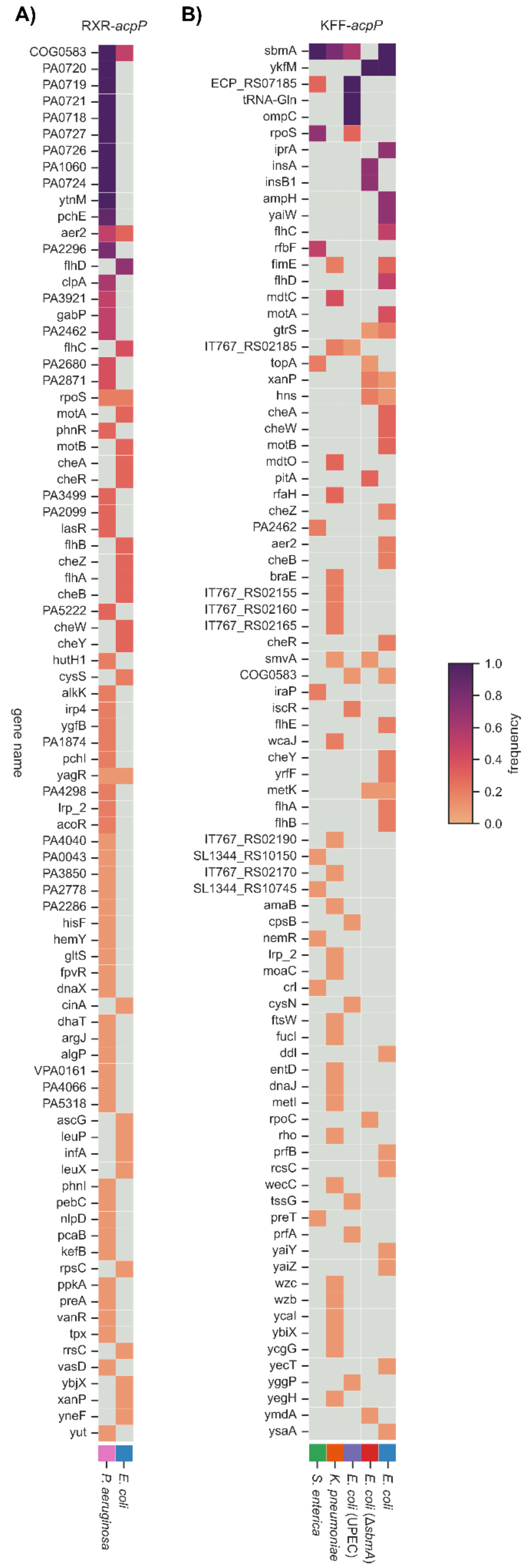

Supplementary Figure 4. Common genetic variants induced during the drug selection ramps, full list. For RXR-*acpP* (A) and KFF-*acpP* (B), all genes with at least one genetic variant in one sample are reported.

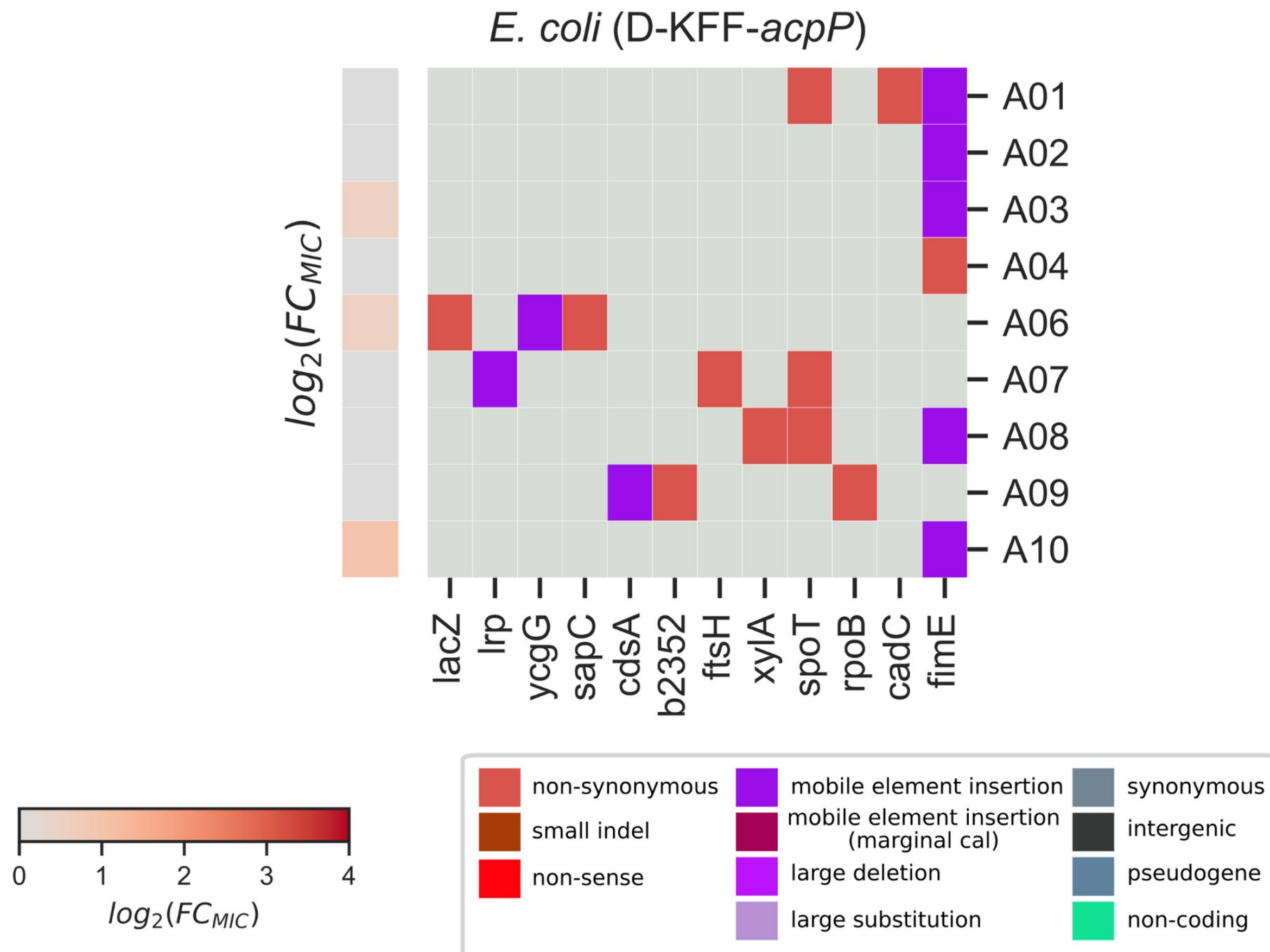

Supplementary Figure 5. Genome-wide distribution of genetic variants in *E. coli* adapted to D-KFF-*acpP*. Genes are ordered according to their position in the chromosome. Light gray boxes indicate that the gene does not have a genetic variant in a sample. The MIC fold-change with respect to the ancestral isolate is shown in gray-to-red heatmaps on the left.

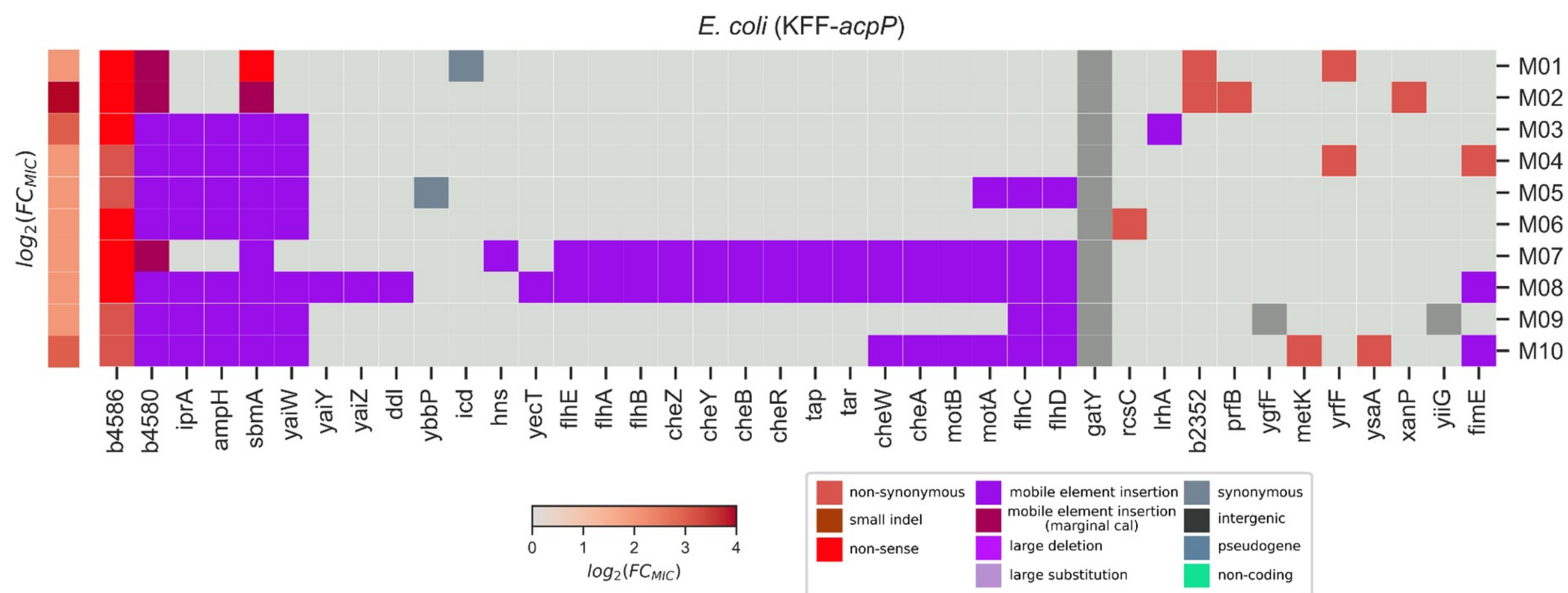

Supplementary Figure 6. Genome-wide distribution of genetic variants in *E. coli* adapted to KFF-*acpP*. Genes are ordered according to their position in the chromosome. Light gray boxes indicate that the gene does not have a genetic variant in a sample. The MIC fold-change with respect to the ancestral isolate is shown in gray-to-red heatmaps on the left.

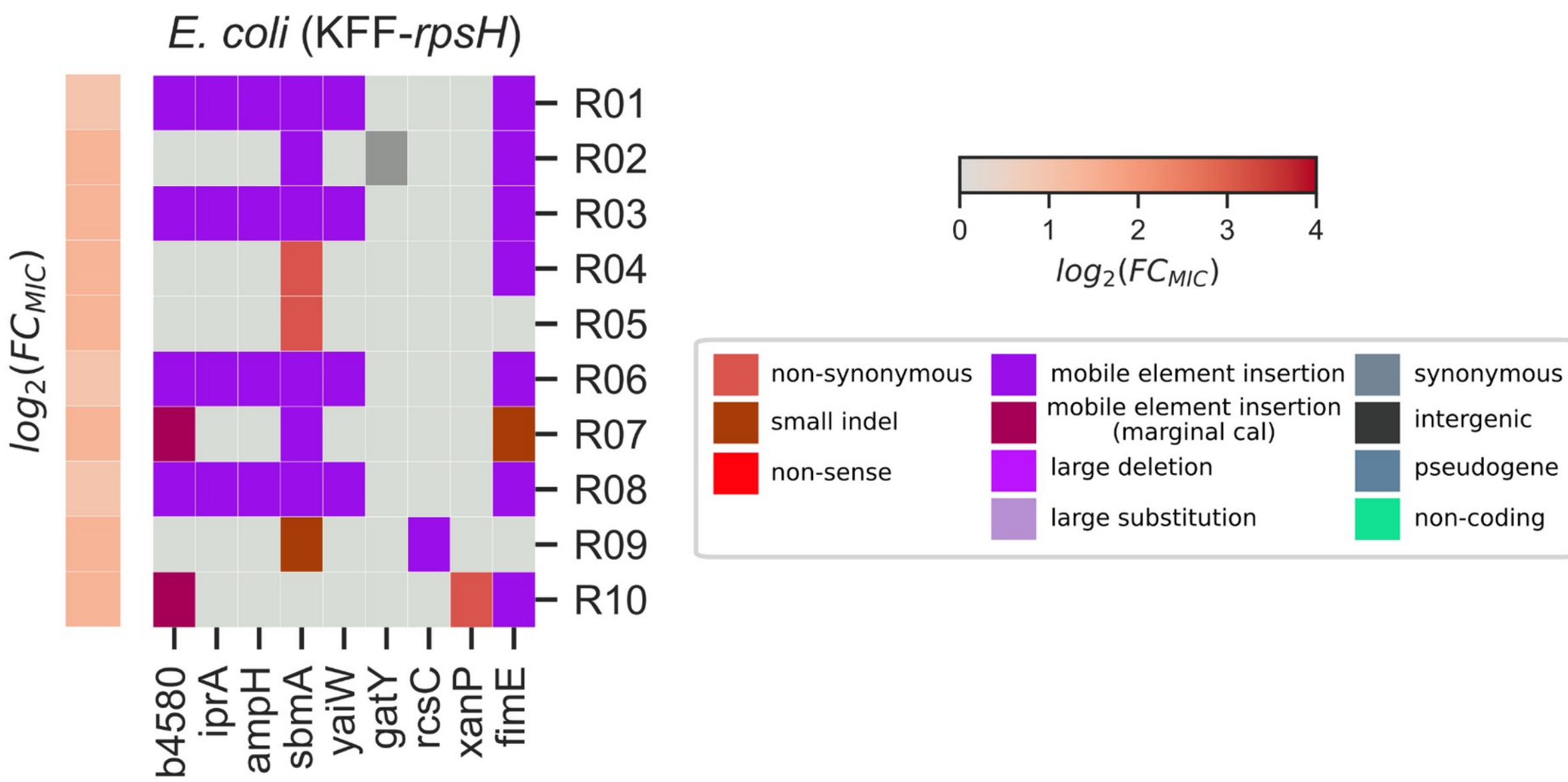

Supplementary Figure 7. Genome-wide distribution of genetic variants in *E. coli* adapted to KFF-*rpsH*. Genes are ordered according to their position in the chromosome. Light gray boxes indicate that the gene does not have a genetic variant in a sample. The MIC fold-change with respect to the ancestral isolate is shown in gray-to-red heatmaps on the left.

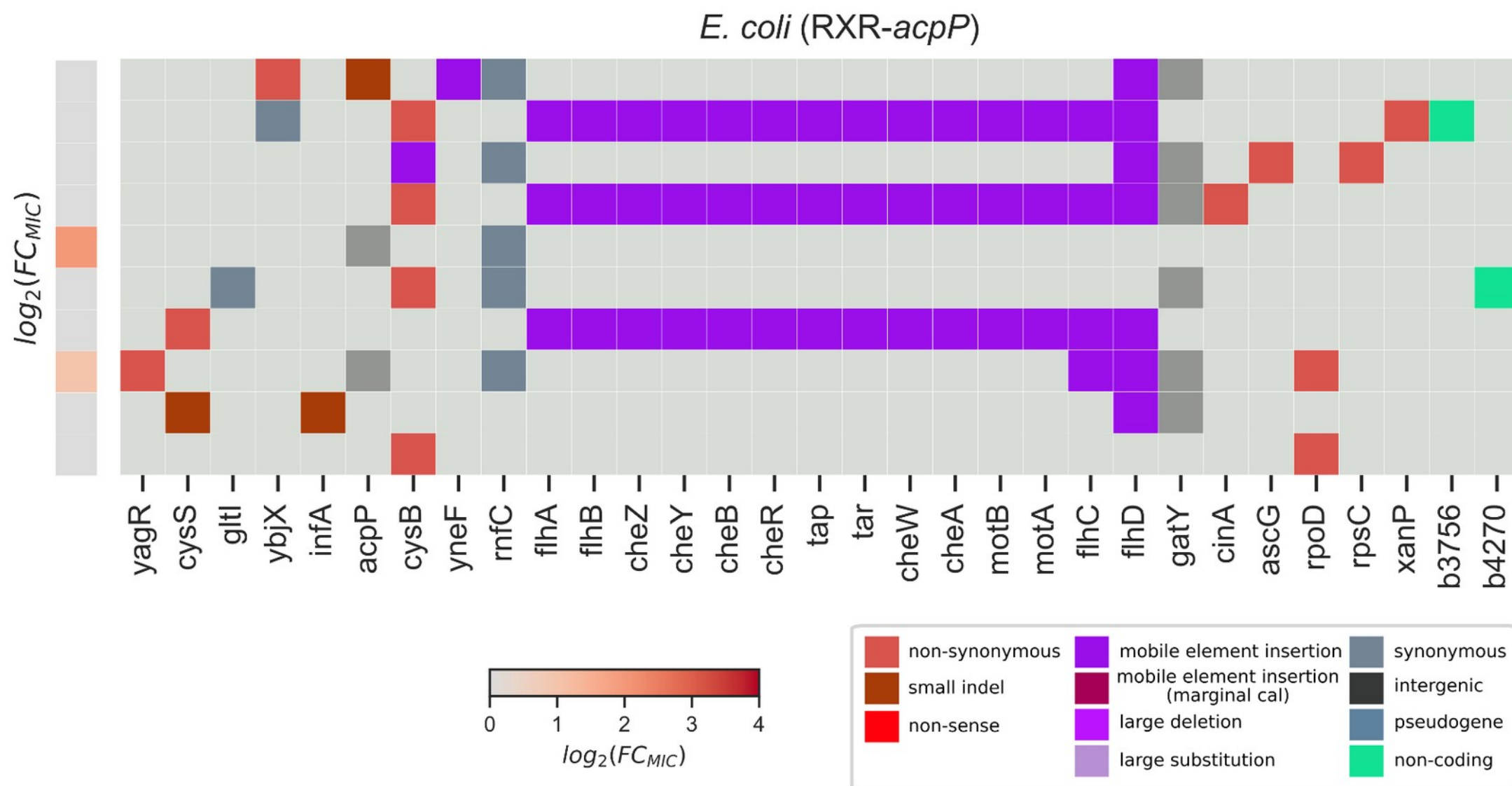

Supplementary Figure 8. Genome-wide distribution of genetic variants in *E. coli* adapted to RXR-*acpP*. Genes are ordered according to their position in the chromosome. Light gray boxes indicate that the gene does not have a genetic variant in a sample. The MIC fold-change with respect to the ancestral isolate is shown in gray-to-red heatmaps on the left.

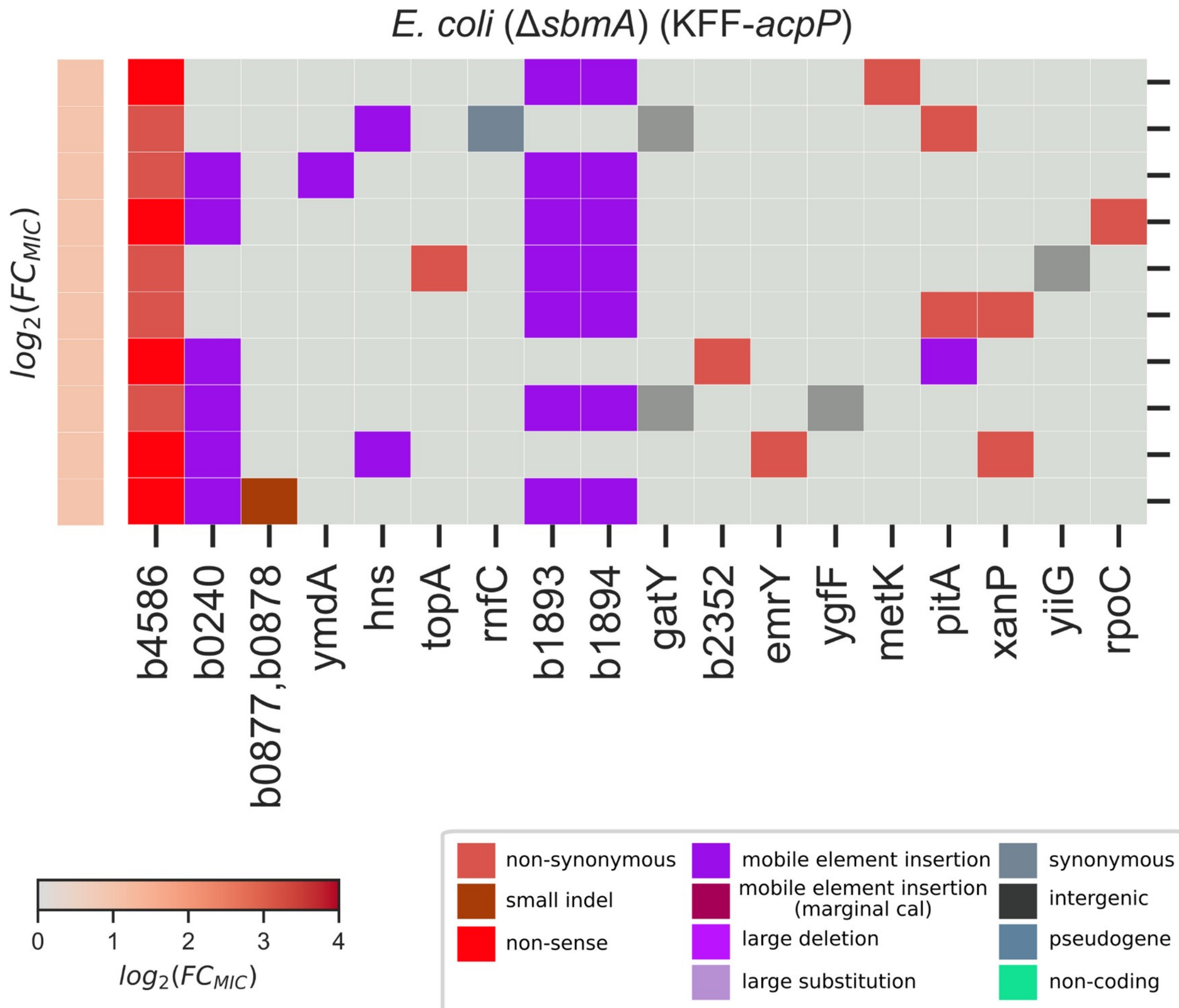

Supplementary Figure 9. Genome-wide distribution of genetic variants in the *E. coli sbmA* knockout strain adapted to KFF-*acpP*. Genes are ordered according to their position in the chromosome. Light gray boxes indicate that the gene does not have a genetic variant in a sample. The MIC fold-change with respect to the ancestral isolate is shown in gray-to-red heatmaps on the left.

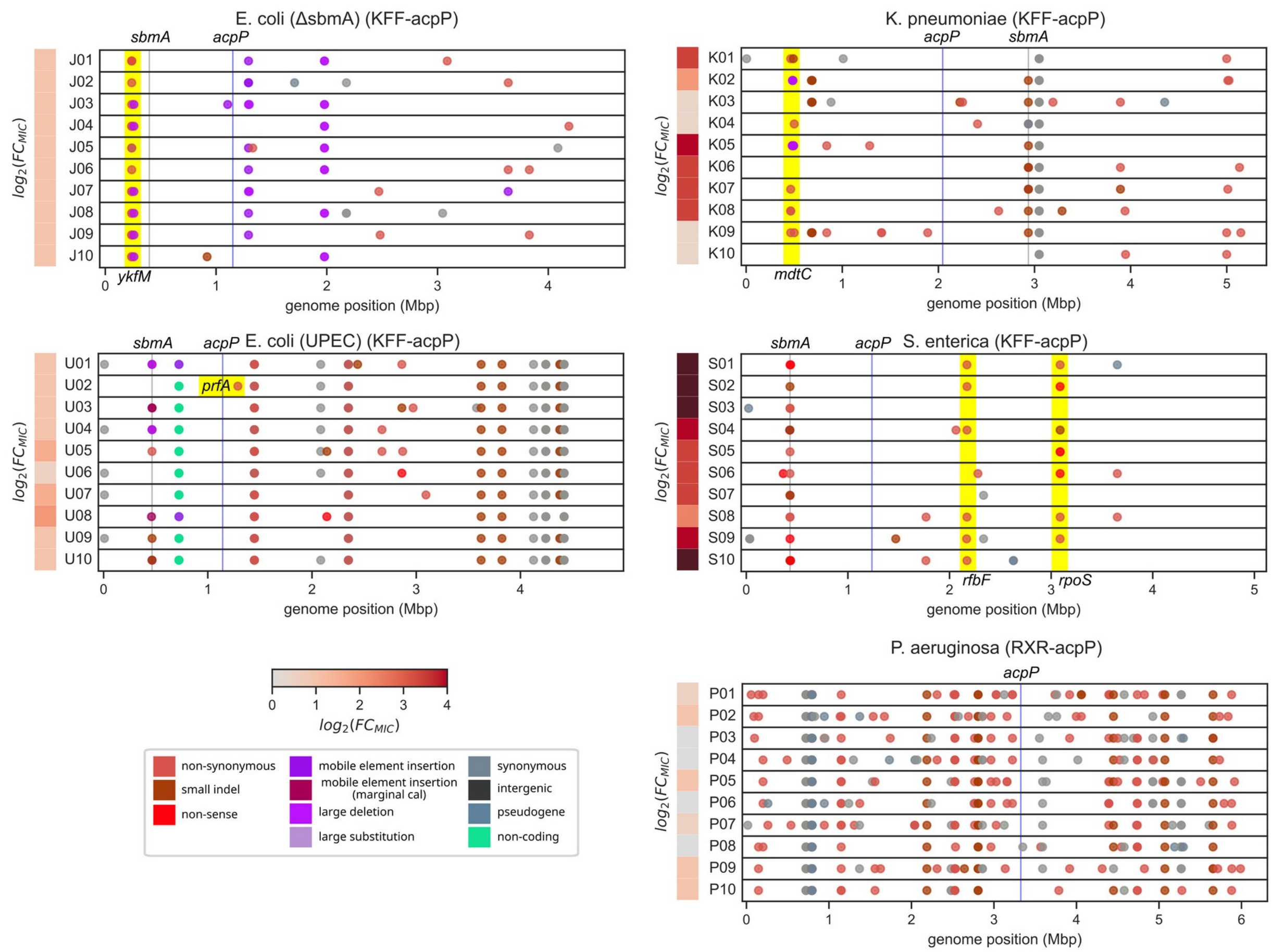

Supplementary Figure 10. Genome-wide distribution of genetic variants in each biological replicate for those species/ASO formulations not shown in Figure 3. The MIC fold-change with respect to the ancestral isolate is shown in gray-to-red heatmaps on the left. The gray and blue vertical lines indicate the location of the *sbmA* and the PNA target gene (*acpP*), respectively.

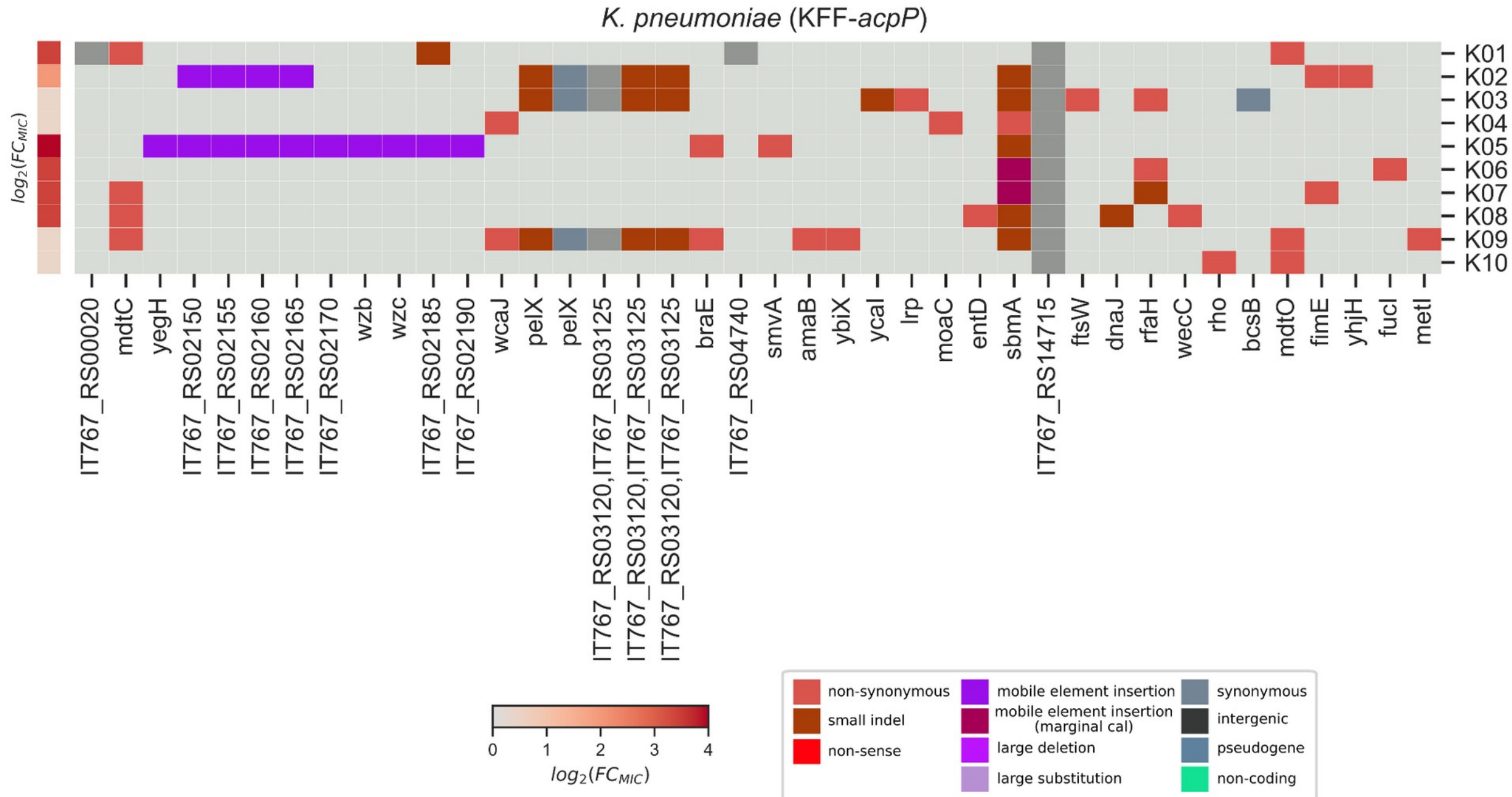

Supplementary Figure 11. Genome-wide distribution of genetic variants in *K. pneumoniae* adapted to KFF-*acpP*. Genes are ordered according to their position in the chromosome. Light gray boxes indicate that the gene does not have a genetic variant in a sample. The MIC fold-change with respect to the ancestral isolate is shown in gray-to-red heatmaps on the left.

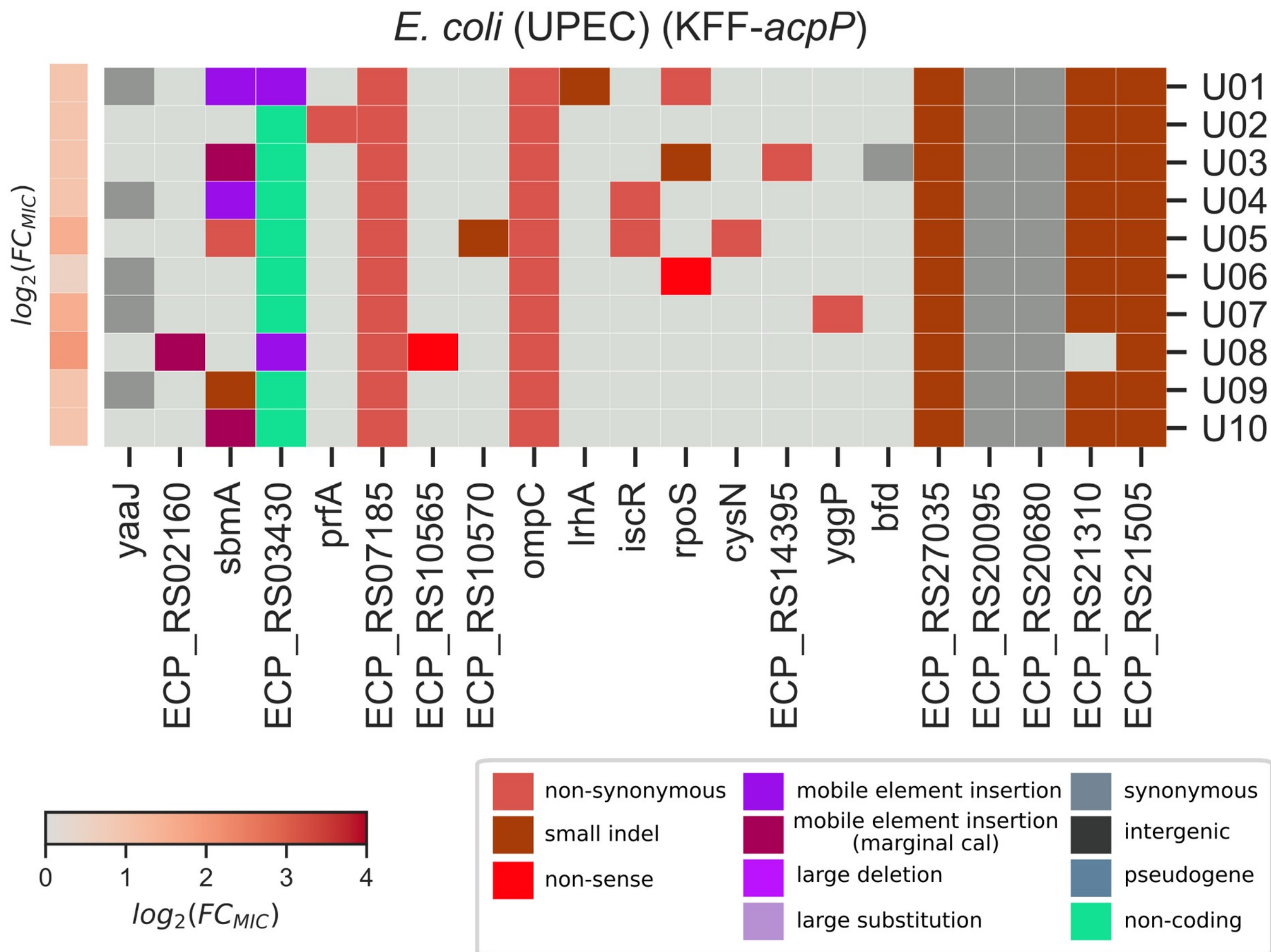

Supplementary Figure 12. Genome-wide distribution of genetic variants in *E. coli* UPEC adapted to KFF-*acpP*. Genes are ordered according to their position in the chromosome. Light gray boxes indicate that the gene does not have a genetic variant in a sample. The MIC fold-change with respect to the ancestral isolate is shown in gray-to-red heatmaps on the left.

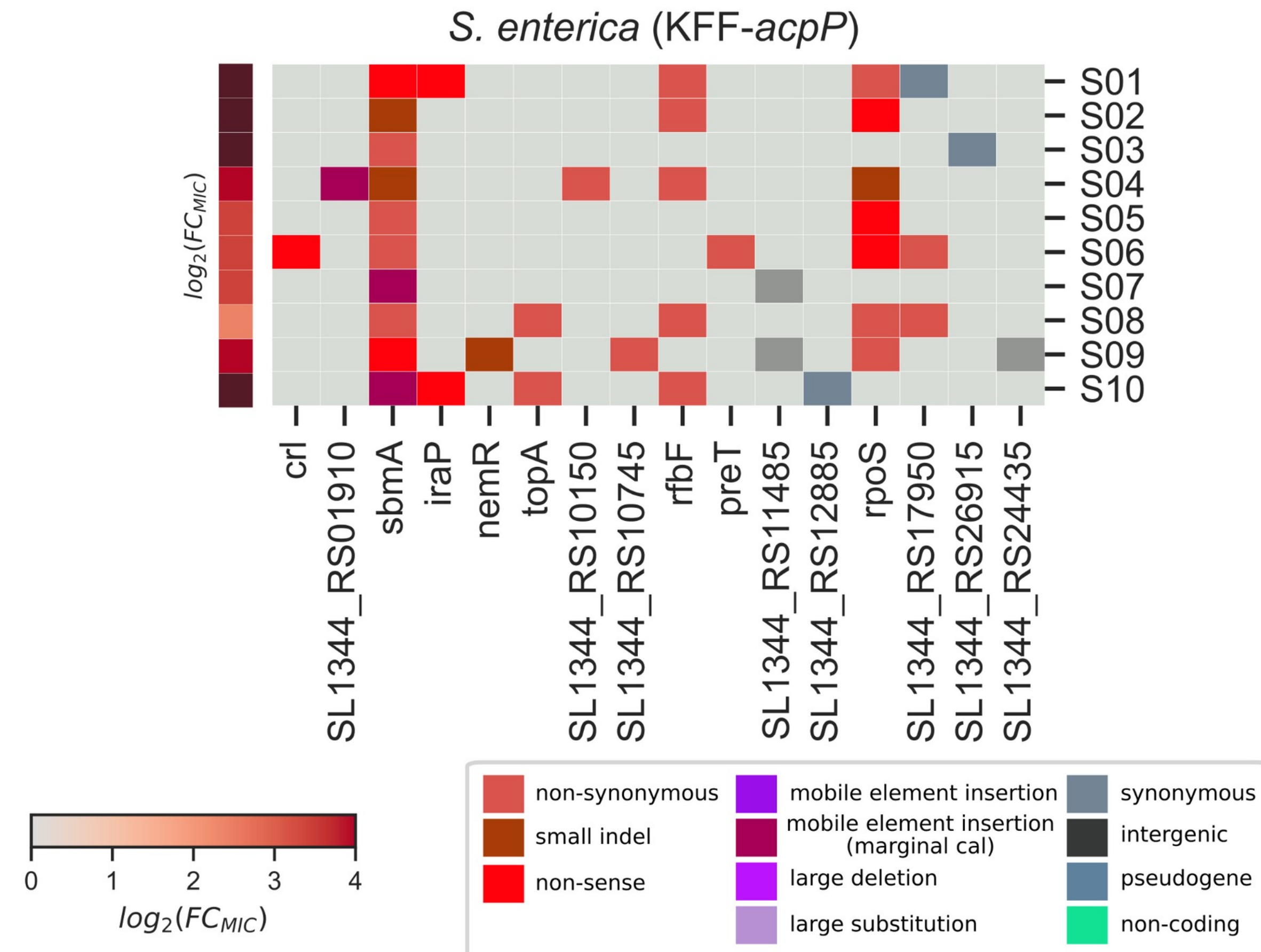

Supplementary Figure 13. Genome-wide distribution of genetic variants in *S. enterica* adapted to KFF-*acpP*. Genes are ordered according to their position in the chromosome. Light gray boxes indicate that the gene does not have a genetic variant in a sample. The MIC fold-change with respect to the ancestral isolate is shown in gray-to-red heatmaps on the left.

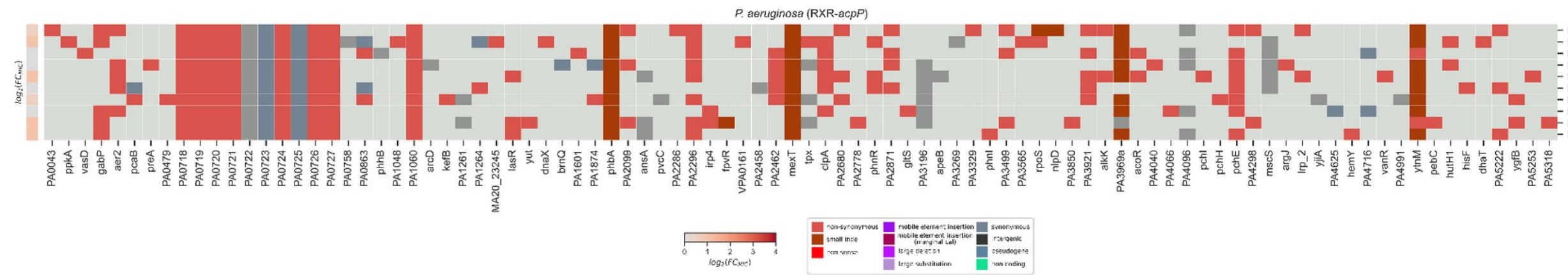

Supplementary Figure 14. Genome-wide distribution of genetic variants in *P. aeruginosa* adapted to RXR-*acpP*. Genes are ordered according to their position in the chromosome. Light gray boxes indicate that the gene does not have a genetic variant in a sample. The MIC fold-change with respect to the ancestral isolate is shown in gray-to-red heatmaps on the left.

Supplementary Notes

Description: HPLC and MS spectra for ASO formulations used in this study (following pages).

HZIpna-1
KFF-acpP (1st batch)

Attachment for a Soluble Peptide in the Platform Peptide Synthesis

(please fill out the yellow fields and send together with the Excel order form by email to peptide@helmholtz-hzi.de)

contact person:	**Linda Popella, HIRI**	peptide name given by platform:	**23Linda-1a**
phone & email:		your name of peptide:	**KFF-1-acpP**
HZI unit:		date of placement:	**27.01.2023**

sequence: **K-F-F-K-F-F-K-F-F-K---c-t-c-a-t-a-c-t-c-t-amide**

(if labelled with *: difficult coupling according to prediction by computer program; will be added by platform)

N-terminus ☒ free ☐ acetylated ☐ biotinylated ☐ others:

C-terminus ☐ free acid ☒ amide ☐ others:

length of peptide **20** mol. weight (average neutral mass): **4032.31** (+ **11** x 114) desired amount: **10** mg

intended usage: **antimicrobial activity**

HPLC-purificat. ☒ required ☐ not necessary ☐ according to agreement

☐ exchange of counter ion TFA → HCl requested (with surcharge)

☐ repetition synthesis; our previous name:

comments: **Peptidsynthese auf vorsynthetisiertem PNA (23Linda-1_4_9-PNA), alles PNA-Zyklen**

General remarks: We carry out the syntheses on standard scales of 50 micromoles. Product yields vary depending on peptide length and purity. If larger amounts are needed please get in touch with us. Please restrict the length of the peptide to a maximum of 30 amino acids. Slightly longer peptides may be possible upon request. Modifications like phosphorylation, biotinylation, fluorescence labelling and others are available upon request and optional surcharge. All peptides are delivered with analytical HPLC and mass analysis. Allow a production time of 2-4 weeks, depending on our order situation and the desired quality. Remark concerning mass analysis by MALDI: In addition to the molecular ion $[M+H]^+$ some peptides show the sodium salt $[M+23]^+$ (possibly as the major signal) and the potassium salt $[M+39]^+$. This is no criterion for the quality of the product. Remark concerning mass analysis by HPLC-ESI: Multiple charged mass ions are usually dominating and are no indication of decomposition of the product.

Synthesedatum:		Ansatz: **10** µmol		
Gerät:	☐ Liberty Blue	☐ Syro	☐ manuell	
Besonderheiten während der Synthese:				
Abspaltung	☐ Silan	☐ KW	☐ KCys	Dauer:
gewün. Peptid:	☐ Hauptprodukt	☐ vorhanden		
	☐ nicht detektierbar	☐ Wiederholungssynthese erforderlich		
Anzahl präparativer HPLCs:		Anzahl analytischer HPLCs:		
Ausbeute Rohpeptid:	mg	Ausbeute gereinigtes Peptid:	mg	
Datum der Fertigstellung:		☐ Preisaufschlag erforderlich, Grund:		

Auftraggeber benachrichtigt Datum per ☐ Telefon ☐ Email

☐ Peptid übergeben Datum

Empfänger:

23Linda-1a	KFF-1-acpP	6.7 mg
K-F-F-K-F-F-K-F-F-K---c-t-c-a-t-a-c-t-c-t-amide		
☐ crude ☒ HPLC-purif.	MW 4032.3 (+ 11 x 114)	Date 20.2.23
At free N-terminus and every Arg, His & Lys peptide has TFA (MW 114) as counter ion; aqueous solutions are strongly acidic		

HZIpna-1
KFF-acpP (1st batch)

Analytical data of 23Linda-1a = KFF-1-acpP

Analytical HPLC of **purified peptide** (Agilent 1100 series; column Aeris Peptide C18, 3.6 µm, 100 Å, 2.1x50 mm (Phenomenex); gradient 5% to 100% ACN (0.1% trifluoroacetic acid) in 20 min, 0.7 ml/min, detection at 220 and 280 nm).

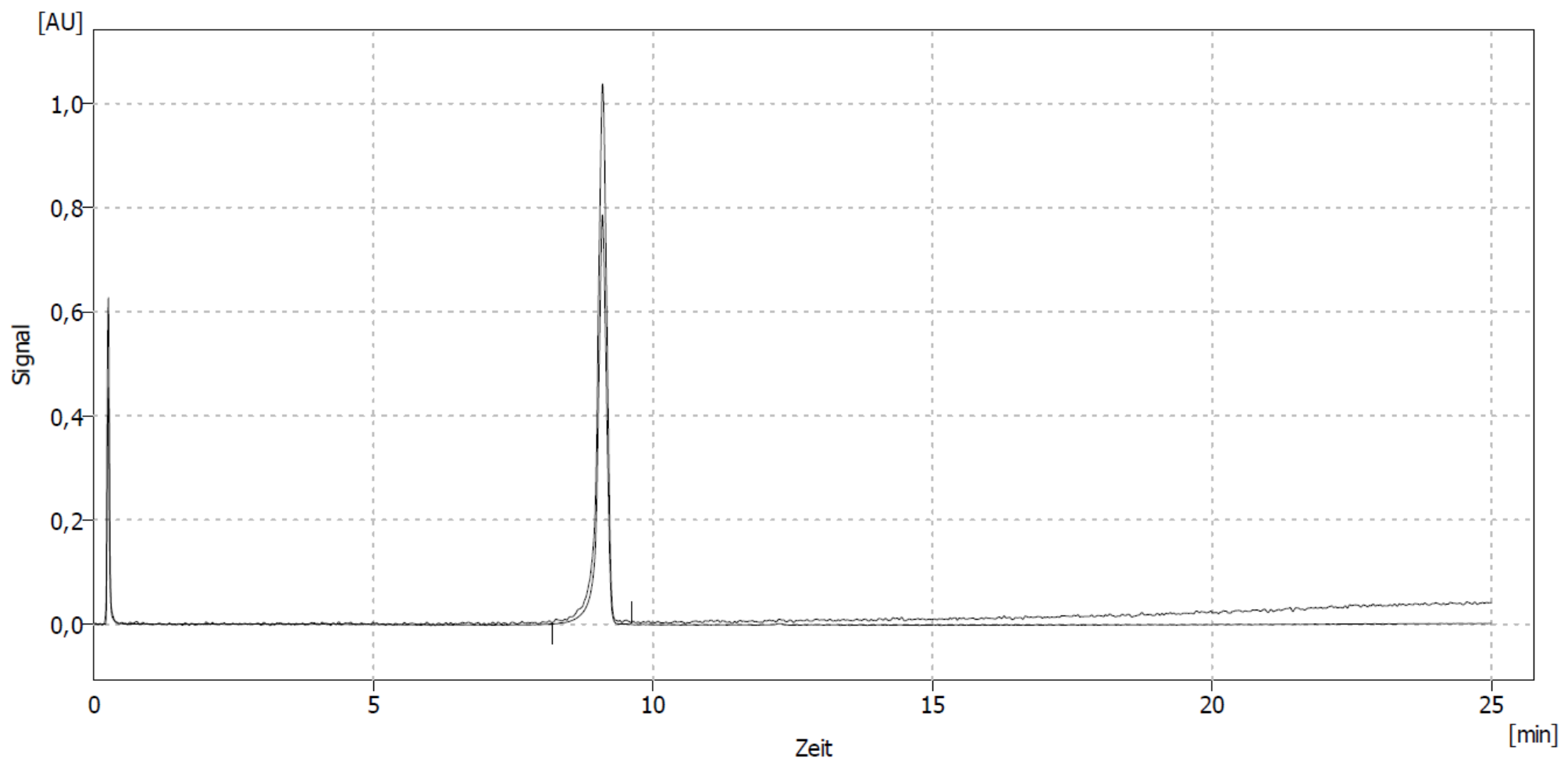

LC-ESI-MS (Bruker Amazon); HPLC Agilent 1200 series, column Gemini-NX C18, 3 µm, 110 Å, 2.0x50 mm (Phenomenex); gradient 5-95% ACN (0.1% formic acid) in 19 min, 0.4 ml/min, detection at 220 nm.
Expected: 4032.31 (average neutral mass)

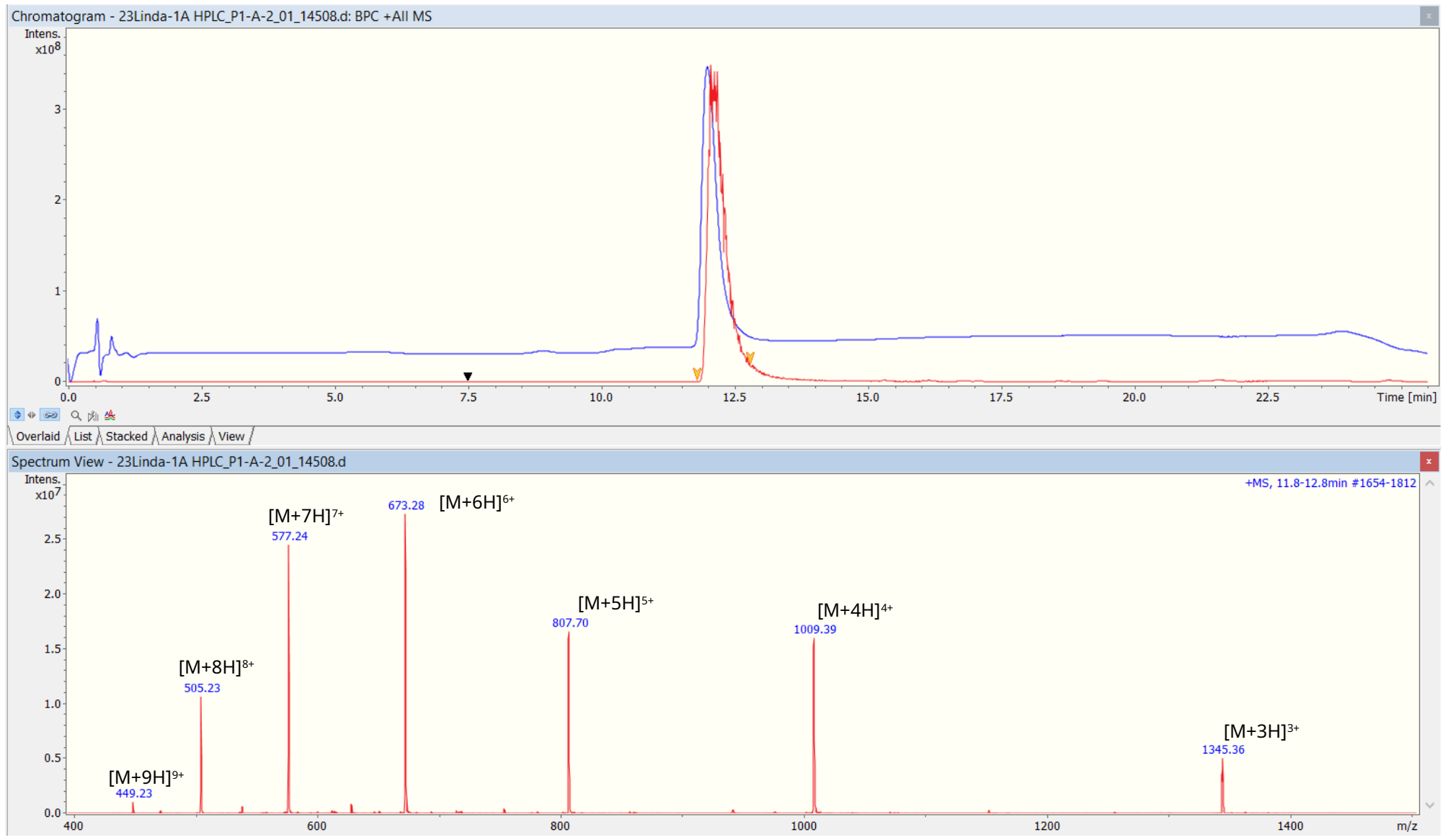

HZIpna-12
KFF-acpP (2nd batch)

Attachment for a Soluble Peptide in the Platform Peptide Synthesis

(please fill out the yellow fields and send together with the Excel order form by email to peptide@helmholtz-hzi.de)

contact person:	**Linda Popella, HIRI**	peptide name given by platform:	**23Linda-12**
phone & email:		your name of peptide:	**KFF-~~rpsH~~ = acpP**
HZI unit:		date of placement:	**05.04.2023**

Conjugate was labeled wrongly (correct: acpP)

sequence: **K-F-F-K-F-F-K-F-F-K---c-t-c-a-t-a-c-t-c-t-amide**

(if labelled with *: difficult coupling according to prediction by computer program; will be added by platform)

N-terminus: ☒ free ☐ acetylated ☐ biotinylated ☐ others:

C-terminus: ☐ free acid ☒ amide ☐ others:

length of peptide **20** mol. weight (average neutral mass): **4033.28** (+ **11** x 114) desired amount: **10** mg

intended usage: **antimicrobial activity**

HPLC-purificat. ☒ required ☐ not necessary ☐ according to agreement

☐ exchange of counter ion TFA → HCl requested (with surcharge)

☐ repetition synthesis; our previous name:

comments:

General remarks: We carry out the syntheses on standard scales of 50 micromoles. Product yields vary depending on peptide length and purity. If larger amounts are needed please get in touch with us. Please restrict the length of the peptide to a maximum of 30 amino acids. Slightly longer peptides may be possible upon request. Modifications like phosphorylation, biotinylation, fluorescence labelling and others are available upon request and optional surcharge. All peptides are delivered with analytical HPLC and mass analysis. Allow a production time of 2-4 weeks, depending on our order situation and the desired quality. Remark concerning mass analysis by MALDI: In addition to the molecular ion $[M+H]^+$ some peptides show the sodium salt $[M+23]^+$ (possibly as the major signal) and the potassium salt $[M+39]^+$. This is no criterion for the quality of the product. Remark concerning mass analysis by HPLC-ESI: Multiple charged mass ions are usually dominating and are no indication of decomposition of the product.

Synthesedatum:		Ansatz: **10** µmol		
Gerät:	☐ Liberty Blue	☐ Syro	☐ manuell	
Besonderheiten während der Synthese:				
Abspaltung	☐ Silan	☐ KW	☐ KCys	Dauer:
gewün. Peptid:	☐ Hauptprodukt	☐ vorhanden		
	☐ nicht detektierbar	☐ Wiederholungssynthese erforderlich		
Anzahl präparativer HPLCs:		Anzahl analytischer HPLCs:		
Ausbeute Rohpeptid:	mg	Ausbeute gereinigtes Peptid:	mg	
Datum der Fertigstellung:		☐ Preisaufschlag erforderlich, Grund:		

Auftraggeber benachrichtigt Datum per ☐ Telefon ☐ Email

☐ Peptid übergeben Datum

Empfänger:

**23Linda-12**	**KFF-~~rpsH~~** acpP	**1.2 mg**
**K-F-F-K-F-F-K-F-F-K---c-t-c-a-t-a-c-t-c-t-amide**		
☐ crude ☒ HPLC-purif.	**MW 4033.3** (+ 11 x 114)	**Date 2.5.23**
At free N-terminus and every Arg, His & Lys peptide has TFA (MW 114) as counter ion; aqueous solutions are strongly acidic		

HZIpna-12
KFF-acpP (2nd batch)

Analytical data of 23Linda-12 = KFF-acpP

Analytical HPLC of **purified peptide** (Agilent 1100 series; column Aeris Peptide C18, 3.6 µm, 100 Å, 2.1x50 mm (Phenomenex); gradient 5% to 100% ACN (0.1% trifluoroacetic acid) in 20 min, 0.7 ml/min, detection at 220 and 280 nm).

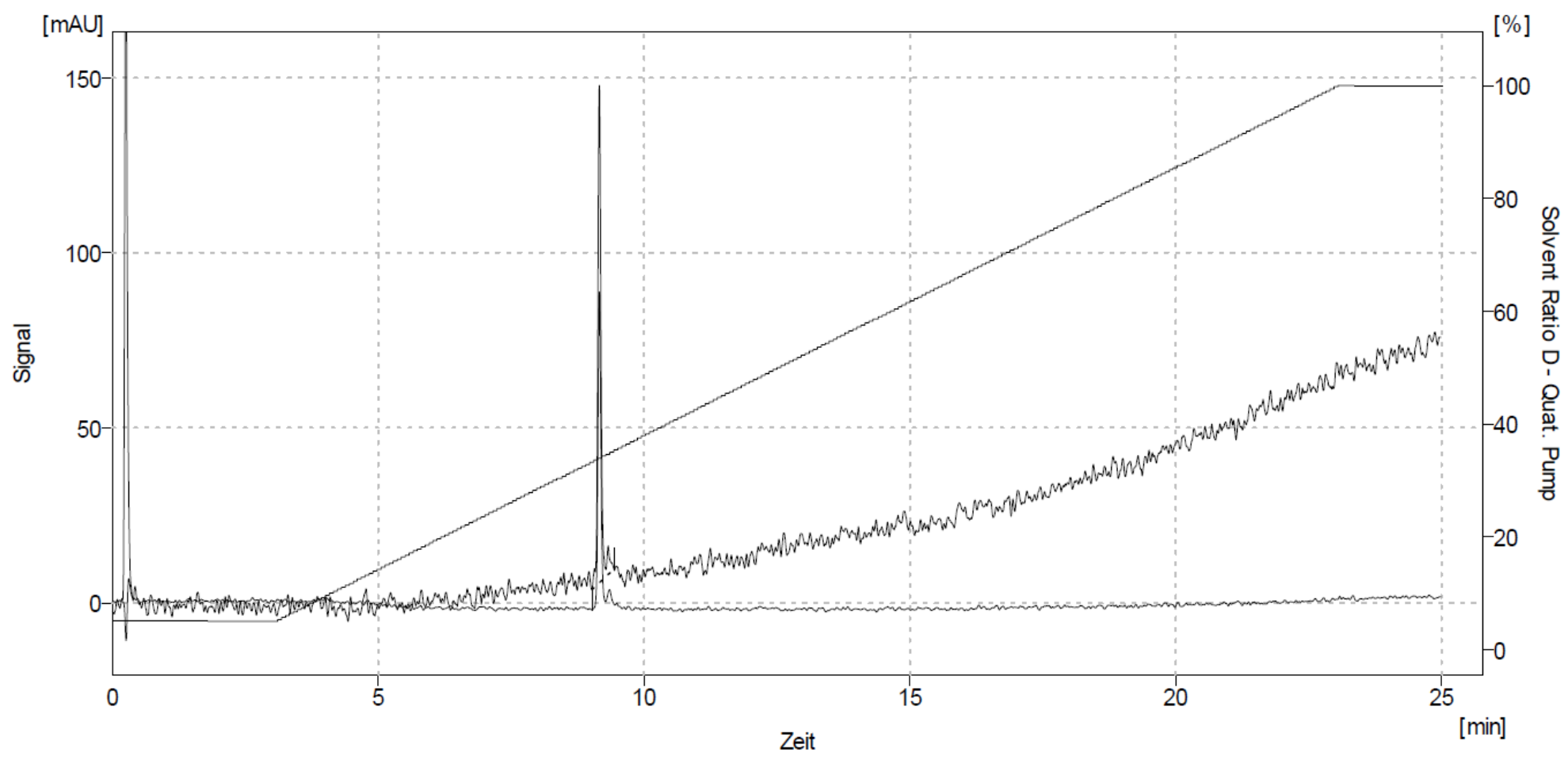

LC-ESI-MS (Bruker Amazon); HPLC Agilent 1200 series, column Gemini-NX C18, 3 µm, 110 Å, 2.0x50 mm (Phenomenex); gradient 5-95% ACN (0.1% formic acid) in 19 min, 0.4 ml/min, detection at 220 nm.
Expected: 4033.28 (average neutral mass)

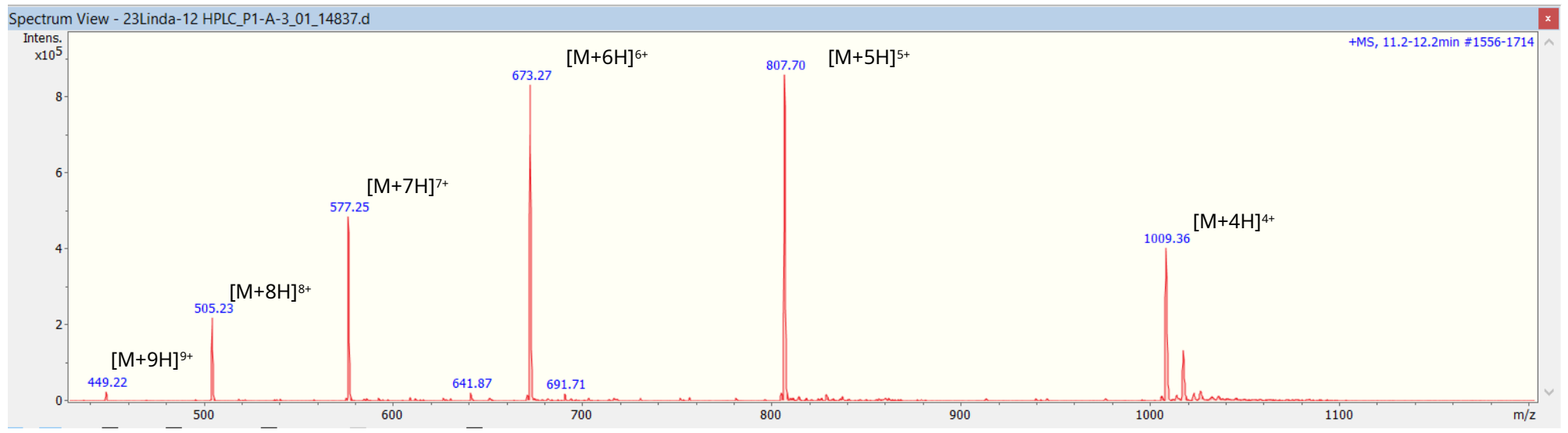

HZIpna-4
RXR-acpP (E. coli)

Attachment for a Soluble Peptide in the Platform Peptide Synthesis

(please fill out the yellow fields and send together with the Excel order form by email to peptide@helmholtz-hzi.de)

contact person:	**Linda Popella, HIRI**	peptide name given by platform:	**23Linda-4a**
phone & email:		your name of peptide:	**RXR-acpP**
HZI unit:		date of placement:	**27.01.2023**

sequence: **R-(Ahx)-R-R-(Ahx)-R-R-(Ahx)-R-R-(Ahx)-R-(Ahx)-ßAla---c-t-c-a-t-a-c-t-c-t-amide**

(if labelled with *: difficult coupling according to prediction by computer program; will be added by platform)

N-terminus ☒ free ☐ acetylated ☐ biotinylated ☐ others:

C-terminus ☐ free acid ☒ amide ☐ others:

length of peptide **24** mol. weight (average neutral mass): **4523.93** (+ **15** x 114) desired amount: **10** mg

intended usage: **antimicrobial activity**

HPLC-purificat. ☒ required ☐ not necessary ☐ according to agreement

☐ exchange of counter ion TFA → HCl requested (with surcharge)

☐ repetition synthesis; our previous name:

comments: **Peptidsynthese auf vorsynthetisiertem PNA (23Linda-1_4_9-PNA); PNA Zyklen**

General remarks: We carry out the syntheses on standard scales of 50 micromoles. Product yields vary depending on peptide length and purity. If larger amounts are needed please get in touch with us. Please restrict the length of the peptide to a maximum of 30 amino acids. Slightly longer peptides may be possible upon request. Modifications like phosphorylation, biotinylation, fluorescence labelling and others are available upon request and optional surcharge. All peptides are delivered with analytical HPLC and mass analysis. Allow a production time of 2-4 weeks, depending on our order situation and the desired quality. Remark concerning mass analysis by MALDI: In addition to the molecular ion $[M+H]^+$ some peptides show the sodium salt $[M+23]^+$ (possibly as the major signal) and the potassium salt $[M+39]^+$. This is no criterion for the quality of the product. Remark concerning mass analysis by HPLC-ESI: Multiple charged mass ions are usually dominating and are no indication of decomposition of the product.

Synthesedatum:		Ansatz: **10** µmol		
Gerät:	☐ Liberty Blue	☐ Syro	☐ manuell	
Besonderheiten während der Synthese:				
Abspaltung	☐ Silan	☐ KW	☐ KCys	Dauer:
gewün. Peptid:	☐ Hauptprodukt	☐ vorhanden		
	☐ nicht detektierbar	☐ Wiederholungssynthese erforderlich		
Anzahl präparativer HPLCs:		Anzahl analytischer HPLCs:		
Ausbeute Rohpeptid:	mg	Ausbeute gereinigtes Peptid:	mg	
Datum der Fertigstellung:		☐ Preisaufschlag erforderlich, Grund:		

Auftraggeber benachrichtigt Datum per ☐ Telefon ☐ Email

☐ Peptid übergeben Datum

Empfänger:

**23Linda-4a**	**RXR-acpP**	**21.2 mg**
**R(Ahx)RR(Ahx)RR(Ahx)RR(Ahx)R(Ahx)-ßAla-c-t-c-a-t-a-c-t-c-t-amide**		
☐ **crude** ☒ **HPLC-purif.**	**MW 4523.9** (+ 15 x 114)	**Date**
At free N-terminus and every Arg, His & Lys peptide has TFA (MW 114) as counter ion; aqueous solutions are strongly acidic		

HZIpna-4
RXR-acpP (E. coli)

Analytical data of 23Linda-4a = RXR-acpP

Analytical HPLC of **purified peptide** (Agilent 1100 series; column Aeris Peptide C18, 3.6 µm, 100 Å, 2.1x50 mm (Phenomenex); gradient 1% to 30% ACN (0.1% trifluoroacetic acid) in 20 min, 0.7 ml/min, detection at 220 and 280 nm).

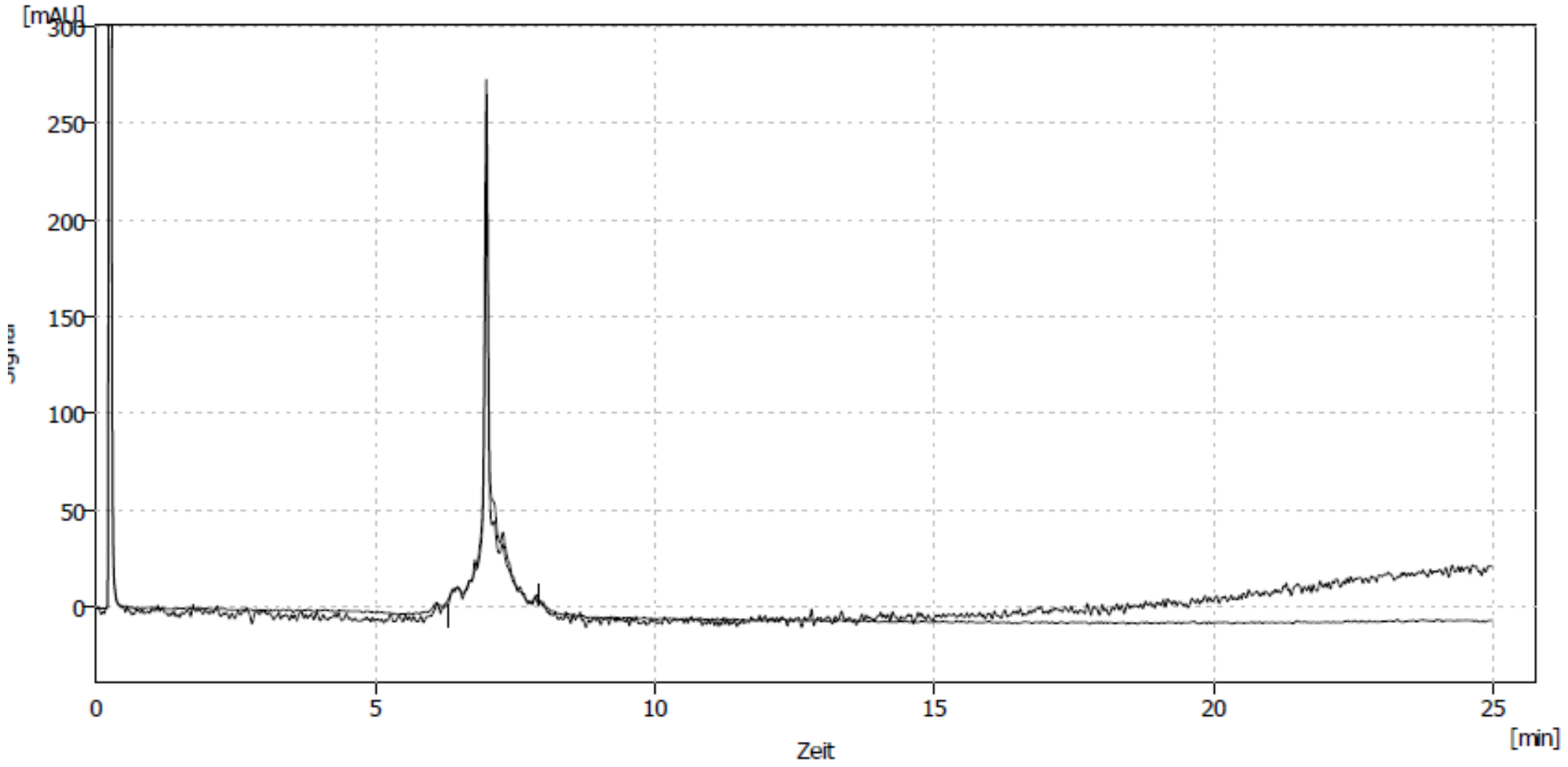

LC-ESI-MS (Bruker Amazon); HPLC Agilent 1200 series, column Gemini-NX C18, 3 µm, 110 Å, 2.0x50 mm (Phenomenex); gradient 5-95% ACN (0.1% formic acid) in 19 min, 0.4 ml/min, detection at 220 nm.
Expected: 4523.93 (average neutral mass)

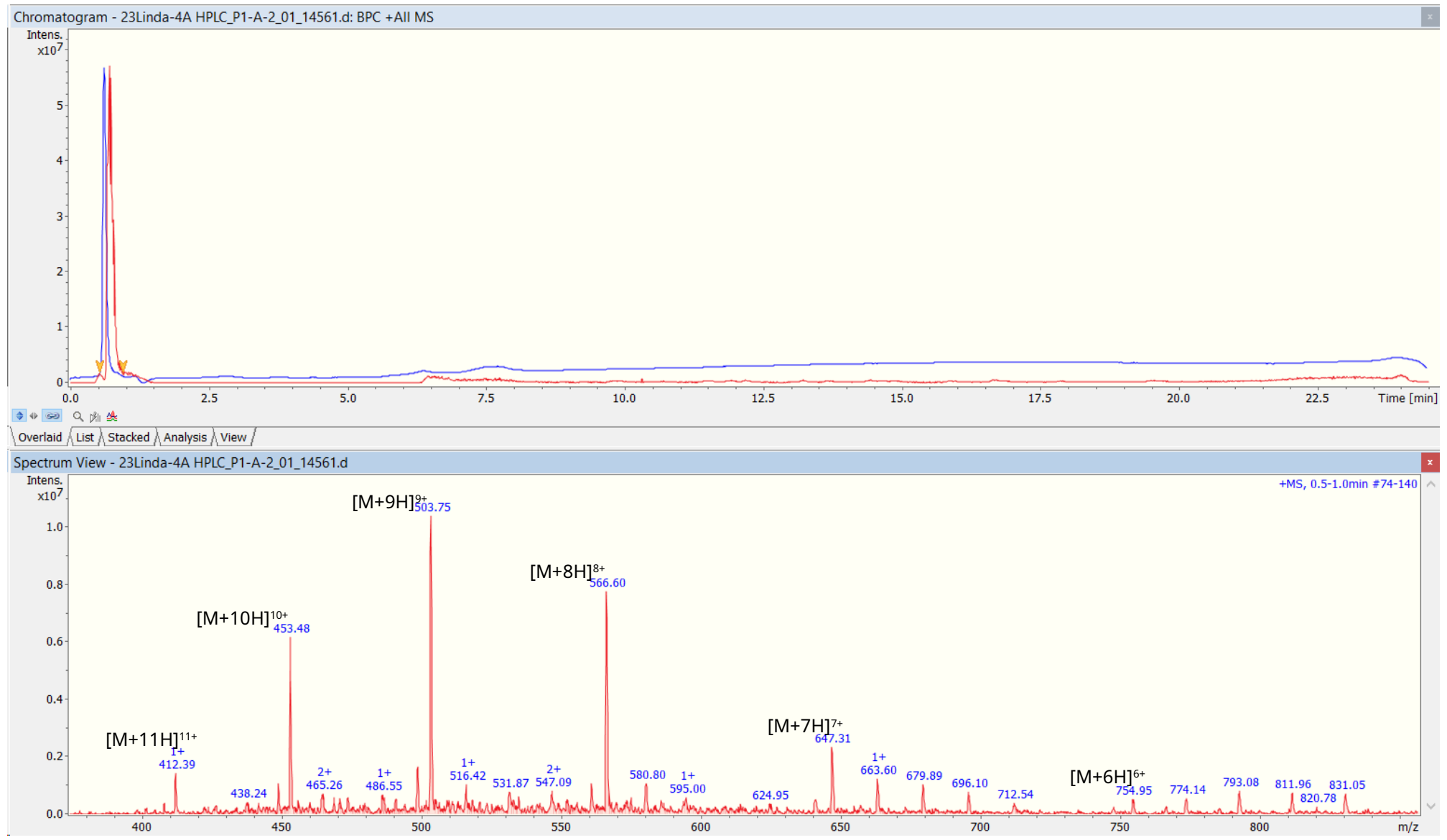

HZIpna-14
KFF-rpsH

Attachment for a Soluble Peptide in the Platform Peptide Synthesis

(please fill out the yellow fields and send together with the Excel order form by email to peptide@helmholtz-hzi.de)

contact person:	**Linda Popella, HIRI**	peptide name given by platform:	**23Linda-14**
phone & email:		your name of peptide:	**KFF-rpsH_richtig**
HZI unit:		date of placement:	**02.05.2023**

sequence: **K-F-F-K-F-F-K-F-F-K---c-t-c-a-t-c-t-g-t-c-amide**

(if labelled with *: difficult coupling according to prediction by computer program; will be added by platform)

N-terminus ☒ free ☐ acetylated ☐ biotinylated ☐ others:

C-terminus ☐ free acid ☒ amide ☐ others:

length of peptide **20** mol. weight (average neutral mass): **4049.28** (+ **11** x 114) desired amount: **10** mg

intended usage: **antimicrobial activity**

HPLC-purificat. ☒ required ☐ not necessary ☐ according to agreement

☐ exchange of counter ion TFA → HCl requested (with surcharge)

☐ repetition synthesis; our previous name:

comments: **10 % vom Rohpeptid zurückbehalten**

General remarks: We carry out the syntheses on standard scales of 50 micromoles. Product yields vary depending on peptide length and purity. If larger amounts are needed please get in touch with us. Please restrict the length of the peptide to a maximum of 30 amino acids. Slightly longer peptides may be possible upon request. Modifications like phosphorylation, biotinylation, fluorescence labelling and others are available upon request and optional surcharge. All peptides are delivered with analytical HPLC and mass analysis. Allow a production time of 2-4 weeks, depending on our order situation and the desired quality. Remark concerning mass analysis by MALDI: In addition to the molecular ion $[M+H]^+$ some peptides show the sodium salt $[M+23]^+$ (possibly as the major signal) and the potassium salt $[M+39]^+$. This is no criterion for the quality of the product. Remark concerning mass analysis by HPLC-ESI: Multiple charged mass ions are usually dominating and are no indication of decomposition of the product.

Synthesedatum:		Ansatz: **10** µmol		
Gerät:	☐ Liberty Blue	☐ Syro	☐ manuell	
Besonderheiten während der Synthese:				
Abspaltung	☐ Silan	☐ KW	☐ KCys	Dauer:
gewün. Peptid:	☐ Hauptprodukt	☐ vorhanden		
	☐ nicht detektierbar	☐ Wiederholungssynthese erforderlich		
Anzahl präparativer HPLCs:		Anzahl analytischer HPLCs:		
Ausbeute Rohpeptid:	mg	Ausbeute gereinigtes Peptid:	mg	
Datum der Fertigstellung:		☐ Preisaufschlag erforderlich, Grund:		

Auftraggeber benachrichtigt Datum per ☐ Telefon ☐ Email

☐ Peptid übergeben Datum

Empfänger:

**23Linda-14**	**KFF-rpsH_richtig**	**2.2 mg**
**K-F-F-K-F-F-K-F-F-K---c-t-c-a-t-c-t-g-t-c-amide**		
☐ **crude** ☒ **HPLC-purif.**	**MW 4049.3** (+ 11 x 114)	**Date 15.5.23**
At free N-terminus and every Arg, His & Lys peptide has TFA (MW 114) as counter ion; aqueous solutions are strongly acidic		

HZIpna-14
KFF-rpsH

Analytical data of 23Linda-14 = KFF-rpsH_richtig

Analytical HPLC of **purified peptide** (Agilent 1100 series; column Aeris Peptide C18, 3.6 µm, 100 Å, 2.1x50 mm (Phenomenex); gradient 5% to 100% ACN (0.1% trifluoroacetic acid) in 20 min, 0.7 ml/min, detection at 220 and 280 nm).

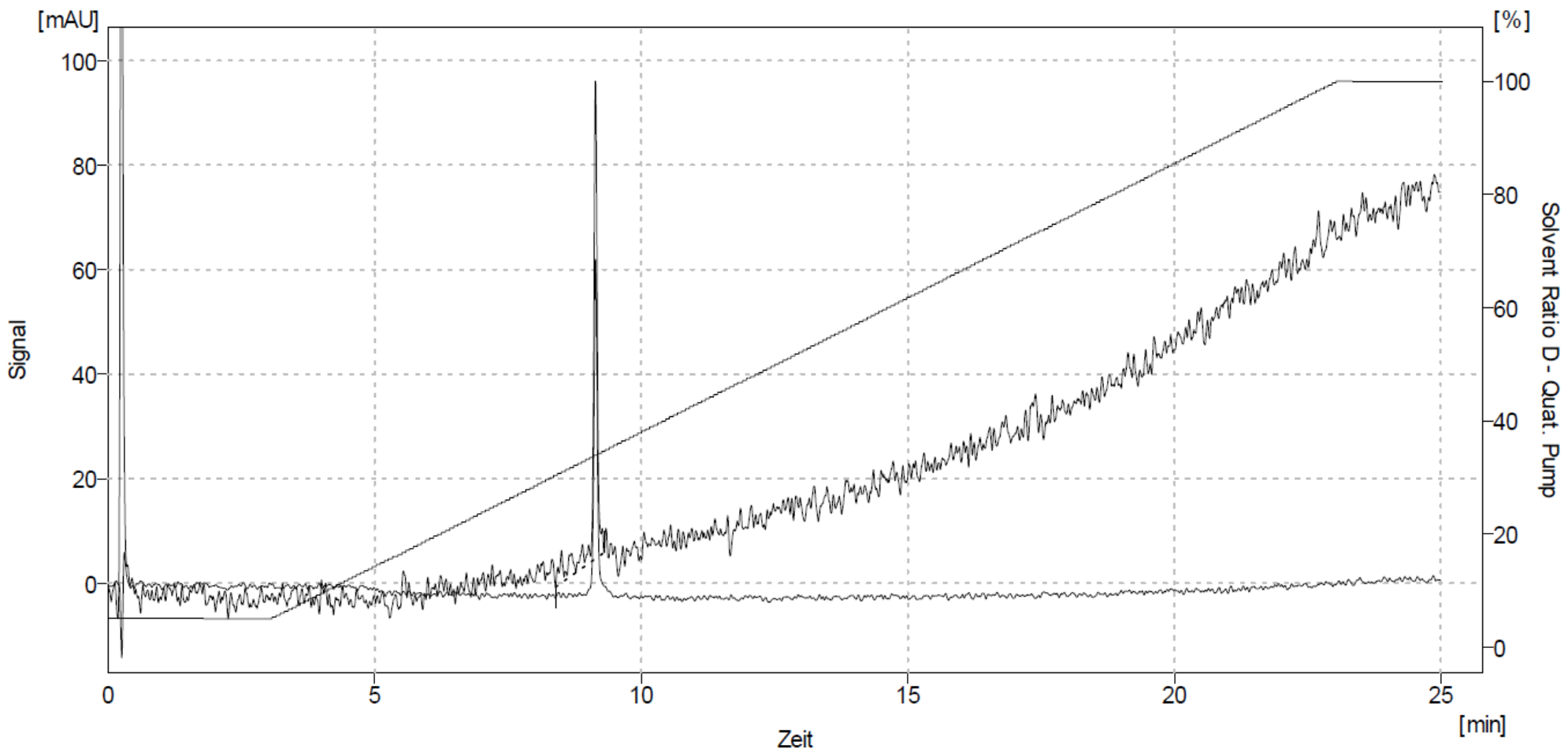

HZIpna-15
(D)KFF-acpP

Attachment for a Soluble Peptide in the Platform Peptide Synthesis

(please fill out the yellow fields and send together with the Excel order form by email to peptide@helmholtz-hzi.de)

contact person:	**Linda Popella, HIRI**	peptide name given by platform:	**23Linda-15**
phone & email:		your name of peptide:	**(D)KFF-acpP-richtig**
HZI unit:		date of placement:	**02.05.2023**

sequence: **K-F-F-K-F-F-K-F-F-K---c-t-c-a-t-a-c-t-c-t-amid**

(if labelled with *: difficult coupling according to prediction by computer program; will be added by platform)

N-terminus ☒ free ☐ acetylated ☐ biotinylated ☐ others:

C-terminus ☐ free acid ☒ amide ☐ others:

length of peptide **20** mol. weight (average neutral mass): **4033.28** (+ **11** x 114) desired amount: **10** mg

intended usage: **antimicrobial activity**

HPLC-purificat. ☒ required ☐ not necessary ☐ according to agreement

☐ exchange of counter ion TFA → HCl requested (with surcharge)

☐ repetition synthesis; our previous name:

comments: **mit D-Aminosäuren!**

General remarks: We carry out the syntheses on standard scales of 50 micromoles. Product yields vary depending on peptide length and purity. If larger amounts are needed please get in touch with us. Please restrict the length of the peptide to a maximum of 30 amino acids. Slightly longer peptides may be possible upon request. Modifications like phosphorylation, biotinylation, fluorescence labelling and others are available upon request and optional surcharge. All peptides are delivered with analytical HPLC and mass analysis. Allow a production time of 2-4 weeks, depending on our order situation and the desired quality. Remark concerning mass analysis by MALDI: In addition to the molecular ion $[M+H]^+$ some peptides show the sodium salt $[M+23]^+$ (possibly as the major signal) and the potassium salt $[M+39]^+$. This is no criterion for the quality of the product. Remark concerning mass analysis by HPLC-ESI: Multiple charged mass ions are usually dominating and are no indication of decomposition of the product.

Synthesedatum:		Ansatz: **10** µmol		
Gerät:	☐ Liberty Blue	☐ Syro	☐ manuell	
Besonderheiten während der Synthese:				
Abspaltung	☐ Silan	☐ KW	☐ KCys	Dauer:
gewün. Peptid:	☐ Hauptprodukt	☐ vorhanden		
	☐ nicht detektierbar	☐ Wiederholungssynthese erforderlich		
Anzahl präparativer HPLCs:		Anzahl analytischer HPLCs:		
Ausbeute Rohpeptid:	mg	Ausbeute gereinigtes Peptid:	mg	
Datum der Fertigstellung:		☐ Preisaufschlag erforderlich, Grund:		

Auftraggeber benachrichtigt Datum per ☐ Telefon ☐ Email

☐ Peptid übergeben Datum

Empfänger:

**23Linda-15**	**(D)KFF-acpP-richtig**	**13.3 mg**
**K-F-F-K-F-F-K-F-F-K---c-t-c-a-t-a-c-t-c-t-amid**		
☐ crude ☒ **HPLC-purif.**	**MW 4033.3** (+ 11 x 114)	**Date 15.5.23**
At free N-terminus and every Arg, His & Lys peptide has TFA (MW 114) as counter ion; aqueous solutions are strongly acidic		

HZIpna-15
(D)KFF-acpP

Analytical data of 23Linda-15 = (D)KFF-acpP-richtig

Analytical HPLC of **purified peptide** (Agilent 1100 series; column Aeris Peptide C18, 3.6 µm, 100 Å, 2.1x50 mm (Phenomenex); gradient 5% to 100% ACN (0.1% trifluoroacetic acid) in 20 min, 0.7 ml/min, detection at 220 and 280 nm).

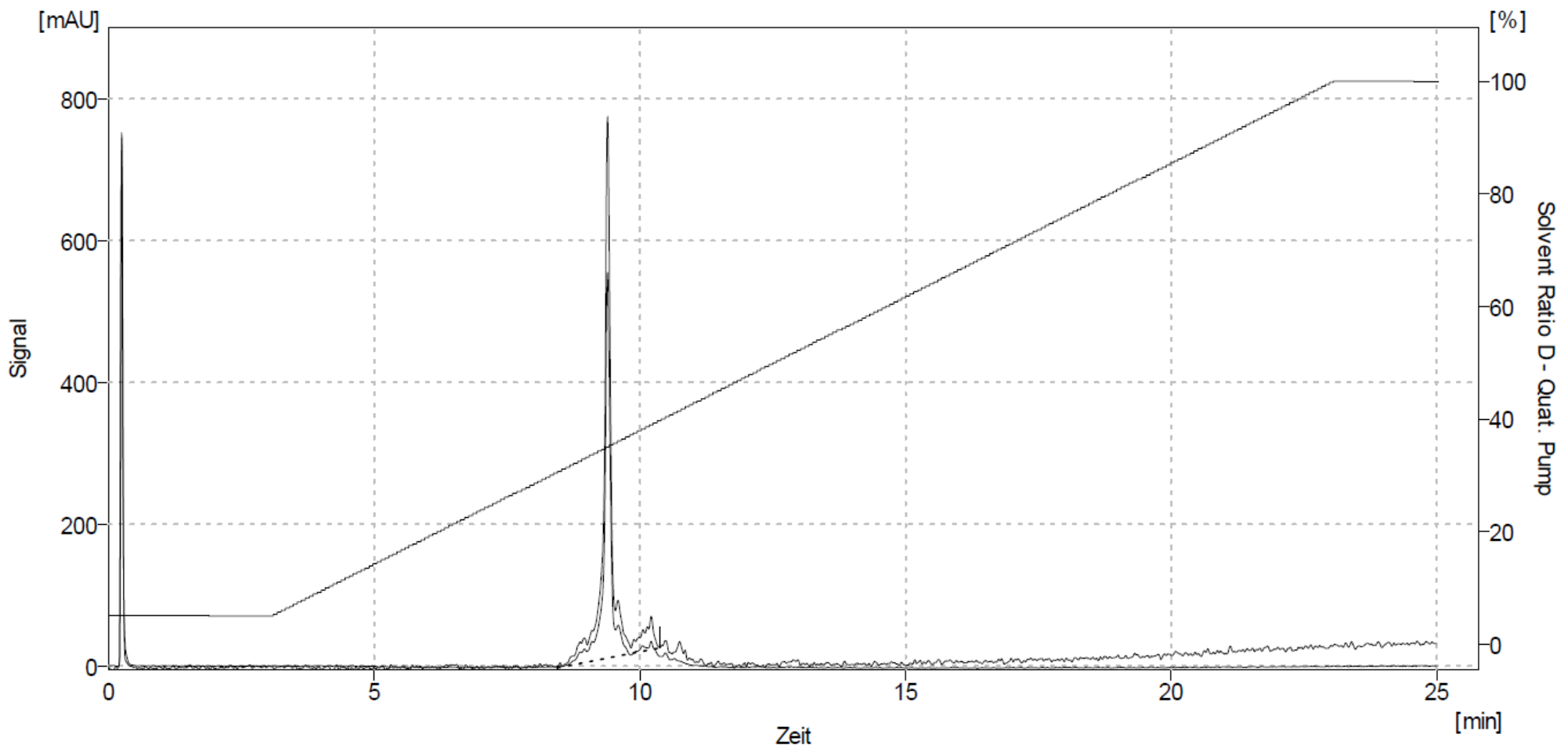

HZIpna-16
RXR-acpP (P. aeruginosa)

Attachment for a Soluble Peptide in the Platform Peptide Synthesis

(please fill out the yellow fields and send together with the Excel order form by email to peptide@helmholtz-hzi.de)

contact person:	**Linda Popella, HIRI**	peptide name given by platform:	**23Linda-16**
phone & email:		your name of peptide:	**RXR-acpP_Pa**
HZI unit:		date of placement:	**02.05.2023**

sequence: **R-(Ahx)-R-R-(Ahx)-R-R-(Ahx)-R-R-(Ahx)-R-(Ahx)-ßAla---c-t-c-a-t-a-c-c-t-t-g-amid**

(if labelled with *: difficult coupling according to prediction by computer program; will be added by platform)

N-terminus ☒ free ☐ acetylated ☐ biotinylated ☐ others:

C-terminus ☐ free acid ☒ amide ☐ others:

length of peptide **25** mol. weight (average neutral mass): **4815.24** (+ **16** x 114) desired amount: **10** mg

intended usage:

HPLC-purificat. ☒ required ☐ not necessary ☐ according to agreement

☐ exchange of counter ion TFA → HCl requested (with surcharge)

☐ repetition synthesis; our previous name:

comments: **10 % vom Rohpeptidzurückbehalten**

General remarks: We carry out the syntheses on standard scales of 50 micromoles. Product yields vary depending on peptide length and purity. If larger amounts are needed please get in touch with us. Please restrict the length of the peptide to a maximum of 30 amino acids. Slightly longer peptides may be possible upon request. Modifications like phosphorylation, biotinylation, fluorescence labelling and others are available upon request and optional surcharge. All peptides are delivered with analytical HPLC and mass analysis. Allow a production time of 2-4 weeks, depending on our order situation and the desired quality. Remark concerning mass analysis by MALDI: In addition to the molecular ion $[M+H]^+$ some peptides show the sodium salt $[M+23]^+$ (possibly as the major signal) and the potassium salt $[M+39]^+$. This is no criterion for the quality of the product. Remark concerning mass analysis by HPLC-ESI: Multiple charged mass ions are usually dominating and are no indication of decomposition of the product.

Synthesedatum:		Ansatz: **10** µmol	
Gerät:	☐ Liberty Blue	☐ Syro	☐ manuell
Besonderheiten während der Synthese:			
Abspaltung	☐ Silan	☐ KW	☐ KCys Dauer:
gewün. Peptid:	☐ Hauptprodukt	☐ vorhanden	
	☐ nicht detektierbar	☐ Wiederholungssynthese erforderlich	
Anzahl präparativer HPLCs:		Anzahl analytischer HPLCs:	
Ausbeute Rohpeptid:	mg	Ausbeute gereinigtes Peptid:	mg
Datum der Fertigstellung:		☐ Preisaufschlag erforderlich, Grund:	

Auftraggeber benachrichtigt Datum per ☐ Telefon ☐ Email

☐ Peptid übergeben Datum

Empfänger:

**23Linda-16**	**RXR-acpP_Pa**	**56 mg**
**R(Ahx)RR(Ahx)RR(Ahx)RR(Ahx)R(Ahx)-ßAla-c-t-c-a-t-a-c-c-t-t-g-amide**		
☐ **crude** ☒ **HPLC-purif.**	**MW 4815.2** (+ 16 x 114)	**Date 22.5.23**
At free N-terminus and every Arg, His & Lys peptide has TFA (MW 114) as counter ion; aqueous solutions are strongly acidic		

HZIpna-16
RXR-acpP (P. aeruginosa)

Analytical data of 23Linda-16 = RXR-acpP_Pa

Analytical HPLC of **purified peptide** (Agilent 1100 series; column Aeris Peptide C18, 3.6 µm, 100 Å, 2.1x50 mm (Phenomenex); gradient 5% to 100% ACN (0.1% trifluoroacetic acid) in 20 min, 0.7 ml/min, detection at 220 and 280 nm).

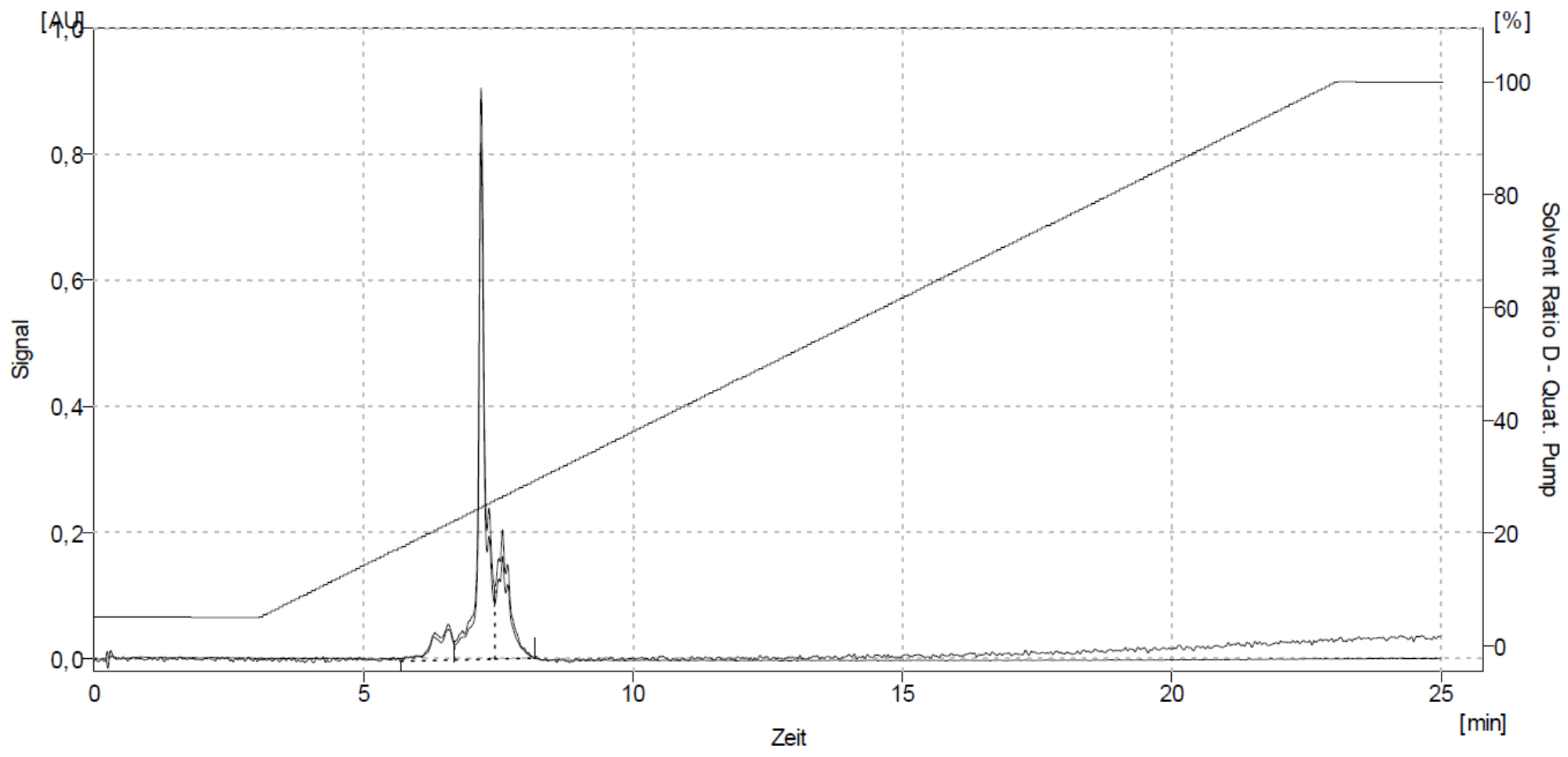

LC-ESI-MS (Bruker Amazon); HPLC Agilent 1200 series, column Gemini-NX C18, 3 µm, 110 Å, 2.0x50 mm (Phenomenex); gradient 5-95% ACN (0.1% formic acid) in 19 min, 0.4 ml/min, detection at 220 nm.
Expected: 4815.24 (average neutral mass)

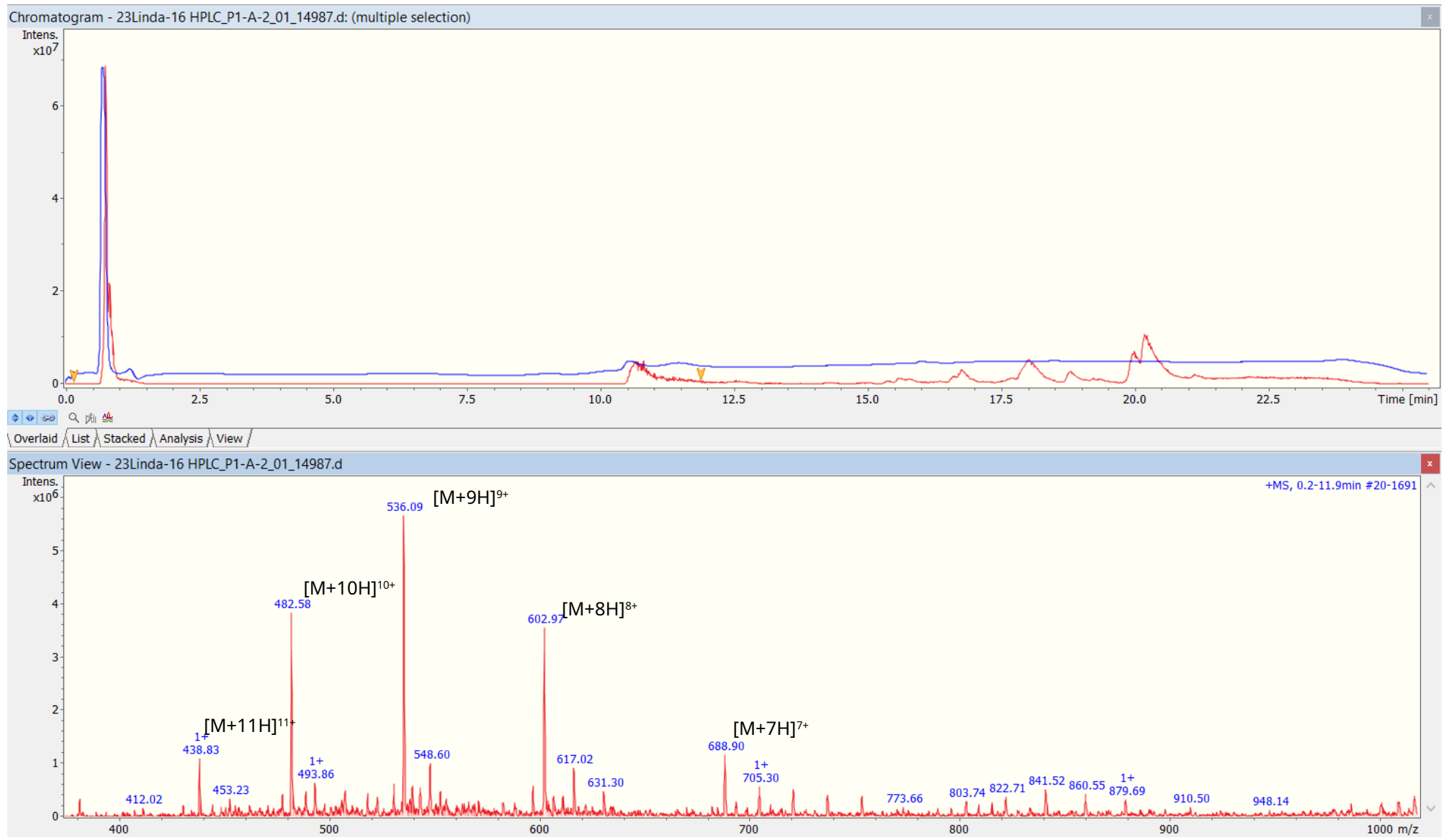

Peps4LS GmbH
Im Neuenheimer Feld 518
69120 Heidelberg
Telefon: +49 6221 6514511
Mail: Jacob@peps4ls.de

Injection Date : 21.03.25
Sample Name : S1185_A1
Operator : Jacob

Vial : P1-A-03
Inj Volume : 3µl

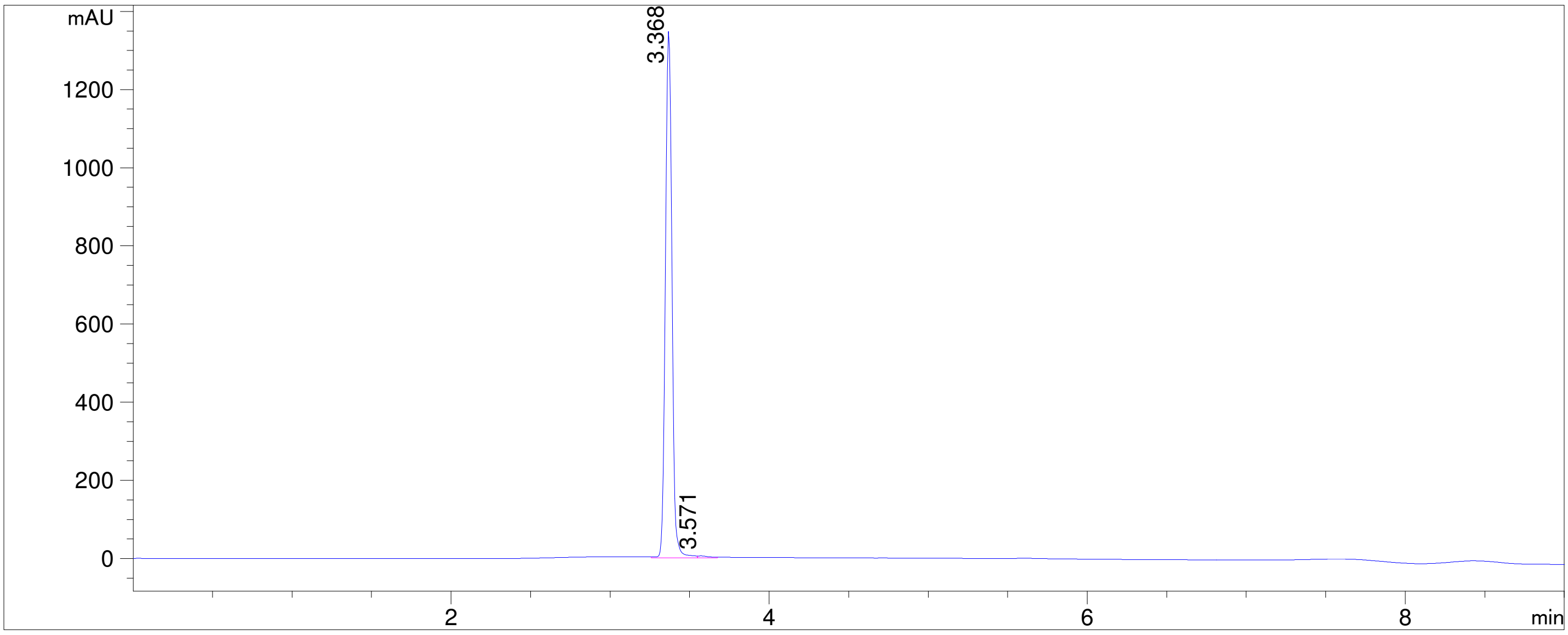

Area Percent Report

Signal 1 : DAD1 A, Sig=217,4 Ref=off
Calculation : Area
Sorted By : Retention Time

#	Meas. R	Peak T	Area	Height	Area %
1	3.368	VV	3.909e3	1.349e3	99.411
2	3.571	MF	23.165	4.880	0.589

*** End of Report ***

Peps4LS GmbH
Im Neuenheimer Feld 518
69120 Heidelberg
Telefon: +49 6221 6514511
Mail: Jacob@peps4ls.de

Injection Date : 25.03.2025
Sample Name : S1185_A2
Operator : Jacob

Vial : P1-A-02
Inj Volume : 3µl

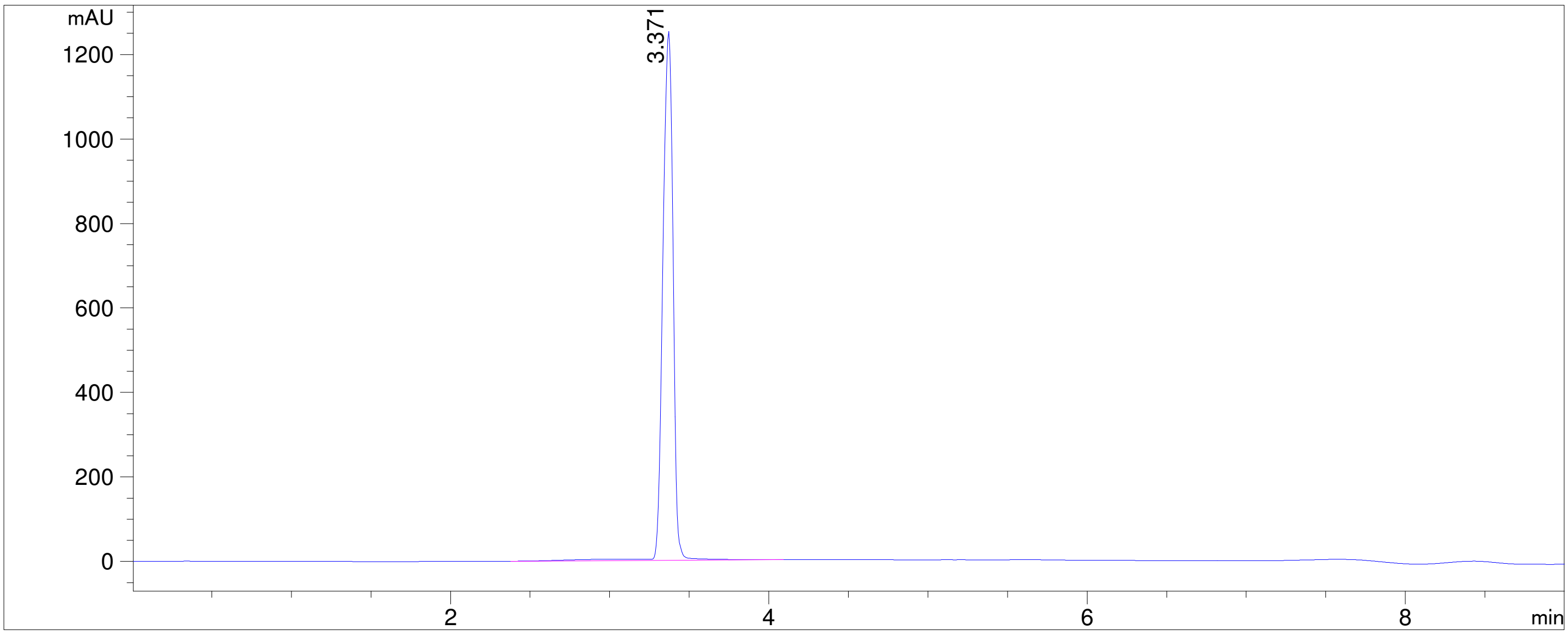

Area Percent Report

Signal 1 : DAD1 A, Sig=217,4 Ref=off
Calculation : Area
Sorted By : Retention Time

#	Meas. R	Peak T	Area	Height	Area %
1	3.371	BB	5.708e3	1.253e3	100.000

*** End of Report ***

Peps4LS GmbH
Im Neuenheimer Feld 518
69120 Heidelberg
Telefon: +49 6221 6514511
Mail: Jacob@peps4ls.de

Qualitätskontrolle S1185_C2

Injection Date : 25.03.2025 Vial : P1-A-03
Sample Name : S1185_C2 Inj Volume : 3µl
Operator : Jacob

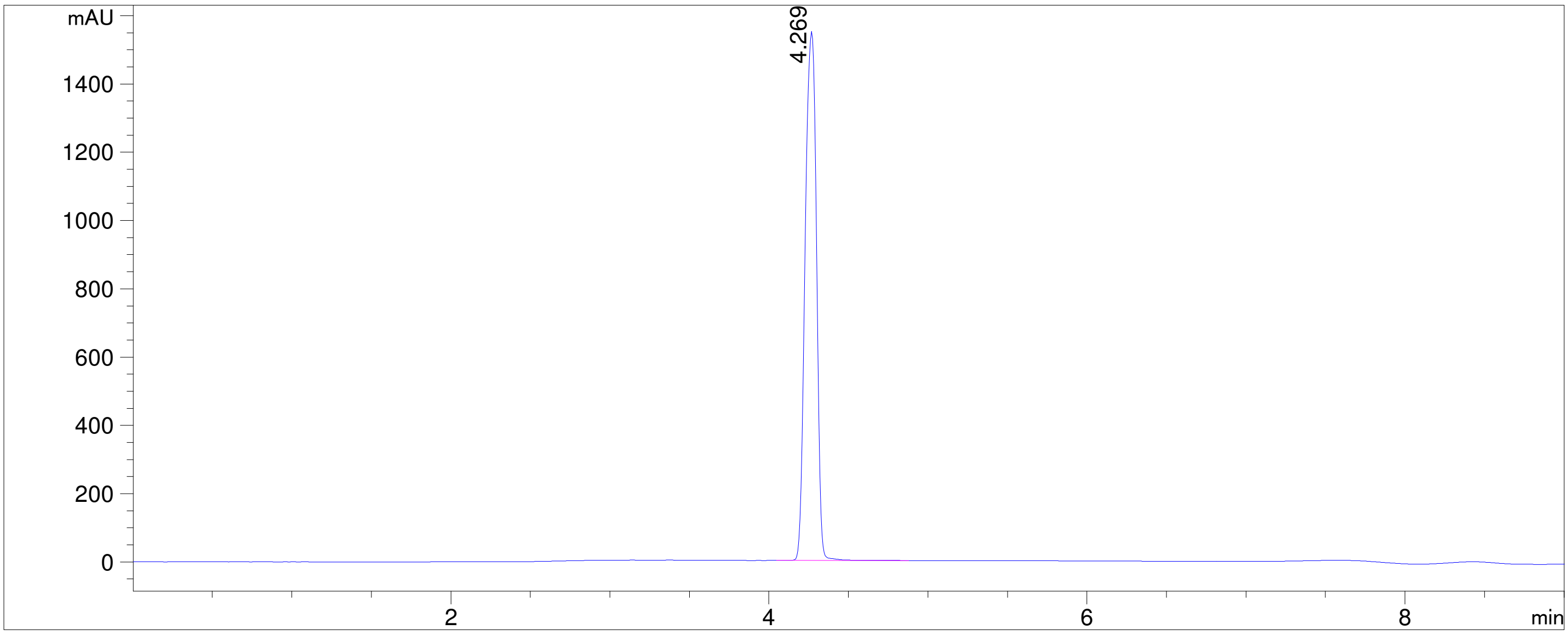

Area Percent Report

Signal 1 : DAD1 A, Sig=217,4 Ref=off
Calculation : Area
Sorted By : Retention Time

#	Meas. R	Peak T	Area	Height	Area %
1	4.269	BB	7.729e3	1.549e3	100.000

*** End of Report ***

Peps4LS GmbH
Im Neuenheimer Feld 518
69120 Heidelberg
Telefon: +49 6221 6514511
Mail: Jacob@peps4ls.de

Injection Date	: 21.03.25	Vial	: P1-A-04
Sample Name	: S1185_E1	Inj Volume	: 3µl
Operator	: Jacob		

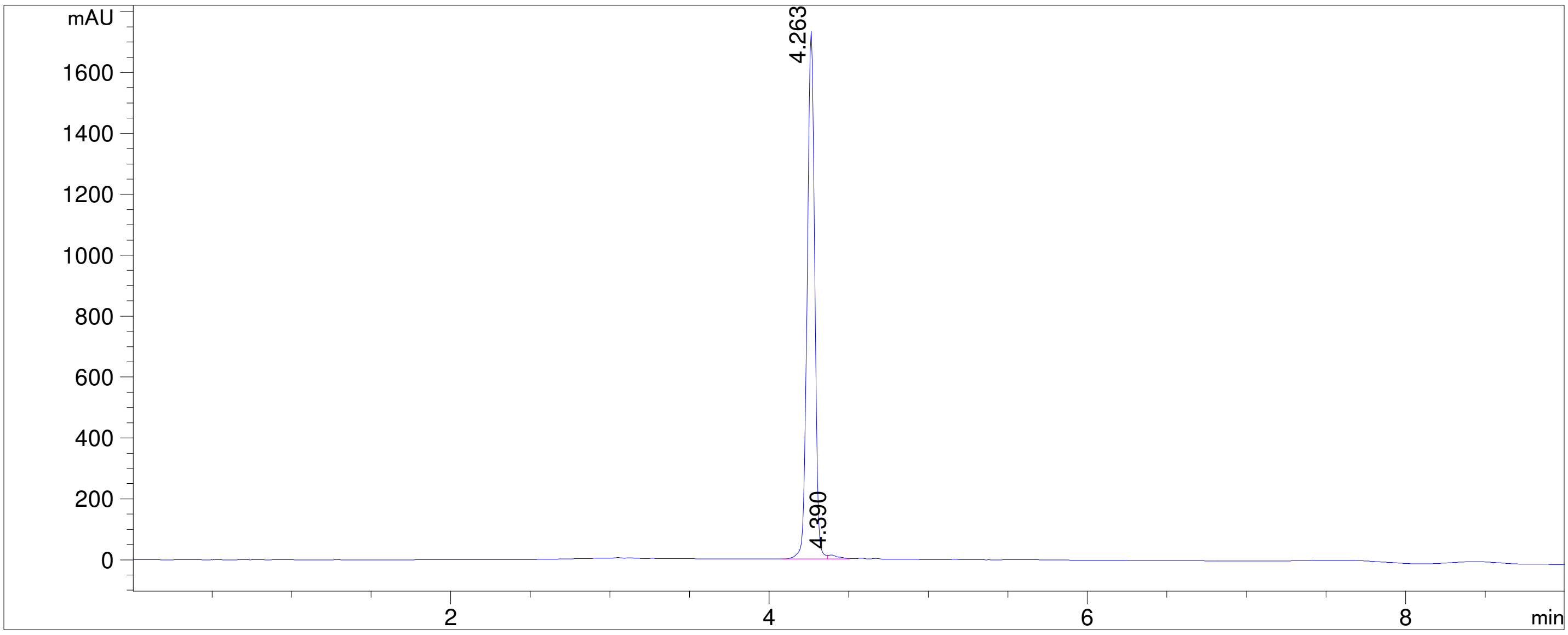

Area Percent Report

Signal 1 : DAD1 A, Sig=217,4 Ref=off
Calculation : Area
Sorted By : Retention Time

#	Meas. R	Peak T	Area	Height	Area %
1	4.263	BV	5.640e3	1.732e3	98.998
2	4.390	VV	57.082	12.498	1.002

*** End of Report ***

Peps4LS GmbH
Im Neuenheimer Feld 518
69120 Heidelberg
Telefon: +49 6221 6514511
Mail: Jacob@peps4ls.de

Qualitätskontrolle S1187_A1

Injection Date : 21.03.25
Sample Name : S1187_A1
Operator : Jacob

Vial : P1-A-05
Inj Volume : 3µl

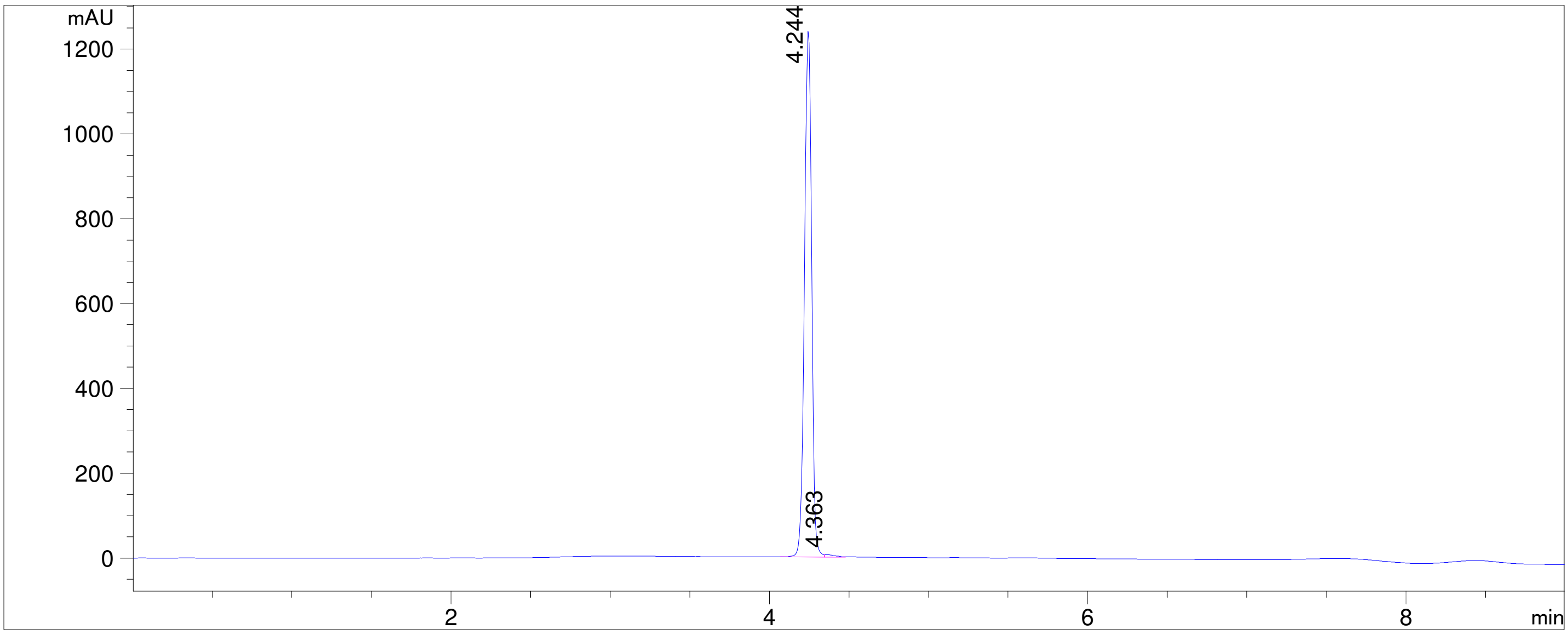

Area Percent Report

Signal 1 : DAD1 A, Sig=217,4 Ref=off
Calculation : Area
Sorted By : Retention Time

#	Meas. Ret. Time	Peak Type	Area	Height	Area %
1	4.244	BV	3.798e3	1.243e3	99.295
2	4.363	MF	26.963	6.234	0.705

*** End of Report ***

Peps4LS GmbH
Im Neuenheimer Feld 518
69120 Heidelberg
Telefon: +49 6221 6514511
Mail: Jacob@peps4ls.de

Qualitätskontrolle S1187_A8

Injection Date : 25.03.2025
Sample Name : S1187_A8
Operator : Jacob

Vial : P1-A-08
Inj Volume : 3µl

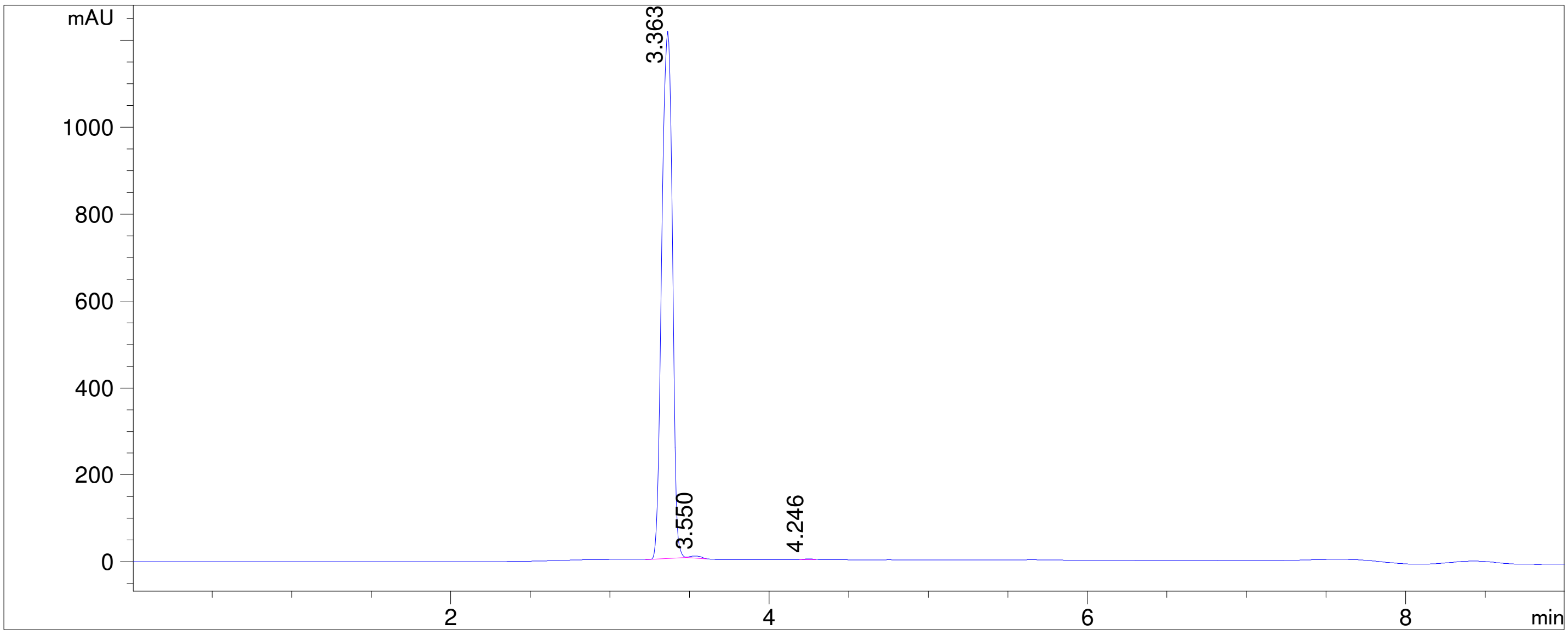

Area Percent Report

Signal 1 : DAD1 A, Sig=217,4 Ref=off
Calculation : Area
Sorted By : Retention Time

#	Meas. R	Peak T	Area	Height	Area %
1	3.363	MM	5.662e3	1.215e3	99.473
2	3.550	MM	23.291	4.870	0.409
3	4.246	MM	6.719	1.571	0.118

*** End of Report ***

Peps4LS GmbH
Im Neuenheimer Feld 518
69120 Heidelberg
Telefon: +49 6221 6514511
Mail: Jacob@peps4ls.de

Qualitätskontrolle S1187_B1

Injection Date : 25.03.2025
Sample Name : S1187_B1
Operator : Jacob

Vial : P1-A-07
Inj Volume : 3µl

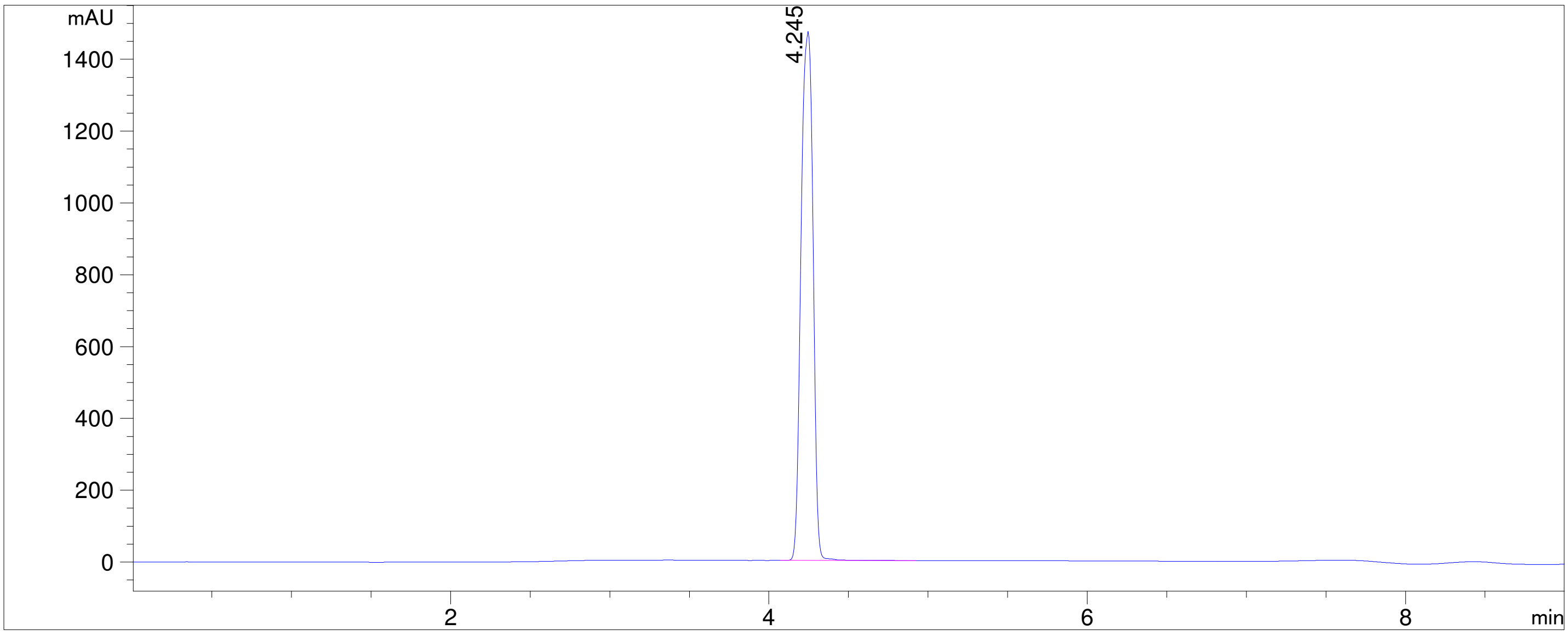

Area Percent Report

Signal 1 : DAD1 A, Sig=217,4 Ref=off
Calculation : Area
Sorted By : Retention Time

#	Meas. R	Peak T	Area	Height	Area %
1	4.245	BB	7.606e3	1.473e3	100.000

*** End of Report ***

Peps4LS GmbH, INF 518, 69120 Heidelberg

Person in charge: Dr. Anette Jacob
Fon: +49 6221 6514511
Mail: Jacob@Peps4LS.de

Heidelberg, den 28.04.2022

Sequence data for L220428-3 - Marco Galardini

Product	Sequence	$[M+H]^+$ [Da]	$[nmol]_{260}$	Purity [%]	ID-Nr
JVpna-21/KFF-acpP	KFFKFFKFFK-ctcatactct	4031,84	1269	99,51	S976-A1

The product is HPLC purified. Please find enclosed Maldi MS analysis and analytical HPLC spectrum of the CPP-PNA conjugate for quality control. Maldi-MS spectra often show the $[M+2H]^{2+}$-signal at half of the mass on the m/z axis additionally to the $[M+H]^+$- and/or the $[M+Na]^+$-signal of the molecular ion of the product.

Quantification of the PNA-peptide conjugate was carried out by UV absorption at 260nm. $[nmol]_{260}$-amounts given in the table are calculated by the UV absorption of the bases at 260nm. Calculated nmol resulting from balanced weights of the compounds may differ from that due to the weight of counterions of the functional groups in the side chains of the peptides. Thus the compounds shall be completely dissolved to get the right concentration. Stock solutions of the conjugates can be stored at -20°C (long time storage).

Purity [%] was determined by analytical HPLC using a standard gradient.

Peps4LS GmbH,
Im Neuenheimer Feld 518, 69120 Heidelberg
Amtsgericht Mannheim HRB 710063
Geschäftsführung: Dr. Anette Jacob
USt.-ID: DE 273653454

Postbank Stuttgart
Konto-Nr. 481 158 706
BLZ 600 100 70
BIC: PBNKDEFF
IBAN: DE56 6001 0070 0481 1587 06

Mail: Jacob@Peps4LS.de
Mobile: +49 15253591203
Fon: +49 6221 6514511
Fax: +49 6221 6548466

AB Sciex TOF/TOF™ Series Explorer™ 2049

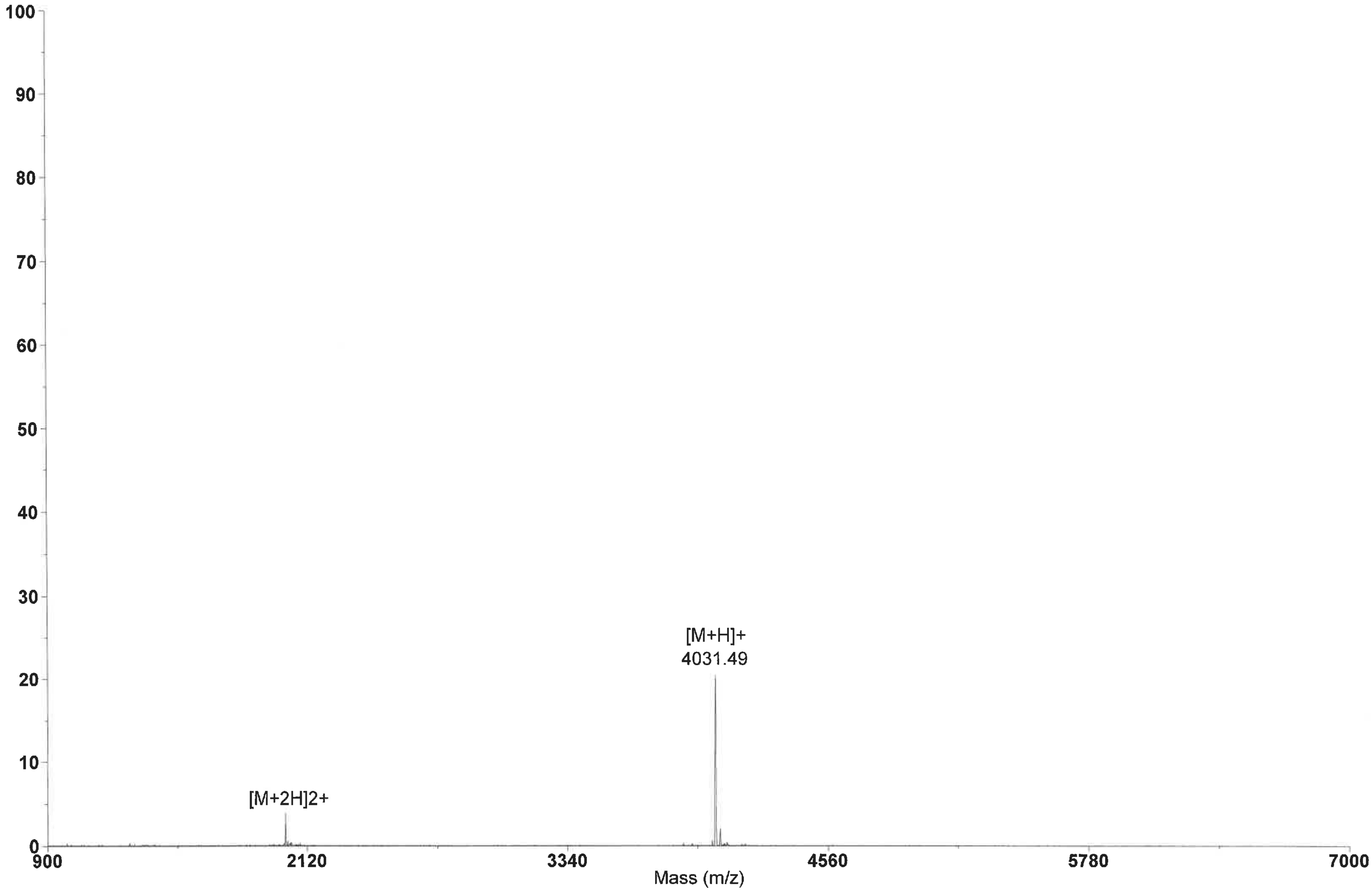

Printed: 10:06, April 28, 2022

C:\Documents and Settings\Maldi\My Documents\LabVIEW Data\S976\S976_A1_.T2D

Peps4LS GmbH
Im Neuenheimer Feld 518
68120 Heidelberg
Telefon: +49 6221 6514511
Mail: Jacob@peps4ls.de

Injection Date : 27.04.2022
Sample Name : S976_A1
Operator : Jacob

Vial : P1-C-01
Inj Volume : 5µl

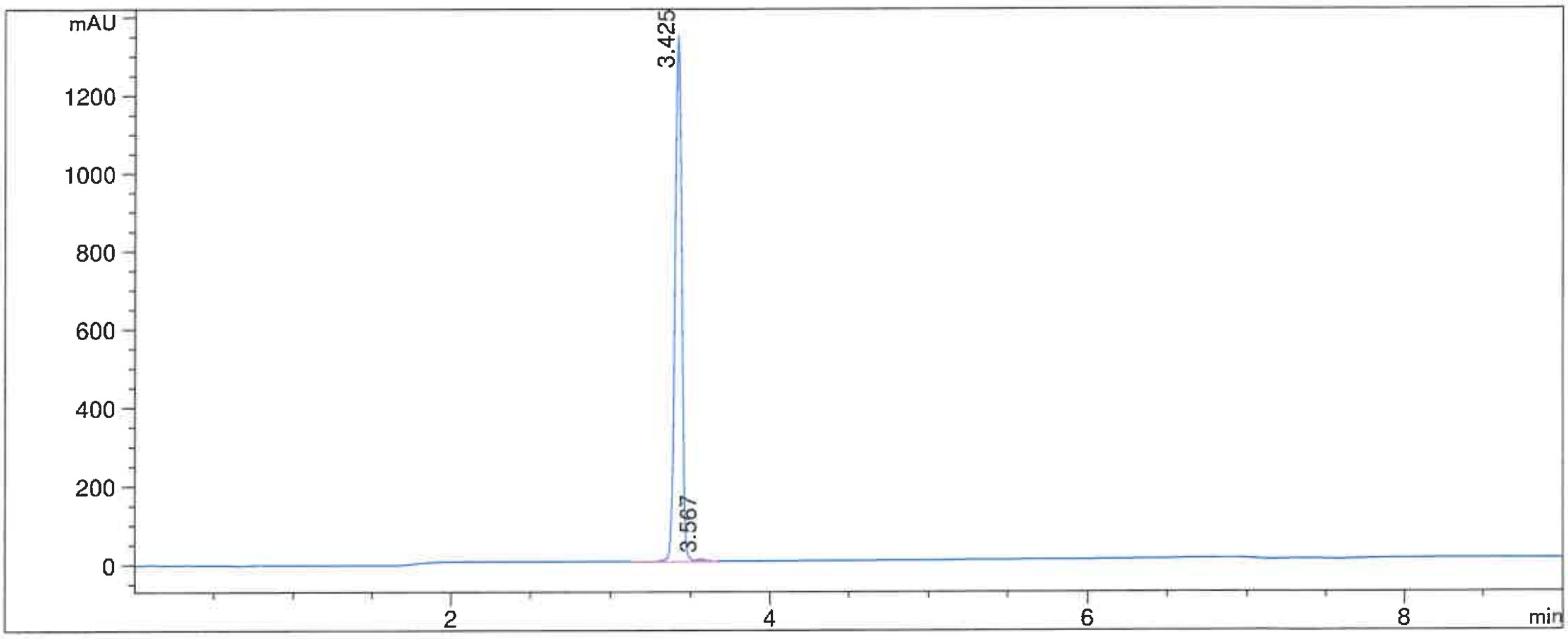

Area Percent Report

Signal 1 : DAD1 A, Sig=217,4 Ref=off
Calculation : Area
Sorted By : Retention Time

#	Meas. Peak	R T	Area	Height	Area %
1	3.425	BV	3.886e3	1.344e3	99.509
2	3.567	VV	19.175	5.055	0.491

*** End of Report ***
