# Supplementary material for: A systematic identification of resistance determinants to antisense antibiotics suggests adaptation strategies dependent on the delivery peptide
Source: Nat Commun. 2026 Aug 12;17:8262. doi: 10.1038/s41467-026-76357-y (PMC13469510; doi:10.1038/s41467-026-76357-y)
Supplement: Supplementary file 2 [file 41467_2026_76357_MOESM2_ESM.pdf]

Title: Supplementary Data 1.

Description: Minimum inhibitory concentration (MIC) values for ASO formulations for each ancestral strain and adapted isolates.

Title: Supplementary Data 2.

Description: List of genetic variants identified in each sample adapted to the ASO formulations, after filtering.

Title: Supplementary Data 3.

Description: List of genetic variants identified in each sample adapted to the scrambled ASO formulations, after filtering.

Title: Supplementary Data 4.

Description: List of asobiotics and their target sequences across the bacterial species tested in this study.

Title: Supplementary Data 5.

Description: List of strains and primers used for the validation of selected mutants.
