# Supplementary material for: A systematic identification of resistance determinants to antisense antibiotics suggests adaptation strategies dependent on the delivery peptide
Source: Nat Commun. 2026 Aug 12;17:8262. doi: 10.1038/s41467-026-76357-y (PMC13469510; doi:10.1038/s41467-026-76357-y)
Supplement: Supplementary file 4 [file 41467_2026_76357_MOESM9_ESM.pdf]

nature portfolio

Peer Review File

A systematic identification of resistance determinants to antisense antibiotics suggests adaptation strategies dependent on the delivery peptide

Corresponding Author: Professor Marco Galardini

This file contains all reviewer reports in order by version, followed by all author rebuttals in order by version.

Version 0:

Reviewer comments:

Reviewer #1

(Remarks to the Author)
In this study Galardine et al. have analyzed resistance development against antisense BPP-PNAs in four Gram negative bacterial species (E.coli, K. pneumonia, S. enterica and P aeruginosa) by serial passaging evolution, with special focus on the effect of the delivery peptide. The experiments are focussed on acpP as the antisense target, and the well described "KFF" and "RXR" delivery peptides (BPPs).
Apart from the expected resistance from sbmA mutations, a large variety of other gene mutations were identified in the evolved populations. Possible mechanisms by which these may induce resistance either via interference with PNA target binding or inhibition of translation, or by reduction of BPP dependent cellular uptake is discussed, but none of the theories are validated by specific genetic validation or other studies.
Therefore, the study is predominantly descriptive (comprehensive) data presentation.
In addition, although effective bacterial delivery of antisense PNAs and PMOs is a key, activity-limiting process for antisense based antibiotics drug discovery, the relevance of the present study is rather limited in this context, due to the choice of CPPs.
The fundamentally different bacterial uptake processes (in E. coli) for both KFF and RXR PNA conjugates has already been studied identifying the non-essential peptide transporter sbmA as necessary for inner membrane crossing of KFF-PNA (refs. 11 & 13), and its functional absence causing resistance against antisense KFF-PNAs. In contrast, RXR-PNA antisense compounds do not require sbmA for inner membrane crossing, but is dependent on at functional inner membrane potential to drive the crossing, and more than one mutation is required to reduce this potential (ref. 15). Therefore, in a serious drug discovery/development process, the KFF peptide will not be relevant due to the easy resistance development by sbmA mutations. As previously shown (ref 15), and supported by the present data resistance development against RXR conjugates is much more complex and difficult. However, these conjugates may have toxicity issues (e.g. Bioconjugate Chem. 2007, 18, 1325).
Therefore, the present study unfortunately provides limited new knowledge that will guide the antisense antibacterial drug discovery field.

Other points
1. The more detailed mechanistic interpretation of the genetic data is limited by the lack of data from isolated resistant clones, and lack of data on the effect of mutations in single genes and in specific combinations of genes.
2. KFF- and RXR-PNA conjugates can also exhibit gene target independent, direct antibacterial effects analogous to those of antibacterial peptides, as observed by antibacterial activity of e.g. mismatch controls (at higher concentration). Thus inclusion of such mismatch controls could shed light on this mechanism.
3. Line 78: PMOs do not have a pseudopeptide backbone
4. Line 95-96: SbmA does not confer resistance to KFF conjugates, on the contrary, it is required for activity. Indeed the argumentation in lines 94-99 does not make much sense.
5. Supplementary tables are missing

(Remarks on code availability)

Reviewer #2

(Remarks to the Author)
Mulkern et al. aim to investigate the mechanisms of resistance to antisense peptide nucleic acids (PNAs) through in vitro laboratory evolution and genome sequencing of the evolved clones.

Experimental Design and Key Objectives:

Primary Resistance Mechanism:
To investigate the primary resistance mechanism against a $(KFF)_3$-K–conjugated PNA targeting acpP (KFF-acpP), the authors evolved 10 biological replicates from three Gram-negative pathogens: Escherichia coli (UPEC), Klebsiella pneumoniae, and Salmonella enterica and a lab E. coli strain (MG1655) under selective pressure with KFF-acpP.

Identifying Additional Genetic Factors:
To identify genes influencing PNA activity beyond the well-known role of sbmA (the transporter responsible for $(KFF)_3$-K–conjugated PNA uptake), an sbmA knockout strain (ΔsbmA) was evolved against the same $(KFF)_3$-K–conjugated PNA targeting acpP.

Impact of Different CPPs on Resistance:
To examine how different cell-penetrating peptides (CPPs) affect PNA resistance and evolvability, a laboratory E. coli strain (MG1655) was evolved against acpP PNA conjugated with three distinct CPPs: the original L-form of $(KFF)_3$-K, its D-form (D-$(KFF)_3$-K), and $(RXR)_4$XB.

Comparing Resistance Mechanisms for Different PNAs:
Finally, to compare resistance mechanisms, the authors evolved a laboratory E. coli strain against two PNAs targeting acpP and rpsH, both conjugated with $(KFF)_3$-K.

Key Findings:
Mutations in sbmA and yaiW:
Loss-of-function mutations in the sbmA gene—a known mediator of $(KFF)_3$-K–conjugated PNA uptake—were common across all three tested Gram-negative pathogens. Additionally, yaiW was identified as a potential contributor to $(KFF)_3$-K-PNA uptake. Notably, yaiW is co-transcribed with sbmA and a palmitate-modified, surface-exposed outer membrane lipoprotein.

Species-Specific Mutations:
Other potentially species-specific mutations were observed following treatment with $(KFF)_3$-K–conjugated acpP, such as mutations in rfbF gene of S. enterica lines (which may impact CPP binding and penetration) and in mdtC gene of the K. pneumoniae lines (a component of the mdtABC/tolC multidrug efflux pump).

Role of ykfM:
Variants in the uncharacterized transmembrane gene ykfM were consistently identified across all replicates of both MG1655 and ΔsbmA strains adapted to $(KFF)_3$-K–acpP. Therefore, the authors suggest that ykfM indirectly affects the efficacy of the PNA targeting acpP.

Delivery Method Influences Resistance:
The study demonstrates that the delivery method is crucial; the acpP PNA conjugated with $(KFF)_3$-K showed a higher evolution of resistance compared to conjugates with D-$(KFF)_3$-K or $(RXR)_4$XB.

Ribosome Rescue Mechanisms:
In a highly resistant sample (16-fold MIC increase for KFF–acpP), a mutation (H246L) in the prfB gene was identified. Similarly, a variant in the related prfA gene was observed in an evolved UPEC strain. Since both genes are involved in rescuing stalled ribosomes, the authors propose two potential hypotheses for the cellular response to PNAs.

Rare Mutation Within The PNA Target Window
For RXR–acpP, only a relatively low-frequency (~30%) set of SNPs was identified within the core of the PNA target window. This represents the first evidence that rare mutations within this central binding region may reduce the efficacy of the PNA.

Major General Comments:

- Clarity and Logical Flow: The paper is challenging to follow due to its lack of clear logical flow. Since not all possible combinations of CPP-PNAs and bacterial strains were screened, the specific objectives of each evolution experiment remain unclear. I recommend reorganizing the paper around the four main experimental goals (as outlined above) to better clarify the purpose and provide a more focused discussion of the genetic variants observed.
- Additionally, the authors might consider reintroducing some of the most interesting and novel genetic variants into wild-type lab E. coli strains to further support their observations and hypotheses.

Figure 1A:
Figure 1A is somewhat misleading, as it implies that all listed strains were evolved with all CPP-PNA combinations. A table

that clearly details which evolution experiments were performed for each strain and CPP-PNA combination would be very helpful.

Selection Pressure Concerns:
The experimental design (starting at 0.5 MIC and increasing by 2-fold every fourth step over 16 passages) and the high frequency of synonymous mutations (an average of 15 SNPs per sample, lines 187-189) suggest that the selection pressure might have been relatively mild. This could lead to a higher frequency of neutral mutations and might not fully capture the genetic adaptations that would occur under stronger, more clinically relevant conditions. The authors should discuss how this might affect the robustness of their conclusions.

Role of fimE:
The authors note that fimE, involved in fimbrial biosynthesis, was frequently mutated in E. coli MG1655 adapted to all KFF-conjugated PNAs and in K. pneumoniae evolved to KFF–acpP. However, they overlook that fimE was also the most frequently mutated gene in lab E. coli adapted to D-KFF–acpP (as shown in Figure 3B), but not in strains evolved with RXR–acpP. Could the authors provide further insight into whether fimE might play a role in the uptake of KFF and D-KFF conjugated PNAs?

Additional Comments:

yaiW: Why was yaiW frequently mutated only in the lab E. coli strain (MG1655) evolved to $(KFF)_3$-K–acpP or $(KFF)_3$-K–rpsH and not in other Gram-negative pathogens evolved to $(KFF)_3$-K–acpP?
Species-Specific Mutations: Are the observed mutations in rfbF and mdtC truly species-specific, given previous links to reduced PNA efficacy in E. coli (lines #224–227, #362–364)?

Resistance Comparison against acpP and rpsH PNAs: Can the authors provide any further explanation for why the laboratory E. coli strain (MG1655) developed lower resistance to $(KFF)_3$K–rpsH compared to $(KFF)_3$K–acpP?

ykfM: The authors suggest that the uncharacterized transmembrane gene ykfM indirectly affects the efficacy of PNA targeting acpP. However, it is notable that ykfM mutations were observed only in the laboratory E. coli strains and not in other Gram-negative pathogenic strains. Could the authors provide any further explanation for this observation? Reintroducing the ykfM mutation into a wild-type lab E. coli background and assessing its resistance to both $(KFF)_3$-K–acpP and $(KFF)_3$-K–rpsH would greatly strengthen this hypothesis

prfA/prfB Mutations: The authors propose that mutations in prfA and prfB (which rescue stalled ribosomes) may confer resistance to PNAs. Are these resistance mechanisms independent of PNA or CPP? Reintroducing these mutations into appropriate strain backgrounds and testing resistance against different KFF-conjugated PNAs (e.g., acpP vs. rpsH, or D-KFF–acpP vs. RXR–acpP) would provide valuable insights.
RXR Binding Site Mutations: For P. aeruginosa, did the authors find any mutations at the center of the RXR–acpP binding site among the evolved strains, which exhibited a 2-fold increase in MIC?

Comments on Figure 2:
Clarity and Data Presentation: Figure 2 could be improved for better clarity. Several data points overlap, making it difficult to distinguish individual results. Applying jittering on both axes would help separate these points and enhance visibility. Additionally, reorganizing the data could improve readability. It would be helpful to separate the results for pathogenic strains treated with KFF–rpsH and RXR–acpP (e.g., for P. aeruginosa) from those for laboratory E. coli strains (MG1655 and ΔsbmA) treated with various CPP-PNAs. Currently, the results for the lab E. coli and UPEC strains appear in both the left and right panels, which can be confusing. Grouping similar data together in dedicated panels would enhance clarity. Furthermore, indicating which pathogen strains evolved statistically significantly higher or lower resistance to KFF–acpP, as well as highlighting which CPP-PNAs induced significantly stronger or lower resistance in the lab E. coli strains, would provide valuable insights and make the figure more informative.

Conclusion:
Overall, the reported results are both important and novel, given the current lack of empirical data on the adaptation strategies to asobiotic treatments across bacterial pathogens. Addressing the points above—particularly clarifying the experimental design, reorganizing the presentation of data (especially for Figures 1A and 2), and further exploring specific genetic variants (such as those in ykfM and prfB, prfA)—would significantly enhance the manuscript.

(Remarks on code availability)

Reviewer #3

(Remarks to the Author)

In light of antimicrobial resistance, the development of alternative therapy options is mandatory. One aspect that needs to be considered in the process is the emergence of resistance to new antimicrobial substances. In this work, Mulkern and colleages investigate the development of resistance to antimicrobial antisense oligomers (asobiotics). They used peptide nucleic acids (PNAs) conjugated to cell-penetrating peptides (CPPs). In principal, resistance based on mutations of the target sequence can easily be responded to by adapting of the oligo sequence. However, the presence of CPPs introduces

potential new resistance mechanisms related to transport or stress response. The authors employed a laboratory evolution approach, “drug selection ramp”, to systematically compare development of resistance and the underlying genetic variants to several different constructs in four gram-negative pathogens. This is the first study, in which resistance to PNA has been comprehensively investigated. Also, it is the first time that mutations in the asobiotics binding region have been documented following treatment.
The authors found that the number and nature of the observed genetic variants was dependent on the CPP used. Treatment with (KFF)3K-PNAs resulted in a high increase of resistance with a high prevalence of genetic variants in sbmA in all species tested. (KFF)3K-PNA uptake is dependent on SbmA as has been shown before. New resistance determinants that were identified in this work included genetic variants responding to the mode of action of the asobiotics used. (RXR)4XB-PNAs do not use the SbmA route. Treatment with these (RXR)4XB-constructs induced only modest elevation of resistance, partially caused by changes in the target sequence.
Together, in this work was shown that genetic variants leading to asobiotics resistance can be related to transport and the mode of action. The laboratory evolution approach provides information on the route of entry of the conjugates. In addition, the mode of action and specific stress responses can be investigated. In the future, comparison of different constructs will help to design asobiotics that are less prone to resistance development. If resistance could be restricted to target sequence modification, it could be easily circumvented by changing the antisense sequence.
The data have been generated using highly established standard methods, which are sufficiently described in the Material and Methods section. For data robustness, 10 lineages of each individual strain have been generated.
Data processing was documented and the respective scripts are available online. I was able to access the repository, but did not check the individual scripts.
The complex data were efficiently visualized in suitable graphs, making the interpretation of the results feasible for the reader. All figures were accompanied by informative captions. Very useful is the comprehensive presentation of the nature and the distribution of the genetic variations detected in the various strains under the different treatment conditions in the supplemental material). Most conclusions were supported by the data. In some cases, I need some clarification. I am going to address these points specifically as “minor issues”.
Overall, the manuscript is concisely written, the results are well described and discussed with appropriate consideration of previous reports. I do believe that this work has a high significance for readers in this field and I do recommend acceptance with minor modifications.

Minor issues:

All lineages were able to grow throughout the “drug selection ramp” up to 4 x MIC. MIC determination after completion of the experiment resulted in several samples in low resistance increase, e.g. E. coli treated with D-KFF, 1.3 x MIC, indicating a combination of adaption and resistance development. Did you evaluate the post-experimental MICs in the lineages following growth in medium without selection pressure? This would allow to discriminate between the effect of adaption and stable MIC elevation caused by genetic variation. Please give a comment in the text.

Out of curiosity: Did you isolate clones from some of your lineages? If yes, did they all show the respective resistance? How was the genetic variation distributed between the different clones? I you did so: please discuss. If you didn’t, ignore my comment, please.

How did you consider genetic variants caused by medium adaptation? Did you include a mock control or controls featuring a mismatch within the antisense region of the PNA?
Line 458: “we first subtracted variants observed in the corresponding ancestral isolate”.
Here, no subtraction of variants observed in non-treated controls is mentioned. Please explain.

Related to the above question: there is a discrepancy between the occurrence of genetic variants and resistance in D-KFF and RXR constructs.
Lines 234-236: “…hypothesize that the variants observed in a high proportion of samples observed may not be directly related to resistance to these ASO constructs and might represent “passenger” mutations instead.”
How do you differentiate between “passenger” and “resistance” mutations in the samples. The phenomenon might not be restricted to those populations showing a low resistance increase.

Line 436-437: “Plates were incubated for 22 hours at 37°C, and overnight cultures were diluted 1:100 into fresh LB broth medium.”
Did you perform the experimental evolution in LB medium or did you perform the assay analogously to the MIC assay? Please give a more detailed description of the procedure.

(Remarks on code availability)
I was able to access the repository, but did not check the individual scripts, because I am not familiar with the use of such a code.

Version 1:

Reviewer comments:

Reviewer #1

(Remarks to the Author)
Although this paper is presenting an impressive amount of data, no important overall conclusions that may bring this drug discovery field significantly forward are presented.
The major weakness in the study is the fact that the two compounds which are the basis of the study are really not very relevant for the major objective since no in vivo data to the best of my knowledge is presently available. In addition, as the authors indicate themselves, it is rather clear that the KFF peptide is not likely to play any role in future clinical asobiotics; which dramatically reduces the significance of the paper.

Specific points
1) The composition of the two compounds used in the study is not revealed, apart from the delivery peptides: Information on the target sequence length and position of this in the mRNA seems to be missing.
2) It is stated that the PNA compounds were characterized by HPLC analysis and mass spectrometry. This data must be included in supplementary.
3) Asobiotics are defined as antisense oligomers (p. 2 line 64). This would include all antisense compounds, which I believe is not the intention?
4) Page 3 line106-7 & p5 line 178-9: The PNA constructs are not “formulations”!

(Remarks on code availability)

Reviewer #2

(Remarks to the Author)
Thank you for the comprehensive revision and detailed responses to the reviewers. I appreciate that the authors have addressed the principal mechanistic concerns raised through reconstruction of sbmA/yaiW and prfB mutants representing two of the three principal mechanistic classes they identified. Furthermore, clarification of the experimental design, inclusion of the scrambled control evolution experiment, and revisions of Figures 1A and 2 and the addition of statistical analyses improves the clarity and interpretability of the results and substantially strengthen the manuscript. Even though, several of my more specific suggestions (e.g., ykfM mutation and prfA/prfB variants reconstruction and MIC testing) were not pursued experimentally, I accept the authors' rationale that these extensions fall beyond the scope of the present study.

Overall, I find that the manuscript has improved substantially following revision. The central conclusions are now better supported experimentally, and the presentation is considerably clearer. The major concerns raised during review have been satisfactorily addressed.

(Remarks on code availability)

Reviewer #3

(Remarks to the Author)
My main main questions were related to the effects of medium adaption, to the analysis of single clones, and to the inclusion of scrambled controls. All points were satisfactorily addressed in the revised version.

(Remarks on code availability)

Open Access This Peer Review File is licensed under a Creative Commons Attribution 4.0 International License, which permits use, sharing, adaptation, distribution and reproduction in any medium or format, as long as you give appropriate credit to the original author(s) and the source, provide a link to the Creative Commons license, and indicate if changes were made.
In cases where reviewers are anonymous, credit should be given to 'Anonymous Referee' and the source.
The images or other third party material in this Peer Review File are included in the article's Creative Commons license, unless indicated otherwise in a credit line to the material. If material is not included in the article's Creative Commons license and your intended use is not permitted by statutory regulation or exceeds the permitted use, you will need to obtain permission directly from the copyright holder.
To view a copy of this license, visit https://creativecommons.org/licenses/by/4.0/

Reviewer #1

In this study Galardine [*sic*] et al. have analyzed resistance development against antisense BPP-PNAs in four Gram negative bacterial species (E.coli, K. pneumonia, S. enterica and P aeruginosa) by serial passaging evolution, with special focus on the effect of the delivery peptide. The experiments are focussed on acpP as the antisense target, and the well described "KFF" and "RXR" delivery peptides (BPPs).

Apart from the expected resistance from sbmA mutations, a large variety of other gene mutations were identified in the evolved populations. Possible mechanisms by which these may induce resistance either via interference with PNA target binding or inhibition of translation, or by reduction of BPP dependent cellular uptake is discussed, but none of the theories are validated by specific genetic validation or other studies.

Therefore, the study is predominantly descriptive (comprehensive) data presentation.

In addition, although effective bacterial delivery of antisense PNAs and PMOs is a key, activity-limiting process for antisense based antibiotics drug discovery, the relevance of the present study is rather limited in this context, due to the choice of CPPs.

The fundamentally different bacterial uptake processes (in E. coli) for both KFF and RXR PNA conjugates has already been studied identifying the non-essential peptide transporter sbmA as necessary for inner membrane crossing of KFF-PNA (refs. 11 & 13), and its functional absence causing resistance against antisense KFF-PNAs. In contrast, RXR-PNA antisense compounds do not require sbmA for inner membrane crossing, but is dependent on at functional inner membrane potential to drive the crossing, and more than one mutation is required to reduce this potential (ref. 15). Therefore, in a serious drug discovery/development process, the KFF peptide will not be relevant due to the easy resistance development by sbmA mutations. As previously shown (ref 15), and supported by the present data resistance development against RXR conjugates is much more complex and difficult. However, these conjugates may have toxicity issues (e.g. Bioconjugate Chem. 2007, 18, 1325).

Therefore, the present study unfortunately provides limited new knowledge that will guide the antisense antibacterial drug discovery field.

We thank the reviewer for their careful reading of our manuscript, and for raising substantive points regarding mechanistic validation and the scope of our study. We would like to address the reviewers general framing before responding to the specific points raised below.

Firstly, with respect to characterisation of the study as “predominantly descriptive”, the revision now includes direct genetic reconstruction of two representative resistance determinants in a clean MG1655 background, with MIC characterisation (detailed below). These experiments provide causal validation of two principal mechanistic classes proposed in the manuscript, loss of SbmA-dependent uptake and modulation of translation termination/ribosome rescue. Additionally, we performed laboratory evolution experiments using scrambled-sequence PNA controls across the strain and CPP panel, which provides orthogonal evidence that the resistance determinants that we identified reflect antisense-specific selection rather than generic peptide-mediated or media-adaptation effects. We believe that with these additions, the study moves substantially beyond descriptive data presentation to provide both characterisation and mechanistic validation of resistance determinants. We note that we also attempted to generate

a mutant with one of the observed mutations in the acpP-PNA binding site, but despite multiple attempts at isolating the clone and generating the mutant, we did not succeed. We believe that these failures indicate 1) that acpP expression levels cannot be altered without causing cell lethality, and 2) that the mutations we identified likely have a substantial fitness effect. Both hypotheses indirectly validate the acpP mutations we have identified.

Secondally, with respect to the relevance of the study to antisense antibacterial discovery, the reviewer correctly notes that KFF is not a likely candidate clinical development due to its susceptibility to rapid resistance via *sbmA* loss-of-function, and that RXR presents a more robust delivery option against resistance. We agree with both points. Our study, however, was not designed as a direct comparison of CPPs but as a systematic characterisation of the resistance landscape across delivery strategies and bacterial species. In the framing, KFF serves as a valuable comparator precisely because its resistance mechanisms are well-defined and provide a benchmark against which other CPP formulations can be assessed. The comparison across CPPs shows that CPP choice is a principal determinant of resistance evolvability, with RXR based delivery showing lower evolvability of resistance across species. The systematic identification of non-*sbmA* resistance determinants further contributes to the field by mapping the alternative genetic routes available to bacteria under asobiotic selection, which is directly informative for designing future formulations.

Finally, we note that the mechanistic reconstructions now included in this revision provide the first direct evidence that ribosome-level adaptation can contribute to asobiotic resistance independent of CPP-uptake. This is a mechanistically novel observation that, to our knowledge, has not been previously reported, and argues for a broader view of asobiotic resistance than just uptake-focused framing.

We address the reviewers' specific points below.

Other points

1. The more detailed mechanistic interpretation of the genetic data is limited by the lack of data from isolated resistant clones, and lack of data on the effect of mutations in single genes and in specific combinations of genes.

We would like to thank the reviewer for this important comment, which aligns with a related suggestion from Reviewer #2 regarding genetic validation of candidate resistance determinants. To address both sets of concerns, we isolated single clones from selected evolved populations and set out to reconstruct three candidate resistance determinants in a clean MG1655 background, each representing a mechanistically distinct class emerging from our population-level data, including loss-of-function of the *sbmA/yaiW* operon, predicted to impair CPP-dependent PNA uptake, the *prfB* non-synonymous variant, predicted to affect ribosome rescue and translation termination and a non-coding, target-site SNP within the *acpP* PNA binding window, predicted to act at the level of PNA binding.

We were able to reconstruct two of these three determinants. We isolated a clone from M08 carrying a deletion spanning *sbmA* and *yaiW*, and a clone from M02, carrying the *prfB* SNP, from their respective KFF-*acpP* evolved populations. Each variant was reconstructed

independently in the ancestral MG1655 background using a modified Lambda-Red/I-SceI allelic replacement method , with *prfB* (an essential gene in *E. coli*) reconstructed via a hybrid Lambda-Red/P1 co-transduction strategy at the linked *idi* locus. Full details of the mutant reconstructions are now provided in the Methods (lines 510-536).

MIC assays with KFF-acpP confirmed that both variants are sufficient to confer resistance in isolation. The reconstructed *sbmA*/*yaiW* deletion mutant showed an MIC of >80 μM (highest concentration tested), a >16-fold increase over the ancestral MG1655 MIC of 5 μM, consistent with loss of KFF-PNA inner membrane translocation. The reconstructed *prfB* T246S mutant displayed an MIC of 20 μM, a 4-fold increase over the ancestral MIC, confirming that a single substitution in a ribosome rescue factor confers measurable resistance in the absence of any uptake-associated mutation. These reconstructions confirm that the two principal mechanistic classes we propose, loss of the SbmA-dependent uptake route and modulation of translation termination, are each individually sufficient to drive measurable KFF-acpP resistance. The data are now presented in a new Results subsection 'Genetic reconstruction confirms two mechanistically distinct routes to KFF-acpP resistance' (lines 344-367). For the third determinant, we attempted reconstruction of the *acpP* target-site SNP in collaboration with the Uhlemann laboratory (Columbia University, New York) but were unable to obtain a stable reconstructed mutant, which we attribute to *acpP* dosage sensitivity (Discussion, lines 435-439). While we were therefore unable to validate this class directly, we consider the outcome itself informative. The difficulty of recovering viable taerget-site mutant is consistent with the fitness-cost hypothesis discussed in the manuscript, and with the correspondingly low frequency of these variants in our evolved populations.

Together, these reconstructions provide direct functional support for two of the three principal mechanistic classes we identify, and the unsuccessful *acpP* attempt is consistent with the predicted cost of target-site mutations. We believe this substantially strengthens the mechanistic interpretation of our population-level data, in line with the reviewer's comment.

2. KFF- and RXR-PNA conjugates can also exhibit gene target independent, direct antibacterial effects analogous to those of antibacterial peptides, as observed by antibacterial activity of e.g. mismatch controls (at higher concentration). Thus inclusion of such mismatch controls could shed light on this mechanism.

We thank the reviewer for raising this important point. We agree that distinguishing peptide-mediated antibacterial effects is critical for interpreting our resistance data. We performed a parallel laboratory evolution experiment using scrambled-sequence PNAs conjugated to each of the four CPPs (KFF, D-KFF, and RXR), applied under identical drug selection ramp conditions across the same panel of strains used in the main experiments.

Two observations from the scrambled-control experiment support the interpretation that selection in our main experiments acted on the antisense mechanism rather than on generic CPP-mediated mechanisms.

First, growth across the 16-passage selection ramp remained stable for all scrambled-PNA conditions (Sup fig 2), indicating that the scrambled conjugates do not impose sufficient selective pressure under our experimental conditions. Second, whole genome sequencing of the endpoint scrambled-PNA bulk populations recovered none of the key resistance determinants identified in the main evolution experiments, with the exception of fimE (Sup fig 3).

We did not detect variants in *sbmA* or *yaiW* (principal KFF-PNA uptake determinants), in *ykfM*

(selected in MG1655 and Δ*sbmA* lineages against KFF-acpP), or in *prfB*/*prfA* (the ribosome rescue factors). The variants we did identify in the scrambled-control lineages were predominantly genes associated with media or passaging adaptation, including *fimE* and *fimA* (linked to cell adhesion and bacterial attachment), flagellar, motility and chemotaxis (*flhA/C/D*, *motA*/*B*, *cheW/Y*/*B* etc) (Sup fig 3).

Together, these data indicate that the resistance determinants reported in the main experiments, particularly variants in *sbmA*/*yaiW*, *prfB*/*prfA*, and target-site SNPs within the *acpP* binding window, reflect selection on the antisense mechanism rather than the independent peptide effect.

3. Line 78: PMOs do not have a pseudopeptide backbone
We thank the reviewer for catching this error, we have revised the sentence in the Introduction (line 85-86) to correctly detail the two PNA and PPMO modalities.

Text now reads "PNAs are stabilized and protected from nuclease degradation by a neutrally charged pseudo-peptide backbone, while PPMOs are based on a phosphorodiamidate morpholino backbone that resists nuclease degradation."

4. Line 95-96: SbmAI want does not confer resistance to KFF conjugates, on the contrary, it is required for activity. Indeed the argumentation in lines 94-99 does not make much sense.
We thank the reviewer for pointing out this ambiguity and we apologise for the unclear phrasing. We have revised the Introduction (lines 102-106) to reflect this distinction.

Text now reads "Spontaneous mutations affecting active uptake have instead been previously described, specifically loss-of-function mutations in *sbmA*, which encodes an inner membrane transporter required for the uptake of $(KFF)_3$K-conjugated PNAs and PPMOs into bacterial cells; loss of SbmA function therefore renders bacteria less susceptible to these formulations by preventing uptake[10,11]."

5. Supplementary tables are missing
We have included these tables in the submission

Reviewer #2
Mulkern et al. aim to investigate the mechanisms of resistance to antisense peptide nucleic acids (PNAs) through in vitro laboratory evolution and genome sequencing of the evolved clones.

<u>Experimental Design and Key Objectives:</u>
Primary Resistance Mechanism:
To investigate the primary resistance mechanism against a $(KFF)_3$-K–conjugated PNA targeting acpP (KFF-acpP), the authors evolved 10 biological replicates from three Gram-negative pathogens: Escherichia coli (UPEC), Klebsiella pneumoniae, and Salmonella enterica and a lab E. coli strain (MG1655) under selective pressure with KFF-acpP.

Identifying Additional Genetic Factors:
To identify genes influencing PNA activity beyond the well-known role of sbmA (the transporter responsible for $(KFF)_3$-K–conjugated PNA uptake), an sbmA knockout strain (ΔsbmA) was evolved against the same $(KFF)_3$-K–conjugated PNA targeting acpP.

Impact of Different CPPs on Resistance:
To examine how different cell-penetrating peptides (CPPs) affect PNA resistance and evolvability, a laboratory E. coli strain (MG1655) was evolved against acpP PNA conjugated with three distinct CPPs: the original L-form of $(KFF)_3$-K, its D-form (D-$(KFF)_3$-K), and $(RXR)_4$XB.

Comparing Resistance Mechanisms for Different PNAs:
Finally, to compare resistance mechanisms, the authors evolved a laboratory E. coli strain against two PNAs targeting acpP and rpsH, both conjugated with $(KFF)_3$-K.

<u>Key Findings:</u>
Mutations in sbmA and yaiW:
Loss-of-function mutations in the sbmA gene—a known mediator of $(KFF)_3$-K–conjugated PNA uptake—were common across all three tested Gram-negative pathogens. Additionally, yaiW was identified as a potential contributor to $(KFF)_3$-K-PNA uptake. Notably, yaiW is co-transcribed with sbmA and a palmitate-modified, surface-exposed outer membrane lipoprotein.

Species-Specific Mutations:
Other potentially species-specific mutations were observed following treatment with $(KFF)_3$-K–conjugated acpP, such as mutations in rfbF gene of S. enterica lines (which may impact CPP binding and penetration) and in mdtC gene of the K. pneumoniae lines (a component of the mdtABC/tolC multidrug efflux pump).

Role of ykfM:
Variants in the uncharacterized transmembrane gene ykfM were consistently identified across all replicates of both MG1655 and ΔsbmA strains adapted to $(KFF)_3$-K–acpP. Therefore, the authors suggest that ykfM indirectly affects the efficacy of the PNA targeting acpP.

Delivery Method Influences Resistance:
The study demonstrates that the delivery method is crucial; the acpP PNA conjugated with $(KFF)_3$-K showed a higher evolution of resistance compared to conjugates with D-$(KFF)_3$-K or $(RXR)_4$XB.

Ribosome Rescue Mechanisms:
In a highly resistant sample (16-fold MIC increase for KFF–acpP), a mutation (T246S) in the prfB gene was identified. Similarly, a variant in the related prfA gene was observed in an evolved UPEC strain. Since both genes are involved in rescuing stalled ribosomes, the authors propose two potential hypotheses for the cellular response to PNAs.

Rare Mutation Within The PNA Target Window
For RXR–acpP, only a relatively low-frequency (~30%) set of SNPs was identified within the core of the PNA target window. This represents the first evidence that rare mutations within this central binding region may reduce the efficacy of the PNA.

Major General Comments:
- Clarity and Logical Flow: The paper is challenging to follow due to its lack of clear logical flow. Since not all possible combinations of CPP-PNAs and bacterial strains were screened, the specific objectives of each evolution experiment remain unclear. I recommend reorganizing the paper around the four main experimental goals (as outlined above) to better clarify the purpose and provide a more focused discussion of the genetic variants observed.

Given that we now have followed the reviewer's suggestion to improve Figure 1A, we believe that the experimental design is now more clearly presented. As a result the general flow of the manuscript also has been improved. The logical flow we had decided to take was to first start with the phenotypic change induced by adaptation (fold-changes of MICs), then followed by listing common mutations first, and rare mutations second. We have now added a section with the validation of one common (*yaiW*) and one rare (*prfB*) mutation, which we think nicely follows our desired flow. We think that dramatically restructuring the manuscript would not necessarily change the key messages we are conveying, while imposing a significant burden on the main author, who has since moved to a new lab and who has therefore significantly less time to dedicate to writing.

- Additionally, the authors might consider reintroducing some of the most interesting and novel genetic variants into wild-type lab E. coli strains to further support their observations and hypotheses.

We thank the reviewer for this suggestion and one that aligns with a similar comment from Reviewer 1 regarding mechanistic interpretation of our genetic data. To address these concerns, we isolated single clones from selected evolved populations and reconstructed two candidate mutations (*sbmA*/*yaiW* deletion mutant, and a *prfB* T246S SNP) in a clean MG1655 background. MIC assays against KFF-*acpP* confirmed that both variants are sufficient to confer resistance in isolation. Full details of the reconstruction strategy and MIC results are provided in our response to Reviewer 1 (Other points 1), in the results (344-367) and described in the Methods (lines 511-536)

Figure 1A:
Figure 1A is somewhat misleading, as it implies that all listed strains were evolved with all CPP-PNA combinations. A table that clearly details which evolution experiments were performed for each strain and CPP-PNA combination would be very helpful.

We thank the reviewer for pointing out the lack of clarity deriving from this figure panel. We have updated the panel to clearly indicate which combinations of species/strains were tested with

which asobiotic, including the scrambled controls. We believe that the experimental design is now sufficiently clear.

Selection Pressure Concerns:
The experimental design (starting at 0.5 MIC and increasing by 2-fold every fourth step over 16 passages) and the high frequency of synonymous mutations (an average of 15 SNPs per sample, lines 187-189) suggest that the selection pressure might have been relatively mild. This could lead to a higher frequency of neutral mutations and might not fully capture the genetic adaptations that would occur under stronger, more clinically relevant conditions. The authors should discuss how this might affect the robustness of their conclusions.

We thank the reviewer for raising this important methodological point. We would like to address two aspects, the choice of selection regime and the interpretation of neutral variant frequencies in our dataset.

Our drug selection ramp design, starting at 0.5xMIC and doubling every four passages to reach 4xMIC by passage 16, is adapted from a widely used laboratory evolution 'drug-selection ramp' approach to map resistance determinants, which are cited in our manuscript e.g. Gifford *et al.* (2018) and San Milan *et al.* (2016). This design was deliberately chosen to provide a sustained selection pressure while also avoiding narrow genetic bottlenecks or population extinctions that can occur under high-concentration challenges. Reach 4xMIC at the final passages represents a substantial, not a mild, selection pressure and the magnitude of resistance phenotypes we observe (with relatively large MIC fold-changes across biological replicates) confirms that the regime was sufficient to drive meaningful adaptation. We nonetheless agree with the reviewer that the choice of selection regime can shape which resistance determinants are recovered. To address this directly, we have added a sentence to the Discussion (lines 446-449) noting that the spectrum of determinants we observe is conditioned on our gradual ramping regime, and that alternative regimes, such as the abrupt high-concentration exposure characteristic of some clinical scenarios, might favour a different distribution of mutational routes.

With respect to the frequency of synonymous and other potentially neutral mutations, we agree with the reviewer that lab evolution experiments inherently accumulate 'passenger' variants alongside other variants under selection, and distinguishing between them is a common analytical challenge. Our analysis pipeline addresses this through multiple filtering steps. First, we subtract variants present in the corresponding ancestral isolate from each adapted sample. Second, we exclude synonymous SNPs and other variants in intergenic regions or those in known pseudogenes from downstream analysis. Thirdly, we apply a 15% frequency threshold in variant calling as an additional filter of mutations. And lastly, we identified resistance determinants that emerge in parallel across independent biological replicates, representing a more robust statistical signature of selection under identical conditions.

Further evidence that our selection regime recovers genuine antisense-specific resistance signals rather than neutral drift comes from our parallel scrambled-sequence PNA evolution experiments, now included in this revision (see reviewer 1 response, and new sup fig 3). Under

identical ramping conditions scrambled-control lineages recovered none of the principal resistance determinants identified in the main experiments i.e. no variants in *sbmA*, *yaiW*, *ykfM*, *prfB/prfA*, and SNPs in the *acpP* binding window. Other genes associated with media and passaging adaptation i.e. *fimE* and *fimA*, demonstrate that our selection does generate passenger mutations, but ones that are clearly distinguishable from the antisense-specific resistance markers recovered from the targeted experiments.

Role of fimE:
The authors note that fimE, involved in fimbrial biosynthesis, was frequently mutated in E. coli MG1655 adapted to all KFF-conjugated PNAs and in K. pneumoniae evolved to KFF–acpP. However, they overlook that fimE was also the most frequently mutated gene in lab E. coli adapted to D-KFF–acpP (as shown in Figure 3B), but not in strains evolved with RXR–acpP. Could the authors provide further insight into whether fimE might play a role in the uptake of KFF and D-KFF conjugated PNAs?

We thank the reviewer for this interesting observation. The parallel scrambled-sequence PNA evolution experiments now included in this revision (reviewer 1 comment response + sup fig 3) indicate that *fimE* has a CPP-independent effect under the passaging regime, as *fimE* variants were recovered in *E.coli* MG1655 lineages evolved against all three scrambled-sequence controls (KFF, D-KFF, and RXR), consistent with the established advantage conferred by *fim* genes in shaken cultures.

We do acknowledge that the RXR-*acpP* main evolution experiment recovered other adaptive variants (flagellar, motility and chemotaxis loci), indicating that this condition permitted detectable accumulation of growth-adapive mutations, but that specific set differs from the KFF-conjugated lineages. The recovery of fimE variants across all three scrambled-sequence controls indicates that fimE selection reflects the passaging regime rather than asobiotic activity, and we therefore do not consider it a determinant of asobiotic resistance.

<u>Additional Comments:</u>
yaiW: Why was yaiW frequently mutated only in the lab E. coli strain (MG1655) evolved to $(KFF)_3$-K–acpP or $(KFF)_3$-K–rpsH and not in other Gram-negative pathogens evolved to $(KFF)_3$-K–acpP?

We believe that yaiW plays a role in providing resistance to asobiotics; the *sbmA*/*yaiW* double mutant has in fact up to a 2-fold increase with respect to evolved lines that only have a *sbmA* mutation. We do recognize that we only observed this in *E. coli* MG1655, likely because of the higher propensity to acquire mutations via large deletions around the *sbmA* region. We have added a sentence in the Discussion (lines 393-397) to indicate how this aspect will need to be clarified in future studies.

Species-Specific Mutations: Are the observed mutations in rfbF and mdtC truly species-specific, given previous links to reduced PNA efficacy in E. coli (lines #224–227, #362–364)?

We believe that we have not made any claims that mutations in this genes are specific to Salmonella and Klebsiella, and we have simply described that we have observed them in those two species. We have therefore not altered the manuscript in response to this comment.

Resistance Comparison against acpP and rpsH PNAs: Can the authors provide any further explanation for why the laboratory E. coli strain (MG1655) developed lower resistance to $(KFF)_3$K–rpsH compared to $(KFF)_3$K–acpP?

We thank the reviewer for bringing this observation up, which is indeed interesting. While we cannot validate this hypothesis with the data we have generated, we can speculate that it could be the combination in differences in efficacy between the two PNAs, and in the lower number of mutations induced by KFF-rpsH. Given the very speculative nature of this hypothesis, we have elected to simply point out the overall difference in fold change between E. coli MG1655 adapted to KFF-acpP Vs. KFF-rpsH (lines 183-186).

ykfM: The authors suggest that the uncharacterized transmembrane gene ykfM indirectly affects the efficacy of PNA targeting acpP. However, it is notable that ykfM mutations were observed only in the laboratory E. coli strains and not in other Gram-negative pathogenic strains. Could the authors provide any further explanation for this observation? Reintroducing the ykfM mutation into a wild-type lab E. coli background and assessing its resistance to both $(KFF)_3$-K–acpP and $(KFF)_3$-K–rpsH would greatly strengthen this hypothesis

With respect to the reviewers suggestion of *ykfM* reconstruction in a wild-type *E.coli* background, we agree that direct functional validation would further strengthen the mechanistic interpretation of *ykfM*. Particularly, in light of its recovery across MG1655 and *ΔsbmA* lineages. However, the three reconstructions we have performed for this revision (*sbmA/yaiW* deletion and *prfB* T246S, and a non-coding SNP in acpP; see our response to Reviewer 1), were selected specifically to validate two principal mechanistic classes emerging from our population-level data i.e. loss of CPP-dependent uptake, modulation of translation termination and ribosome rescue, and PNA binding. We consider a broader reconstruction panel extending to *ykfM* as an important direction for future work, but is outside the scope of the present study to map the resistance determinants across CPP-PNA combinations and bacterial species rather than dissecting each determinant in depth.

prfA/prfB Mutations: The authors propose that mutations in prfA and prfB (which rescue stalled ribosomes) may confer resistance to PNAs. Are these resistance mechanisms independent of PNA or CPP? Reintroducing these mutations into appropriate strain backgrounds and testing resistance against different KFF-conjugated PNAs (e.g., acpP vs. rpsH, or D-KFF–acpP vs. RXR–acpP) would provide valuable insights.

We would like to thank the reviewer for the insightful suggestion. We predict that the resistance contribution from prfA and prfB is largely CPP-independent due to their action on the ribosome after PNA uptake has already occurred. If the CPP governs delivery to the cytoplasm, the *prfA/prfB* variants would act downstream of the delivery by modulating translation termination and ribosome rescue. In contrast, the contribution of these variants is more likely to show PNA-target dependence.

Our reconstruction experiments, now included in this revision (response to reviewer 1), directly support the first part of this prediction. The reconstructed *prfB* T246S mutant confers resistance to KFF-*acpP* in isolation (MIC 20 μM, 4-fold over the ancestral) in a clean MG1655 background, without any uptake-associated variant. This confirms that the *prfB* T246S resistance mechanism does not require CPP-specific context to operate.

We agree with the reviewer that this broader reconstruction panel, extending to *prfA* and testing across multiple CPP-PNA combinations, would enrich the mechanistic picture. As noted in our response to the reviewer's earlier comments on reconstruction and on ykfM, we consider this an important direction for future work but outside the scope of the present study.

RXR Binding Site Mutations: For P. aeruginosa, did the authors find any mutations at the center of the RXR–acpP binding site among the evolved strains, which exhibited a 2-fold increase in MIC?
We have double checked the sequencing data (**Supplementary Table 2**, and **Supplementary Figures 10** and **14**), and we can confirm that no variants were induced in the RXR-acpP binding site, nor in the vicinity of the acpP gene.

<u>Comments on Figure 2:</u>
Clarity and Data Presentation: Figure 2 could be improved for better clarity. Several data points overlap, making it difficult to distinguish individual results. Applying jittering on both axes would help separate these points and enhance visibility. Additionally, reorganizing the data could improve readability. It would be helpful to separate the results for pathogenic strains treated with KFF–rpsH and RXR–acpP (e.g., for P. aeruginosa) from those for laboratory E. coli strains (MG1655 and ΔsbmA) treated with various CPP-PNAs. Currently, the results for the lab E. coli and UPEC strains appear in both the left and right panels, which can be confusing. Grouping similar data together in dedicated panels would enhance clarity. Furthermore, indicating which pathogen strains evolved statistically significantly higher or lower resistance to KFF–acpP, as well as highlighting which CPP-PNAs induced significantly stronger or lower resistance in the lab E. coli strains, would provide valuable insights and make the figure more informative.

We thank the reviewer for these further suggestions to improve the presentation and clarity of our results. We have implemented both suggestions. In particular, we have updated Figure 2B to separate the lab strains from the pathogenic ones, and we have split the distributions so that they do not overlap. We have also ran a series of one-sample Wilcoxon signed-rank tests to

identify which fold-change distributions were significantly higher that the null (i.e. no increase in MIC), and pairwise Mann-Whitney U tests between the E. coli MG1655 sets to identify which were significantly different from each other. We have reported the results of these tests in the manuscript (lines 165-187).

Conclusion:
Overall, the reported results are both important and novel, given the current lack of empirical data on the adaptation strategies to asobiotic treatments across bacterial pathogens. Addressing the points above—particularly clarifying the experimental design, reorganizing the presentation of data (especially for Figures 1A and 2), and further exploring specific genetic variants (such as those in ykfM and prfB, prfA)—would significantly enhance the manuscript.

We thank the reviewer for their overall positive assessment of the work and for the constructive set of comments throughout the review. In this revision, we have clarified the experimental design by revising Figure 1A to indicate which species and asobiotic combinations were tested, including the scrambled-sequence controls, and provided functional validation of two of the principal resistance determinants (*sbmA/yaiW* and *prfB*) through reconstruction in a clean MG1655 background. We have also addressed the reviewers specific questions regarding *ykfM, prfA,* and additional genetic variants. We believe these changes substantially strengthen the manuscript and trust the reviewer agrees.

Reviewer #3
In light of antimicrobial resistance, the development of alternative therapy options is mandatory. One aspect that needs to be considered in the process is the emergence of resistance to new antimicrobial substances. In this work, Mulkern and colleages investigate the development of resistance to antimicrobial antisense oligomers (asobiotics). They used peptide nucleic acids (PNAs) conjugated to cell-penetrating peptides (CPPs). In principal, resistance based on mutations of the target sequence can easily be responded to by adapting of the oligo sequence. However, the presence of CPPs introduces potential new resistance mechanisms related to transport or stress response. The authors employed a laboratory evolution approach, “drug selection ramp”, to systematically compare development of resistance and the underlying genetic variants to several different constructs in four gram-negative pathogens. This is the first study, in which resistance to PNA has been comprehensively investigated. Also, it is the first time that mutations in the asobiotics binding region have been documented following treatment.
The authors found that the number and nature of the observed genetic variants was dependent on the CPP used. Treatment with (KFF)3K-PNAs resulted in a high increase of resistance with a high prevalence of genetic variants in sbmA in all species tested. (KFF)3K-PNA uptake is dependent on SbmA as has been shown before. New resistance determinants that were identified in this work included genetic variants responding to the mode of action of the asobiotics used. (RXR)4XB-PNAs do not use the SbmA route. Treatment with these (RXR)4XB-constructs induced only modest elevation of resistance, partially caused by changes in the target sequence.
Together, in this work was shown that genetic variants leading to asobiotics resistance can be

related to transport and the mode of action. The laboratory evolution approach provides information on the route of entry of the conjugates. In addition, the mode of action and specific stress responses can be investigated. In the future, comparison of different constructs will help to design asobiotics that are less prone to resistance development. If resistance could be restricted to target sequence modification, it could be easily circumvented by changing the antisense sequence.
The data have been generated using highly established standard methods, which are sufficiently described in the Material and Methods section. For data robustness, 10 lineages of each individual strain have been generated.
Data processing was documented and the respective scripts are available online. I was able to access the repository, but did not check the individual scripts.
The complex data were efficiently visualized in suitable graphs, making the interpretation of the results feasible for the reader. All figures were accompanied by informative captions. Very useful is the comprehensive presentation of the nature and the distribution of the genetic variations detected in the various strains under the different treatment conditions in the supplemental material). Most conclusions were supported by the data. In some cases, I need some clarification. I am going to address these points specifically as "minor issues".
Overall, the manuscript is concisely written, the results are well described and discussed with appropriate consideration of previous reports. I do believe that this work has a high significance for readers in this field and I do recommend acceptance with minor modifications.

<u>Minor issues:</u>
All lineages were able to grow throughout the "drug selection ramp" up to 4 x MIC. MIC determination after completion of the experiment resulted in several samples in low resistance increase, e.g. E. coli treated with D-KFF, 1.3 x MIC, indicating a combination of adaption and resistance development. Did you evaluate the post-experimental MICs in the lineages following growth in medium without selection pressure? This would allow to discriminate between the effect of adaption and stable MIC elevation caused by genetic variation. Please give a comment in the text.

We thank the reviewer for the important methodological point. Our MIC assays were performed on overnight cultures derived from endpoint evolved populations following a single passage in drug-free medium, this is standard for experimental evolution endpoint MIC determination. However, it does not fully exclude the possibility that lower relative MIC increases reflect transient phenotypic adaptation rather than stable genetic resistance. We did not perform additional extended drug-free passaging prior to MIC testing to discriminate between these possibilities.

Yet, for the lineages that form the basis of our central conclusions, the resistance phenotype is unambiguously genetic. The high-resistance KFF-conjugate treated lineages showed consistent genetic variants across biological replicates (e.g. *sbmA*, *yaiW*), and the mutant reconstructions now included in the revision (see responses to reviewer 1) directly demonstrate that the reconstructed *sbmA/yaiW* deletion and *prfB* T246S variants confer resistance in clean MG1655 backgrounds. With MIC increases that fall in the range observed in evolved populations.

Out of curiosity: Did you isolate clones from some of your lineages? If yes, did they all show the respective resistance? How was the genetic variation distributed between the different clones? I you did so: please discuss. If you didn't, ignore my comment, please.

We thank the reviewer for the interesting comment. For this revision, we isolated single clones from two KFF-*acpP* evolved *E. coli* MG1655 populations for genetic reconstruction (see our response to Reviewer 1). Both clones carried the candidate resistance variant identified in the parent population (Δ*sbmA*/*yaiW* and *prfB* T246S) and showed the corresponding resistance phenotype. We did not perform systematic clone-level characterisation across the full panel of evolved lineages, as our analytical strategy relied on population-level whole-genome sequencing.

How did you consider genetic variants caused by medium adaptation? Did you include a mock control or controls featuring a mismatch within the antisense region of the PNA?

We thank the reviewer for raising this point. For this revision, we performed a parallel laboratory evolution experiment using scrambled-sequence PNAs conjugated to each CPP under identical drug selection ramp conditions (see extended response to reviewer 1). These experiments serve precisely the function the reviewer describes, allowing us to distinguish genetic variants arising from media and passaging adaptation from those specifically selected by antisense activity. Scrambled-PNA lineages recovered none of the principal resistance determinants identified in the main experiments, while variants that did accumulate were predominantly genes associated with liquid-culture passaging adaptation. This evidence directly supports the interpretation that the resistance determinants we report reflect antisense-specific selection rather than media-driven.

Line 458: "we first subtracted variants observed in the corresponding ancestral isolate".
Here, no subtraction of variants observed in non-treated controls is mentioned. Please explain.

For this revision, we have added parallel laboratory evolution experiments using scrambled-sequence PNAs conjugated to each CPP under identical drug selection ramp conditions. These experiments provide a matched non-targeted control at the full evolution-experiment scale and allow us to directly distinguish media and passaging adaptation from antisense-specific selection.

Related to the above question: there is a discrepancy between the occurrence of genetic variants and resistance in D-KFF and RXR constructs.
Lines 234-236: "…hypothesize that the variants observed in a high proportion of samples observed may not be directly related to resistance to these ASO constructs and might represent "passenger" mutations instead."
How do you differentiate between "passenger" and "resistance" mutations in the samples. The

phenomenon might not be restricted to those populations showing a low resistance increase.

We thank the reviewer for this interesting and important point. The reviewer is correct that the distinction between passenger and resistance mutations is not restricted to lineages with lower MIC increase, and our framing could be improved slightly (Results 260-262).

First, we can use condition-specific emergence, established by our comparison with scrambled controls. The parallel laboratory evolution experiments using scrambled-sequence PNAs included in this revision provide a direct empirical baseline for variants selected by passaging and other non-PNA specific effects rather than antisense activity. Secondly, via direct functional validation through mutant reconstruction for a selection. These reconstructions included in this revision, provide evidence for the resistance phenotype of two specific variants were in fact sufficient to confer resistance in a clean MG1655 background. And thirdly, mechanistic plausibility and correlation with phenotype. For the variants not amendable for reconstruction we can assess their consistency between the predicted mechanism of action and the observed resistance phenotype. For example, the *acpP* target-site SNPs, fall within the central PNA binding site and disrupt this process. When exactly the two RXR-*acpP* lineages with the SNPs showed elevated MIC, this supports the interpretation that resistance mutations (despite their sporadic distribution) disrupt the binding interaction.

Line 436-437: “Plates were incubated for 22 hours at 37°C, and overnight cultures were diluted 1:100 into fresh LB broth medium.”
Did you perform the experimental evolution in LB medium or did you perform the assay analogously to the MIC assay? Please give a more detailed description of the procedure.

We thank the reviewer for raising this point. The experimental evolution and MIC determination were two distinct procedures performed under different conditions, and we have revised the Methods to make this explicit (additional lines added in Methods ‘Experiment Evolution’ subsection).

The experimental evolution ‘drug selection ramp’ was performed in LB broth in 96-well microplates, with 150 µl per well and 10 biological replicates per strain and asobiotic combination. Plates were incubated at 37 °C for 22h per passage, and at the end of the passage OD600nm was measured before serial transfer by 1:100 dilution into fresh LB broth containing relevant asobiotic concentration for next passage. Asobiotic concentrations were increased every four passages from 0.5xMIC to 4xMIC over 16-day passage ramp. We selected LB as the evolution medium for a few reasons. Firstly, LB is the standard rich medium for laboratory evolution experiments across gram-negative species and supported robust reproducible growth of the four pathogens. Secondly, LB supports higher bacterial population densities than MHB, which increases effective population supply and mutation supply available to selection each passage, improving our sampling of the fitness landscape and recovery of

lower probability/lower frequency resistance determinants across replicate lineages. The asobiotic formulations induced MIC increases in adapted populations and the parallel selection of expected CPP-antisense specific resistance determinants.

MIC determination was performed in MHB, a standardised susceptibility-testing medium, following modified broth microdilution protocol in accordance with CLSI guidelines. MHB was used in our manuscript for MIC determination to ensure resulting susceptibility values are directly comparable with published literature.

<u>Remarks on code availability:</u>
I was able to access the repository, but did not check the individual scripts, because I am not familiar with the use of such a code.

Reviewer #1

Although this paper is presenting an impressive amount of data, no important overall conclusions that may bring this drug discovery field significantly forward are presented.
The major weakness in the study is the fact that the two compounds which are the basis of the study are really not very relevant for the major objective since no in vivo data to the best of my knowledge is presently available. In addition, as the authors indicate themselves, it is rather clear that the KFF peptide is not likely to play any role in future clinical asobiotics; which dramatically reduces the significance of the paper.

We acknowledge that the reviewer has reservations about the significance of our study. In our opinion, the study's strengths are the description and validation of new PNA resistance variants (*prfB* in particular), and the low frequency of resistance for PNAs that don't use the KFF delivery peptide. These insights will be the base for future studies in the field, for which we anticipate the possibility to advance the development of this yet untapped antimicrobial drug class.

Specific points

1) The composition of the two compounds used in the study is not revealed, apart from the delivery peptides: Information on the target sequence length and position of this in the mRNA seems to be missing.
We have added all missing information in the new **Supplementary Table 4**. We apologies for not having provided this information earlier.

2) It is stated that the PNA compounds were characterized by HPLC analysis and mass spectrometry. This data must be included in supplementary.
We have added the HPLC and immediately available MS results for the PNAs used in this study as a new **Supplementary Data File 1**, using the IDs reported in the new **Supplementary Table 4**. We note that obtaining the MS spectra from the suppliers' records for some of the PNAs would require incurring in additional delays; given the high purity indicated by the HPLC data, which are available for all PNAs, we believe that including those would not be required.

3) Asobiotics are defined as antisense oligomers (p. 2 line 64). This would include all antisense compounds, which I believe is not the intention?
We have clarified the definition of asobiotics on page 2, which now reads: "In the context of antimicrobial therapy, asobiotics are a subclass of ASOs that are developed as antibacterials, designed to inhibit bacterial growth by targeting the mRNA of essential genes with sequence complementarity to the translation initiation region."

4) Page 3 line106-7 & p5 line 178-9: The PNA constructs are not "formulations"!
We have changed the terminology throughout the manuscript, avoiding using the term "formulation".

Reviewer #2:

Thank you for the comprehensive revision and detailed responses to the reviewers. I appreciate that the authors have addressed the principal mechanistic concerns raised through

reconstruction of sbmA/yaiW and prfB mutants representing two of the three principal mechanistic classes they identified. Furthermore, clarification of the experimental design, inclusion of the scrambled control evolution experiment, and revisions of Figures 1A and 2 and the addition of statistical analyses improves the clarity and interpretability of the results and substantially strengthen the manuscript. Even though, several of my more specific suggestions (e.g., ykfM mutation and prfA/prfB variants reconstruction and MIC testing) were not pursued experimentally, I accept the authors' rationale that these extensions fall beyond the scope of the present study.

Overall, I find that the manuscript has improved substantially following revision. The central conclusions are now better supported experimentally, and the presentation is considerably clearer. The major concerns raised during review have been satisfactorily addressed.

We are glad to have produced an improved manuscript.

Reviewer #3:
My main main questions were related to the effects of medium adaption, to the analysis of single clones, and to the inclusion of scrambled controls. All points were satisfactorily addressed in the revised version.

We are glad to have produced an improved manuscript.
